# Machine learning–based feature selection to search stable microbial biomarkers: application to inflammatory bowel disease

**DOI:** 10.1093/gigascience/giad083

**Published:** 2023-10-26

**Authors:** Youngro Lee, Marco Cappellato, Barbara Di Camillo

**Affiliations:** Department of Electrical and Computer Engineering, Seoul National University, Seoul, 08826, Korea; Institute of Engineering Research at Seoul National University, Seoul, 08826, Korea; Department of Information Engineering, University of Padova, Padova, 35122, Italy; Department of Information Engineering, University of Padova, Padova, 35122, Italy

**Keywords:** microbiota, machine learning, feature selection, biomarkers discovery, Shapley values

## Abstract

**Background:**

Biomarker discovery exploiting feature importance of machine learning has risen recently in the microbiome landscape with its high predictive performance in several disease states. To have a concrete selection among a high number of features, recursive feature elimination (RFE) has been widely used in the bioinformatics field. However, machine learning–based RFE has factors that decrease the stability of feature selection. In this article, we suggested methods to improve stability while sustaining performance.

**Results:**

We exploited the abundance matrices of the gut microbiome (283 taxa at species level and 220 at genus level) to classify between patients with inflammatory bowel disease (IBD) and healthy control (1,569 samples). We found that applying an already published data transformation before RFE improves feature stability significantly. Moreover, we performed an in-depth evaluation of different variants of the data transformation and identify those that demonstrate better improvement in stability while not sacrificing classification performance. To ensure a robust comparison, we evaluated stability using various similarity metrics, distances, the common number of features, and the ability to filter out noise features. We were able to confirm that the mapping by the Bray–Curtis similarity matrix before RFE consistently improves the stability while maintaining good performance. Multilayer perceptron algorithm exhibited the highest performance among 8 different machine learning algorithms when a large number of features (a few hundred) were considered based on the best performance across 100 bootstrapped internal test sets. Conversely, when utilizing only a limited number of biomarkers as a trade-off between optimal performance and method generalizability, the random forest algorithm demonstrated the best performance. Using the optimal pipeline we developed, we identified 14 biomarkers for IBD at the species level and analyzed their roles using Shapley additive explanations.

**Conclusion:**

Taken together, our work not only showed how to improve biomarker discovery in the metataxonomic field without sacrificing classification performance but also provided useful insights for future comparative studies.

## Introduction

Next-generation sequencing technologies allow reconstructing the internal composition of the whole microbial community (microbiota) present in a sample possibly exploiting 2 different approaches: whole-genome shotgun sequencing (WGS) and targeted amplicon sequencing of 16S ribosomal RNA (16S rDNA-seq) [1, 2]. The first focuses on all genomes, while the second only on a region of a single gene (i.e., 16S rRNA gene). Different bioinformatics preprocessing pipelines can be used to obtain the so-called abundance matrix and taxonomy matrix [3, 4] from raw-read data. The abundance matrix describes the relative abundance of different operating taxonomic units (OTUs) or of different amplicon sequence variant (ASVs) [5, 6] on each sample, while the taxonomy matrix contains information about the taxonomy of each OTU or ASV (i.e., kingdom, phylum, class, order, family, genus, species).

Machine learning (ML) can be used to classify samples based on their taxa composition, thus identifying a microbial signature that characterizes host phenotypes. Recently, several studies exploited different ML-based techniques to develop models for predicting disease states, such as inflammatory bowel disease (IBD) [7, 8], colorectal cancer [9, 10], and cardiovascular disease [11], using microbial data. This growing interest is mainly due to the potential impact on diagnosis and therapeutic target identification.

However, the application of ML methods to microbiota might be challenging for a number of reasons [12]. Despite the existence of several consortium studies [13–16], there is a lack of standards in data structures, metadata collection, and preprocessing. This leads to limited generalizability [17, 18] of the results in terms of diagnostic and prognostic biomarkers—that is, taxa that could be used as diagnostic or prognostic markers [19] and that, as such, need to be robust and reproducible indicators of the biological state.

**Figure 1:**
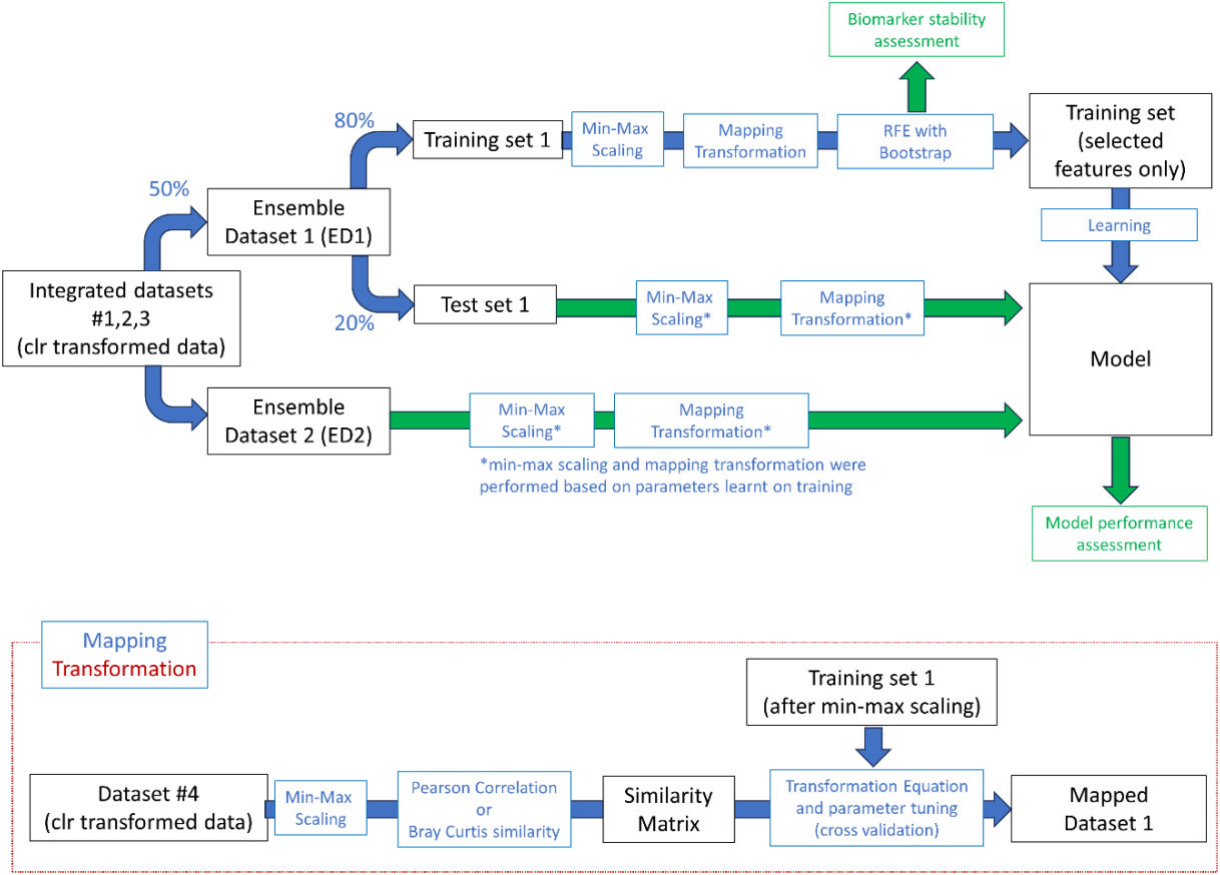
Diagram for the overall experiments. The figure reports the analysis performed on ensemble dataset 1 using ensemble dataset 2 only for testing (black: inputs and outputs; blue: analysis steps; green: results assessment). A symmetric analysis was performed on ensemble dataset 2 using ensemble dataset 1 for testing. The inset represents the details of the mapping transformation procedure.

In this work, we performed a comprehensive evaluation of biomarker stability and classification approaches in the context of gut metataxonomic data used to identify a microbial signature of IBD-affected patients versus healthy controls. Fig. 1 summarizes the whole analysis pipeline. Three different datasets (described in *Methods, Data*) were merged to increase the number of examples. The final merged dataset consists of 1,569 samples in total, where 702 samples were identified as IBD-affected patients and the others as healthy controls. The merged dataset was split in two, thus obtaining ensemble dataset 1 (ED1) and ensemble dataset 2 (ED2) by mixing the samples from the original studies. Note that, although the percentage of samples from different datasets is similar in ED1 versus ED2, we expected the abundance of each taxon to be different in the 2 datasets due to the high number of features and the high variability and sparsity of metagenomics datasets [20]. This characteristic might potentially affect the performance and generalizability of methods, which is a property we wanted to check in our experiments.

Each dataset was given as input to a “typical” ML pipeline, including splitting the data into test and training sets, feature selection, and a final classification step. Since generalized biomarker discovery is a key point in this landscape, our effort focused on studying the feature selection step. In particular, we used recursive feature elimination (RFE) within a bootstrap embedding [21] as described in *Methods, Feature selection approach* to identify and select robust features. Moreover, the integration of prior knowledge in the learning process was investigated as a method to achieve higher stability of the biomarker list [22]. A fourth external dataset for which the samples’ labels were not available was used for this purpose, to compute feature similarity. We refer to this procedure as mapping strategy, since it is based on a kernel-based data transformation that projects data in a new space where the more similar 2 features are, the closer they are mapped in the new space. The basic idea is to take into account the correlation of different taxa: if some features are strongly correlated, then they likely have similar importance and are equally relevant for the classification task. More details about the mapping strategy are provided in *Methods, Mapping*. In *Methods, Evaluation metrics*, we introduce all metrics used to assess features stability (robustness of taxa selected).

It is worth noting that there are similar approaches that use a similarity matrix to map similar features into closer space [23–26]. Among others, AggMapNet utilizes a unique approach for data transformation, converting the original data into multichannel 2-dimensional (2D) spatial-correlated images through pairwise correlation distances. This is achieved by employing the manifold learning method called Uniform Manifold Approximation and Projection (UMAP) [27]. Through a preliminary clustering step, various channels are selected based on the pairwise correlation distances among features. Subsequently, the feature maps are fed as input to machine learning models, such as convolutional neural networks, enabling effective classification tasks. However, traditional ML methods require as input 1-dimensional (1D) vectors. Our approach can map unordered features as 1D vectors for conventional ML models and is therefore somehow complementary to the AggMapNet approach.

With the selected set of features, we used different classification approaches to classify IBD-affected patients and healthy controls—namely, logistic regression, support vector machines, random forests, extreme gradient boosting, and neural networks. In *Methods, Classification model algorithms*, we introduce all the prediction methods used in our study. To assess the generalizability of the classifiers and the robustness of the microbial signature, the models developed using ED1 were tested on test 1 (obtained by splitting ED1 in training and test in proportions 80% and 20%, respectively) and on the entire ED2. Similarly, models developed using ED2 were tested on test 2 and on the entire ED1.

Using the Bray–Curtis similarity matrix to map the data provides the best, improved stability, without sacrificing the classification performance. Using this pipeline, we selected the top 14 features as a trade-off between optimal performance and method generalizability. The best-performing algorithm on this robust set of features was the random forest. We further investigated the role of these biomarkers using Shapley values [28–30].

To ensure the reproducibility of the results, datasets and all code written in support of this publication are publicly available in the *GigaScience* Database [31].

## Methods

### Data

Four datasets were downloaded from Qiita, an open-source microbial study management platform (see Table 1 for the reference Qiita study ID for reproducibility) [16, 32–34]. Table 1 summarizes the characteristics of the different datasets, such as 16S region sequenced, the specimen analyzed, the patient’s geographic origin, and the abundance matrices’ dimensionality. Throughout the article, we will refer to each dataset with the ID indicated in the first column (Dataset#).

**Table 1:** Summary of datasets used in the study. The table shows, in different columns, the ID of the dataset (Dataset#), the Qiita study ID, the sequenced hypervariable region (16S region), the specimen analyzed (Product), the patient’s geographic origin (Geographic localization), and the number of samples labeled as IBD versus non-IBD. CD stands for Crohn’s disease and UC for ulcerative colitis.

Dataset Information							
			Biological specimen	Geographic localization	Selected samples for the study		
Dataset#	Qiita study ID	16S region			Total	IBD	Non-IBD
Dataset 1 [16]	11,484	V4	Feces	USA	96	95 CD:75/UC:20	1
Dataset 3 [32]	2151	V4	Feces	USA	836	32 CD:10/UC:22	804
Dataset 2 [33]	1629	V4	Feces	Sweden	637	575 CD:251/UC:324	62
Dataset 4 [34]	10,317	V4	Feces	USA, UK, AU	444	N/A	N/A
**Ensemble Dataset Information (Dataset 1 + Dataset 2 + Dataset 3)**							
Ensemble Dataset 1—Training Dataset					627	281	346
Ensemble Dataset 2—Training Dataset					628	281	347
Ensemble Dataset 1—Test Dataset					157	70	87
Ensemble Dataset 2—Test Dataset					157	70	87

We downloaded the metadata file, with all the available covariates (e.g., sample ID, age, sex, weight), the *.biom* file with the abundance matrices of the processed 16S rDNA sequences, and the associated taxonomy. For all the datasets, the abundance matrices were obtained by applying the same bioinformatics preprocessing steps—that is, trimming (QIIMEq2 1.9.1) and Pick closed-reference OTUs (QIIMEq2 1.9.1) [35] as implemented in Qiita.

Several preprocessing tools have been developed to handle with 16S sequencing data characteristics, such as log ratio–based transformation, normalization, and zero imputation [36–38]. However, no standard approach has been identified yet. We preprocessed each dataset independently and then combined them in ED1 and ED2. First, all the taxa with the same taxonomy classification (i.e., same species level or genus level) were aggregated and their respective counts were summed, as usually done in microbiome studies [19, 3, 39]. We considered both species and genus levels independently, thus obtaining 2 abundance matrices for each dataset. After that, we filtered out those taxa with more than 99% of abundances equal to 0. It is worth noting that usually, in the field of microbiota analysis, a threshold of 95% or 90% is used. However, lower thresholds can alter the composition of the abundance profiles (i.e., the abundances of all the features in a sample) and have an effect on the following normalization step [40]. Then we divided the abundance profiles by the geometric mean and took the log (base 2) of the ratios leveraging on the idea of clr transformation, using a pseudocount value equal to the minimum observed data.

After finishing preprocessing Dataset 1 + Dataset 2 + Dataset 3 separately, we integrated the 3 datasets as shown in Fig. 1. To this purpose, from the entire set of features, only the ones common throughout all datasets were kept. In this way, we selected a core set of features with 283 taxa at the species level and 220 at the genus level. After dataset splitting in ED1 and ED2, min–max scaling was performed to scale features into the same range. The preprocessing was performed separately to the external dataset (Dataset 4) following the same steps described above.

### Feature selection approach

For each taxonomic level (genus, species) and each dataset ED1 and ED2, RFE was used as a feature ranking method to obtain the optimal number of features [21] as described in the following. RFE was performed 100 times with bootstrapping. In each bootstrap, the training dataset was split into an internal training set and an internal test set. A prediction model was trained within the internal training set using a linear support vector machine (SVM). RFE is a feature selection method that fits a model and removes the weakest feature (or features) until the specified number of features is reached. Linear SVMs were chosen because of their cost-efficiency trade-off and their ability to deal with a high number of predictors [41–43]. The regularization parameter was tuned within each internal training set using grid search and 5-fold cross-validation with the Matthew correlation coefficient (MCC) [44] as the performance index. Feature importance was measured based on the feature weight since data had been standardized in input. The least important feature was eliminated from the dataset iterating the process until only 1 feature was left. Sorting the features from the last eliminated, which will therefore have rank 1, and averaging the ranked lists across the 100 bootstraps, the global feature rank was calculated. Finally, MCC was computed independently on each bootstrap internal test sets for different numbers of features and then averaged across the 100 bootstraps. The number of features corresponding to the maximum MCC (MCC shows either a peak or a saturation effect) was taken as optimum.

RFE was performed (i) without any transformation, (ii) with mapping performed using Pearson correlation, and (iii) with mapping performed using Bray–Curtis similarity, as explained in the following paragraph.

### Mapping

#### Similarity matrix

The similarity matrix is a symmetric matrix encoding the knowledge about the likeness between features. In this article, we used either Pearson correlation [45] or Bray–Curtis similarity [46]. Pearson correlation is the linear correlation between 2 sets, dividing the covariance of 2 features by the product of each standard deviation. Bray–Curtis similarity is the comparison of the composition between 2 different sites, dividing twice the sum of the lesser value for only those features in common between 2 sites by the total sum of values counted at both sites for all the features. Assume ${x}_{ij}$ as ${j}^{th}( < n)$ feature value of ${i}^{th}( < m)\ $data sample. Then we can define the list of ${a}^{th}\ $and ${b}^{th}$ feature value as $A = [ {{x}_{1a},{x}_{2a}, \ldots \ {x}_{ma}} ]$ and $B = [ {{x}_{1b},{x}_{2b}, \ldots \ {x}_{mb}} ]$. Pearson correlation and Bray–Curtis similarity are defined as follows:


(1) \begin{eqnarray*}
Pearson{\mathrm{\ }}Correlation = {\mathrm{\ }}\frac{{cov\left( {A,B} \right)}}{{{\sigma }_A{\sigma }_B}}
\end{eqnarray*}

(2) \begin{eqnarray*}
Bray{\mathrm{\ }}Curtis{\mathrm{\ }}Similarity = \frac{{2\mathop \sum \nolimits_1^m min\left( {A\left[ i \right],B\left[ i \right]} \right)}}{{\left( {\sum A} \right) + \left( {\sum B} \right)}}
\end{eqnarray*}

It should be noted that, to avoid introducing any bias, the similarity matrices were calculated on dataset 4 (see Fig. 1 and Table 1), an external dataset used only to calculate the similarity matrix to perform the mapping transformation (see *Methods, Mapping, Similarity Matrix*).

#### Mapping transformation and its theoretical advantages

Feature mapping is a crucial steps in machine learning that can significantly impact model performance. Feature mapping involves transforming raw input data into a format suitable for the learning algorithm, enabling the extraction of meaningful patterns and relationships. By converting complex and diverse features into a more structured representation, feature mapping empowers the model to discern relevant information, leading to more accurate predictions. In the context of omics data, due to the abundance of features, the problem of identifying relevant features for the predictive model becomes underconstrained, leading to numerous potential sets of relevant features that could achieve comparable accuracy. To address this, we leveraged supplementary data from an external dataset (dataset 4) to impose additional constraints during feature mapping. In essence, this approach aims to account for strong correlations among certain features, indicating their similar importance for the classification task. As a result, we ensure that these correlated features are equally relevant, enhancing the overall performance of the model.

Information about feature correlation is integrated by mapping data using a kernel transformation that has been shown to possibly alleviate feature instability [22]. Transformation matrix ${\mathrm{P}}$ is obtained using the equation ${\mathrm{P}} = {{\mathrm{D}}}^{ - 1}( {{\mathrm{I}} + {\mathrm{\alpha }}( {{\mathrm{S}} - {\mathrm{I}}} )} )$, where ${\mathrm{S}}$ is the similarity matrix, ${\mathrm{D}}$ is the diagonal matrix whose elements are the sum of the elements in the rows of the matrix ${\mathrm{I}} + {\mathrm{\alpha }}( {{\mathrm{S}} - {\mathrm{I}}} )$, and ${\mathrm{\alpha }}$ is a tuning parameter. The value of ${\mathrm{\alpha }}$ was decided for each experiment using 5-fold cross-validation within its internal training dataset using a grid of 0.01 and from 0.05 to 1 by step 0.05. In our approach, mapping was used only in the RFE step.

### Evaluation metrics

#### Stability

Different rank-based stability indexes were used to evaluate the robustness of the feature selection algorithm: Spearman’s rank correlation coefficient (SRCC), Hamming distance, Pearson correlation, and Bray–Curtis dissimilarity [47, 48].

Since the ranks are distinct integers, ***SRCC*** between 2 rank sets can be calculated as follows:


(3) \begin{eqnarray*}
SRCC = 1 - 6{\mathrm{*}}\mathop \sum \limits_{each{\mathrm{\ }}feature} \frac{{{d}^2}}{{{n}^3 - n}}
\end{eqnarray*}

where *d* is the difference between the 2 ranks of each feature and *n* is the total number of ranked features.

***Hamming distance*** between 2 rank sets is calculated as the proportion of disagreeing components as follows:


(4) \begin{eqnarray*}
Hamming{\mathrm{\ }}Distance = \frac{1}{n}\mathop \sum \limits_{each{\mathrm{\ }}feature} \left\{ {\begin{array}{@{}*{1}{c}@{}} {1,\ if\ ranks\ are\ not\ the\ same}\\ {0,\ otherwise} \end{array}} \right. \end{eqnarray*}

The equation of ***Pearson correlation and Bray–Curtis dissimilarity*** is identical to Eq. (1) and Eq. (2), but *A* and *B* now comprise rank of each feature at each bootstrap where $\ {x}_{ij}\ $ represent the rank of ${i}^{th}( {i = 1,\ \ldots ,\# of\ features} )$ feature of ${j}^{th}( {j = 1,\ \ldots ,\ 100} )$ bootstrap. Bray–Curtis dissimilarity is calculated by subtracting Bray–Curtis similarity value from 1.

***Euclidean distance*** is the length of the line segment connecting 2 different points. The stability indexes described above were computed for each pair of bootstrap samples used for RFE and finally averaged across the 100 * (100 – 1)/2 values.

The ***number of commonly ranked features*** was defined by counting the number of common features across all bootstraps considering a range that starts from the top-ranked single feature and expanding the range one by one. The condition can be varied by defining “common” as consistent across all 100 bootstraps or across at least 66 or 50 bootstraps.

To compare the ability to distinguish unimportant features from important features, a noise-filtering experiment was performed. We added 100 randomly generated noisy features (range 0 to 1 from a uniform distribution) to the original features (range 0 to 1 by min–max scaler) before mapping and after mapping and RFE compared the average rank of noisy features from the average rank of true features (Table 3). With a good noise-filtering algorithm, noise features are expected to have big feature ranks.

#### Performance measurement

MCC was used for the step of recursive feature elimination and to compare the performance of the different models. MCC estimates the correlation between the predictive value and ground-truth value by using the following equation:


(5) \begin{eqnarray*}
MCC = \frac{{TN{\mathrm{*}}TP - FN{\mathrm{*}}FP}}{{\sqrt {\left( {TP + FP} \right)\left( {TP + FN} \right)\left( {TN + FP} \right)\left( {TN + FN} \right)} }}
\end{eqnarray*}

where TP is the number of true-positive, TN of true-negative, FN of false-negative, and FP of false-positive values. Chicco et al. [44] showed the appropriateness of MCC for evaluating binary classification performance by considering all 4 confusion matrix categories.

AUC, accuracy, specificity, sensitivity, positive predictive value (PPV), and negative predictive value (NPV) were also measured to compare models’ performance. AUC stands for area under the ROC curve, and the ROC curve is drawn by placing the false-positive rate, $\ \frac{{FP}}{{FN + TP}}$, to the x-axis and the true-positive rate, $\frac{{TP}}{{TP + FN}}\ $, to the y-axis. Accuracy stands for $\frac{{TP + TN}}{{TP + FP + FN + TN}}$, specificity for $\frac{{TN}}{{TN + FP}}$, sensitivity for $\frac{{TP}}{{TP + FN}}$, PPV for $\frac{{TP}}{{TP + FP}}$, and NPV for $\frac{{TN}}{{TN + FN}}$. Apart from linear SVM, which outputs the class, predictive values should be converted into binary values by establishing a threshold. Considering the positive to negative ratio was not high (with a value of 0.81 as indicated in Table 1), we set the threshold to 0.5.

### Classification model algorithms

Eight different types of prediction algorithms—namely, logistic regression, linear SVM, random forest, XGBoost, perceptron, and multilayer perceptron (MLP) with 1, 2, or 3 hidden layers—were used to classify samples in IBD versus healthy using the features selected within the RFE phase. Within each training dataset, a classification model that predicts the status of IBD was built. Hyperparameters in each model were tuned using 5-fold cross-validation within the training set with grid search.

***Logistic regression*** outputs prediction scores between 0 and 1 by applying logistic function to linear regression. In this study, regularized logistic regression, using L2 penalty (i.e., ridge regression) was used. For the optimization, lbfgs solver was used with the tolerance parameter of 1e-4 (which followed the default setting of the library sklearn.linear_model.LogisticRegression by scikit-learn) [49]. Regularization parameter was tuned using a grid of 2 to the power of (−5, −3, −1, 1, 3, 5).

***Linear SVM*** linearly separates data maximizing the margin between 2 classes. The squared L2 penalty was applied to prevent overfitting. The “rfb” kernel was used, and “gamma” was set as “scale” so that it used 1/(# of features * variation of features) as the value of gamma (which followed the default setting of the library sklearn.svm.LinearSVC by scikit-learn) [49]. Regularization parameter was tuned with a grid of 2 to the power of (−5 to 15 with the step of 2).

***Random forest*** builds many decision trees and uses bagging to make an uncorrelated forest of trees combined for better prediction. Gini impurity was used to decide the splits, while the maximum depth was unlimited so that nodes were expanded until all leaves were pure or contained less than the parameter “min samples split.” The parameter “min samples split,” which is the minimum number of samples for splitting an internal node, and the parameter “min samples leaf,” which is the minimum of samples to be at a leaf node, were set equal to 2. The number of features to search for the best split was set as $\sqrt {\# {\mathrm{\ of\ features}}} $. Finally, the number of trees in the forest was set as 100.

Extreme gradient boosting (***XGBoost***) is a tree ensemble model that utilizes gradient boosting to combine many trees. XGBRegressor was trained with 200 gradient boosted trees. Base learners had 20 maximum tree depth. Subsample ratio of the training instance was 0.2, while subsample ratio of columns when constructing each tree was 0.5. Minimum loss reduction, which is for the partition on a leaf node, was set to 1. Learning rate was tuned with the grid of 0.005, 0.01, 0.05, 0.1, 0.5. Regularization factor, alpha, which is the L1 regularization term, was with the grid of 1e-3, 1e-2, 0.1, 1, 10.

***Perceptron*** is a single-layer neural network predicting an output with combinations of input values, weights, and bias. ***MLP*** is a feedforward neural network with multiple hidden layers. In the experiments, MLP Regressor, which has a single output layer, was built. LBFGS optimizer minimizing the squared error function was used to optimize weight variables, while ReLu function was utilized for the activation function. When the loss score was under 1e-4, the optimization was considered as converged, and the training stopped. Learning rate and L2 regularization term were tuned with the same procedure with XGBoost. MLP with 1, 2, and 3 hidden layers were constructed with hidden layer size corresponding to the number of features.

## Results

### Mapping transformation and recursive feature elimination

**Figure 2:**
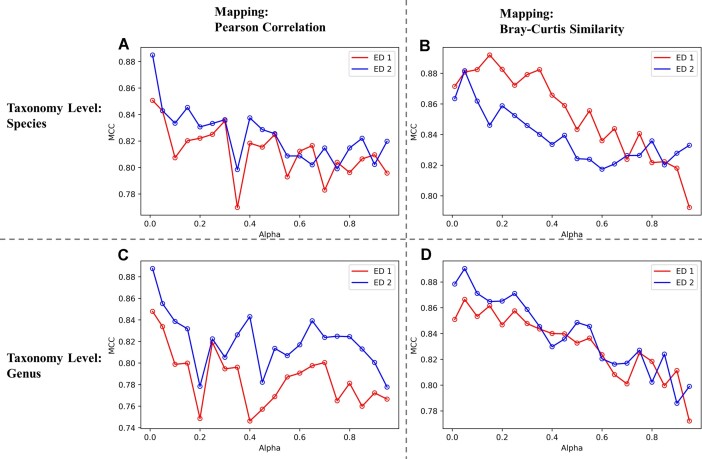
Average MCC across bootstraps at different values of hyperparameter ${\mathrm{\alpha }}$ in mapping transformation. Hyperparameter ${\mathrm{\alpha }}$ is used for controlling the degree of mapping transformation. (A) Species level, mapping by Pearson correlation. (B) Species level, mapping by Pearson correlation. (C) Genus level, mapping by Pearson correlation. (D) Genus level, mapping by Pearson correlation.

Hyperparameter $\alpha $ used in mapping decides how much we weigh the similarity matrix in data transformation when performing RFE and was tuned in cross-validation within each internal test set as explained in *Methods, Mapping, Mapping transformation and its theoretical advantages* (see Fig. 2). Interestingly, the MCC values exhibit different patterns when comparing mapping performed using Pearson correlation and mapping performed using Bray–Curtis similarity. For mapping with Pearson correlation, the MCC shows an unstable profile and a sudden drop as shown in Fig. 2A, C. The optimal α value appears as the smallest value, 0.01, within the grid range of 0.01 to 1. On the other hand, Bray–Curtis similarity mappings demonstrate an initial increase in the performance followed by a stable pattern of decline, with optimal α values equal to 0.15 and 0.05 at the species level for ED1 and ED2, respectively, and equal to 0.05 at the genus level for both ED1 and ED2.

**Figure 3:**
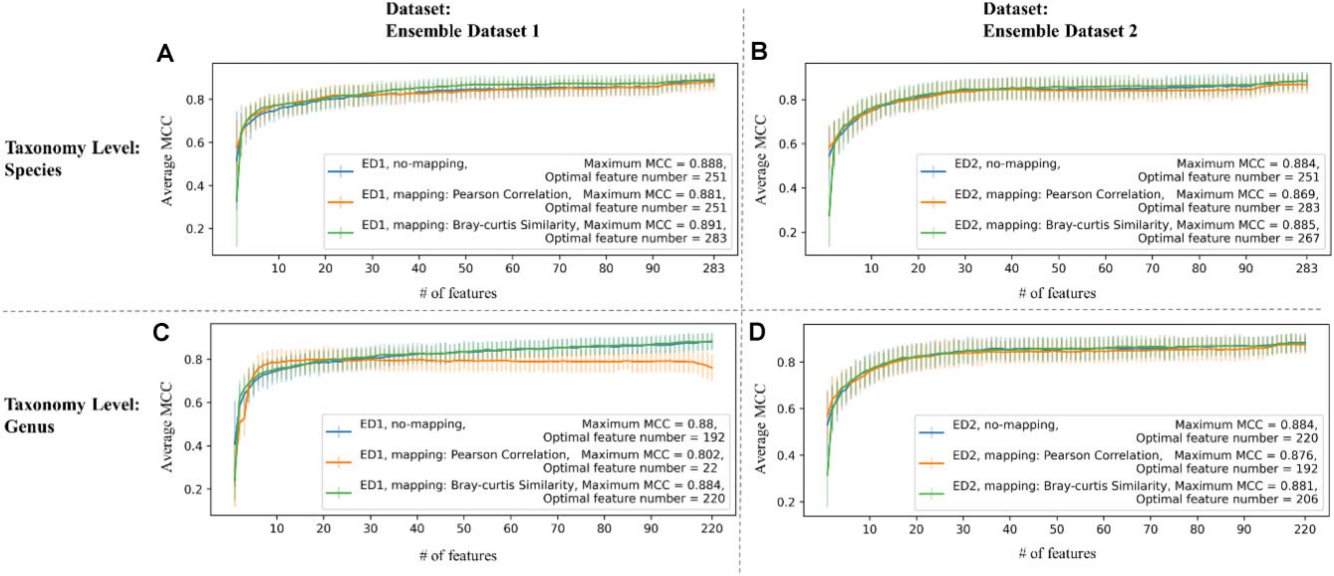
Average MCC across the 100 bootstrapped recursive feature elimination process. MCC (y-axis) at varying number of features (x-axis) averaged across the 100 bootstrap internal test sets. Maximum MCC with optimal feature number is written in each legend. Error bars represent the standard deviation of MCC. (A) Species level, ED1. (B) Species level, ED2. (C) Genus level, ED1. (D) Genus level, ED2.

Performance dependence on the number of features is illustrated in Fig. 3, where MCC is shown (i) with not mapping, (ii) with mapping performed using Pearson correlation, and (iii) with mapping performed using Bray–Curtis similarity. The pattern is similar in the 3 cases, with MCC close to 0.8 slightly increasing with the number of features and saturating only at the very right side of the curve. As a consequence, the optimal number of features is high (87.1% of features are selected on average) in most of the RFE experiments. The only exception is represented by mapping based on Pearson correlation on ED1 at the species level, which reaches the maximum MCC when using 22 features. The maximum value of the MCC (average calculated on the 100 bootstrap samples) is shown in the label of Fig. 3 as “Maximum MCC,” with the corresponding number of features indicated as “Optimal feature number.”

### Feature stability

#### Stability indexes

Table 2 illustrates the values of the stability metrics across the different bootstraps in each experiment. At the species level, mapping data using Bray–Curtis similarity allows reaching better stability with every dataset and every metric. At the genus level, mapping data using Bray–Curtis similarity still shows the better stability in the ED1 dataset (with the exception of Hamming distance and SRCC metrics) and in the ED2 dataset (with the exception of Hamming distance metric).

**Table 2:** Stability metrics for each recursive feature elimination experiment. For each dataset, the best stability value is highlighted in bold.

Level: Species					
	SRCC	Pearson correlation	Hamming distance	Bray–Curtis dissimilarity	Euclidean distance
ED1					
No mapping	0.662	0.653	0.919	0.085	1.402
Mapping: Pearson correlation	0.652	0.642	0.92	0.086	1.425
Mapping: Bray–Curtis similarity	**0.667**	**0.689**	**0.918**	**0.08**	**1.325**
ED2					
No mapping	0.617	0.639	0.925	0.088	1.423
Mapping: Pearson correlation	0.579	0.599	0.928	0.094	1.505
Mapping: Bray–Curtis similarity	**0.643**	**0.662**	**0.921**	**0.084**	**1.355**
**Level: Genus**					
ED1					
No mapping	**0.67**	0.671	0.921	**0.1**	1.689
Mapping: Pearson correlation	0.607	0.618	**0.903**	0.108	1.819
Mapping: Bray–Curtis similarity	0.667	**0.677**	0.919	**0.1**	**1.672**
ED2					
No mapping	0.698	0.715	**0.916**	0.093	1.561
Mapping: Pearson correlation	0.647	0.662	0.922	0.102	1.705
Mapping: Bray–Curtis similarity	**0.707**	**0.733**	0.917	**0.089**	**1.514**

Supplementary Figs. S1 and S2 show the *number of commonly ranked features* across all 100 bootstraps or across at least 66 or 50 bootstraps. At small ranks, the difference between different mapping strategies is not clear as the number of common features across bootstraps is too small. However, as more features are considered, RFE with Bray–Curtis similarity–based mapping shows a greater number of common features in every dataset except ED1 at the genus level.

#### Noise filtering

To assess the noise-filtering ability of the various approaches (*Methods, Evaluation metrics, Stability*), we subtracted the average rank of noisy features from the average rank of original data features (283 features at the species level, 220 features at the genus level). In Table 3, except ED1 at the species level, mapping with Bray–Curtis similarity shows a better noise-filtering score than the others. The superiority of mapping with Bray–Curtis similarity is more apparent at the genus level, as the gap is 3 times bigger compared to the species level (22.87 to 77.66 and 26.06 to 80.49). While mapping with Bray–Curtis similarity shows consistent noise-filtering ability, mapping with Pearson correlation shows unstable noise-filtering ability, which is sometimes worse than no mapping.

**Table 3:** Noise-filtering result for each recursive feature elimination experiment. The margin between the rank of noise features and original features is calculated.

Noise-filtering score	avg(rank of noises)—avg(rank of real) * rank by recursive feature elimination	
Taxonomy level/algorithm	Species	Genus
ED 1: no mapping	11.30	34.61
ED 1: mapping with Pearson correlation	27.17	−5.92
ED 1: mapping with Bray–Curtis similarity	22.87	77.66
ED 2: no mapping	20.19	33.21
ED 2: mapping with Pearson correlation	14.10	33.42
ED 2: mapping with Bray–Curtis similarity	26.06	80.49

### Performance comparison by selected set of features

Prediction models were built based on the optimal set of features identified using linear SVM-based RFE and Bray–Curtis similarity–based mapping (Fig. 3). To validate the methods, the models trained on ED1 were tested in both test 1–ED1 and on the entire ED2, and models trained on ED2 were tested on test 2–ED2 and on the entire ED1. Eight different prediction algorithms (see *Methods, Classification Model Algorithms*) were tested. Overall performance obtained by different methods using different metrics (AUC, accuracy, sensitivity, specificity, PPV, NPV, MCC) is illustrated in Supplementary Tables S1–S4. Supplementary Table S5 summarizes the best MCC with the best-performing algorithm in each experiment. MLP with 1 or 2 hidden layers shows the best MCC in most of the experiments (23 among 24 results).

As the optimal number of features is high in most RFE experiments (Fig. 3), prediction models were also trained and compared with a constant number of a few top features. We choose to consider the top 14 features (see *Methods, Full Data Experiment and Biomarker Selection*). Results obtained using different algorithms are shown in Supplementary Table S6. With a smaller number of selected features, the performance slightly decreases and the best-performing algorithm varies. Random forest shows the best performance in the most cases (15 among 24), followed by MLP-1,2 hidden layer, XGBoost, logistic regression, and linear SVM. RFE without mapping shows a better performance than RFE with mapping by Bray–Curtis similarity with 0.0016 on average. Considering the small gap, mapping with Bray–Curtis similarity, while improving feature stability, does not seem to affect method performance in terms of MCC, which overall, remains quite stable.

### Full data experiment and biomarker selection

The best pipeline for biomarker selection (linear SVM-based RFE with mapping using Bray–Curtis similarity) is applied to the full dataset at the species level without any split (i.e., training set is the combination of training sets in ED1 and ED2, test set of test sets in ED1 and ED2) to obtain the possible biomarkers for IBD. During the RFE process, we compute the average MCC across the 100 bootstrap internal test sets. Similar to Fig. 3, average MCC slightly increases with the number of features, and the maximum MCC is reached corresponding to 267 features among the 283 considered. The 8 different predictive models presented in *Methods, Classification Model Algorithms* are trained using the selected features, illustrated in Supplementary Table S7. MLP-1,2 hidden layers consistently show the highest performance in terms of MCC, confirming what was previously found.

**Figure 4:**
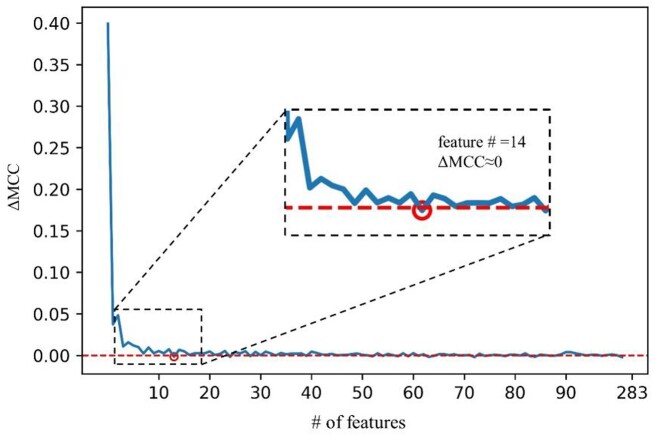
Differential of average MCC during RFE. Average MCC is calculated in each feature number, and each average MCC is subtracted by its former average MCC to calculate differentials. x-axis: the number of features, y-axis: differential of average MCC, horizontal red line: y = 0.

As the optimal number of features is high in most RFE experiments (Fig. 3), prediction models were also trained and compared with a constant number of a few top features. We choose to consider the top 14 features as a tradeoff between optimal performance and generalizability potential, based on results shown in Fig. 4 where we calculated the  ΔMCC as the difference in MCC at different subsequent numbers of features and selected the value at which the  ΔMCC starts to converge to zero. Compared to models with optimal number of features (267), overall performance decreases and the best model algorithm changes. While MLP with 1 hidden layer is the best algorithm with MCC 0.963 in Supplementary Table S7, it decreases to 0.826. Instead, random forest decreases to 0.845 from 0.854, which is the maximum MCC among the 8 algorithms.

**Figure 5:**
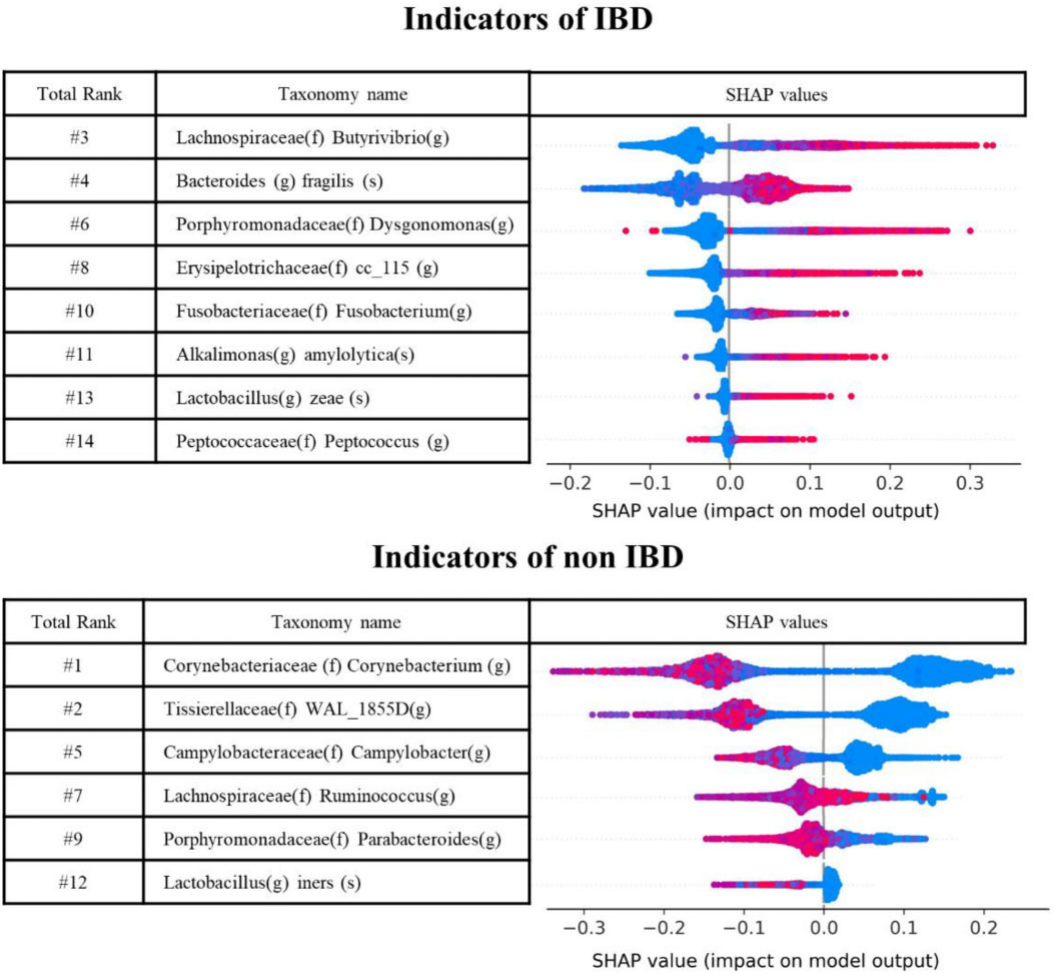
Shapley additive explanation (SHAP) summary plot of primary indicators of IBD and non-IBD. Random forest model trained by top 14 features in RFE (species level, trained by the combination of training sets in ED1 and ED2) is used to calculate SHAP values. SHAP values are measured by the training dataset. Total rank is the rank between 14 features used for training the model.

With the final random forest–based predictive model, Shapley values are calculated on the training, test, and external datasets for the top 14 ranked features. Shapley values calculate the extent that each feature at each data sample contributes to the change of output of the prediction model. Since Shapley values are model agnostic, which provides local explanations, they are calculated on the training and test datasets and on dataset 4 (for which we do not know the sample labels but still can run the models and calculate the Shapley values). Shapley values on the training set are shown in Fig. 5 (Shapley values on the test and the external dataset 4 are shown in Supplementary Table S8). The rank of the 14 features based on Shapley values shows a high similarity across different datasets (training, test, and external dataset 4), and the directions, which increase or decrease the possibility of IBD, are identical. Random forest model with 14 selected features determines *Lachnospiraceae (f) Butyrivibrio (g), Bacteroides (g) fragilis (s), Porphyromonadaceae (f) Dysgonomonas (g), Erysipelotrichaceae (f) cc_115 (g), Fusobacteriaceae (f) Fusobacterium (g), Alkalimonas (g) amylolytica (s), Lactobacillus (g) zeae (s), Peptococcaceae (f) Peptococcus (g)* as indicators of IBD state, and *Corynebacteriaceae (f) Corynebacterium (g), Tissierellaceae (f) WAL_1855D (g), Campylobacteraceae (f) Campylobacter (g), Lachnospiraceae (f) Ruminococcus (g), Porphyromonadaceae (f) Parabacteroides (g), Lactobacillus (g) iners (s)* as indicators of negative of IBD (*f*: family, *g*: genus, *s*: species level).

## Discussion

Stable feature selection is a prerequisite to decide strong biomarkers. To prove the validity of a set of features as biomarkers, they should be observed consistently every time a feature selection algorithm is applied. In this article, we performed RFE multiple times using bootstrapping and checked the level of consistency of feature ranks between trials using stability indexes. Moreover, we investigated various strategies that could improve the stability of selected biomarkers.

First, we used bootstrap to cope with the scarcity of samples in the training set compared to the number of features [50]. In this way, we generated various classifiers, selected various features on various data splits, and then averaged the results, preserving a high ranking only for those features that are consistently the most discriminating features across the splits. Second, since the high number of features makes the problem underconstrained (i.e., many possible sets of features can be considered relevant to the task and equally good in terms of accuracy), we used additional information from an external dataset (dataset 4) to include additional constraints in terms of feature mapping. In other words, if some features are strongly correlated, then they likely have similar importance, and we want them to be equally relevant for the classification task.

Three different datasets were merged to increase the number of examples and mitigate the potential batch effect and then split in two. As the division of the dataset was performed using bootstrapping, the ratio of non-IBD versis IBD was preserved, and the ratio of the data source was also preserved without significant difference, as shown in Supplementary Table S9. However, the distribution of each feature may differ as the size of the dataset was small compared to the number of features.

Mapping transformation with linear SVM-based RFE improved the stability without the loss of performance. We recommended using Bray–Curtis similarity to improve the stability of the selected features since, in most cases, mapping using Bray–Curtis similarity showed a significant increase of stability compared to other approaches and higher noise-filtering score, at both genus and species levels.

Moreover, as shown in Supplementary Figs. S1 and S2, mapping with Bray–Curtis similarity presents a higher number of common features, already at an early stage of RFE.

Notably, the Bray–Curtis similarity was designed for compositional data (a composition is represented by a vector of proportions with respect to a total sum on the sample under observation) and is therefore more robust to spurious correlations introduced by the fact that the taxonomic abundances are multivariate observations whose information content is closely linked to the relationship between the components.

A possible drawback of our method is that the mapping transformation compresses the information by shortening the distance between similar features. In case the degree of the transformation is high ($\alpha \ $ in the transformation matrix equation $P = {D}^{ - 1}( {I + \alpha ( {S - I} )} )$), the loss of potential information can be important. Choosing $\alpha $ in cross-validation as done in this work might help mitigate this risk, although at a higher computational cost. Indeed, in our experiments, the optimal choice of $\alpha $ did not highlight a loss in classification performance.

There are similar approaches that use the similarity matrix to map similar features into closer space [23–26]. AggMapNet maps the original data into multichannel 2D spatial-correlated images based on their pairwise correlation distances using the manifold learning method UMAP [27]. Different channels are chosen based on a preliminary clustering step, which is again based on pairwise correlation distances between features. Features maps are then given as input to machine learning methods such as convolutional neural networks to perform classification. Results obtained using the AggMapNet [23, 24] approach on our dataset are shown in Supplementary Table S10 and compared with our pipeline (Bray–Curtis similarity–based mapping + RFE + MLP). This comparison proves the strength of our pipeline in the situation of small data and a high number of features, which is typical of omics studies. Differently from AggMapNet [23, 24], our pipeline does not project the data in multichannel 2D spatial-correlated images and thus is not suitable for 2D data representation and omics data integration.

To further assess the consistency of our results and the robustness of our approach, we calculated the False Discovery Rate (FDR)-adjusted *P* values using the Wilcoxon rank-sum test, fold change and average, and standard deviation information of the top 14 biomarkers we selected in Supplementary Table S11. We found that 13 of 14 biomarkers had a *q* value below 0.001 when comparing between IBD and non-IBD samples. However, despite the statistically significant differences in the markers, 52.3% of the features showed significant differences between IBD and non-IBD groups, which implies that relying solely on statistical tests may not be sufficient in determining good biomarkers. The robustness of our algorithm and the excellence of machine learning algorithms are shown by high-performing prediction for this sparse type of dataset.

It should be reported that we originally performed the overall experiment without logarithmic transformation (data not shown) and checked later that logarithmic transformation improved the performance significantly. We expect the logarithmic transformation has accounted for the highly skewed distribution of microbial abundances [51]. Before using the logarithmic transformation, the best-performing algorithm was random forest instead of MLP. While tree-based ensembles utilize feature splitting based on a set of thresholds, neural network uses continuous values to build nonlinear relationships among variables, and logarithmic transformation is expected to advantage the performance in this aspect.

However, when fewer features are considered, random forest outperforms other methods, including MLP. In recent studies, random forest provides generally higher performance than other conventional algorithms for microbial data analysis [52–55]. We suppose the higher complexity of the MLP algorithm takes more advantage when a higher number of features is considered, whereas in case fewer features are considered, the “split” approach of tree-based ensemble models may fit to microbial data better.

## Availability of Source Code and Requirements

Project name: MLonMicrobiome

Project homepage: https://gitlab.com/sysbiobig/mlonmicrobiome

Operating system(s): Platform independent

Programming language: Python, R

Other requirements: R 4.1.3, phyloseq 1.27.6, openxlsx 4.2.4, Python 3.85, scikit-learn 1.0.2

License: GNU GPL

RRID: N/A

## Supplementary Material

giad083_GIGA-D-23-00164_Original_Submission

giad083_GIGA-D-23-00164_Revision_1

giad083_GIGA-D-23-00164_Revision_2

giad083_Response_to_Reviewer_Comments_Original_Submission

giad083_Response_to_Reviewer_Comments_Revision_1

giad083_Reviewer_1_Report_Original_Submission Wan Xiang Shen -- 7/10/2023 Reviewed

giad083_Reviewer_2_Report_Original_Submission Jakob Wirbel -- 7/16/2023 Reviewed

giad083_Reviewer_2_Report_Revision_1 Jakob Wirbel -- 8/29/2023 Reviewed

giad083_Supplemental_File

## Data Availability

Four datasets were downloaded from Qiita, an open-source microbial study management platform (see Table 1 for the reference Qiita study ID for reproducibility) [16, 32–34]. Data after each preprocessing stage can be accessed by our code repository. Our code and other data further supporting this work, including DOME-ML annotations, are openly available in the *GigaScience* repository GigaDB [31].

## Abbreviations

AUC: area under the receiver operating characteristic curve; CD: Canberra distance; IBD: inflammatory bowel disease; MCC: Matthew correlation coefficient; MLP: multilayer perceptron; RF: random forest; RFE: recursive feature elimination; SRCC: Spearman’s rank correlation coefficient; SVM: support vector machine; WGS: whole-genome shotgun sequencing; XGBoost: Extreme Gradient Boosting.

## References

[1] Quince C, Walker AW, Simpson JT et al. Shotgun metagenomics, from sampling to analysis. Nat Biotechnol. 2017;35(9):833–44. Erratum in: Nat Biotechnol. 2017;35(12):1211. 10.1038/nbt.3935. 28898207

[2] Kamble A, Sawant S, Singh H. 16S ribosomal RNA gene-based metagenomics: a review. Biomed Res J. 2020;7:5–11. 10.4103/BMRJ.BMRJ_4_20.

[3] Breitwieser FP, Lu J, Salzberg SL. A review of methods and databases for metagenomic classification and assembly. Brief Bioinform. 2019;20(4):1125–36. 10.1093/bib/bbx120. 29028872 PMC6781581

[4] Bharti R, Grimm DG. Current challenges and best-practice protocols for microbiome analysis. Brief Bioinform. 2021;22(1):178–93. 10.1093/bib/bbz155. 31848574 PMC7820839

[5] Blaxter M, Mann J, Chapman T, et al. Defining operational taxonomic units using DNA barcode data. Philos Trans R Soc Lond B Biol Sci. 2005;360(1462):1935–43. 10.1098/rstb.2005.1725. 16214751 PMC1609233

[6] Callahan BJ, McMurdie PJ, Holmes SP. Exact sequence variants should replace operational taxonomic units in marker-gene data analysis. ISME J. 2017;11(12):2639–43. 10.1038/ismej.2017.119. 28731476 PMC5702726

[7] Manandhar I, Alimadadi A, Aryal S, et al. Gut microbiome-based supervised machine learning for clinical diagnosis of inflammatory bowel diseases. Am J Physiol Gastrointest Liver Physiol. 2021;320(3):G328–37. 10.1152/ajpgi.00360.2020. 33439104 PMC8828266

[8] Wang X, Xiao Y, Xu X, et al. Characteristics of fecal microbiota and machine learning strategy for fecal invasive biomarkers in pediatric inflammatory bowel disease. Front Cell Infect Microbiol. 2021;11:711884. 10.3389/fcimb.2021.711884. 34950604 PMC8688824

[9] Thomas AM, Manghi P, Asnicar F et al. Metagenomic analysis of colorectal cancer datasets identifies cross-cohort microbial diagnostic signatures and a link with choline degradation. Nat Med. 2019;25(4):667–78. Erratum in: Nat Med. 2019;25(12):1948. 10.1038/s41591-019-0405-7. 30936548 PMC9533319

[10] Gao Y, Zhu Z, Sun F. Increasing prediction performance of colorectal cancer disease status using random forests classification based on metagenomic shotgun sequencing data. Synth Syst Biotechnol. 2022;7(1):574–85. 10.1016/j.synbio.2022.01.005. 35155839 PMC8801753

[11] Aryal S, Alimadadi A, Manandhar I, et al. Machine learning strategy for gut microbiome-based diagnostic screening of cardiovascular disease. Hypertension. 2020;76(5):1555–62. 10.1161/HYPERTENSIONAHA.120.15885. 32909848 PMC7577586

[12] Marcos-Zambrano LJ, Karaduzovic-Hadziabdic K, Loncar Turukalo T et al. Applications of machine learning in Human microbiome studies: a review on feature selection, biomarker identification, disease prediction and treatment. Front Microbiol. 2021;12:634511. 10.3389/fmicb.2021.634511. 33737920 PMC7962872

[13] Human Microbiome Project Consortium . Structure, function and diversity of the healthy human microbiome. Nature. 2012;486(7402):207–14. 10.1038/nature11234. 22699609 PMC3564958

[14] Thompson LR, Sanders JG, McDonald D et al. Earth Microbiome Project Consortium. A communal catalogue reveals Earth's multiscale microbial diversity. Nature. 2017;551(7681):457–63. 10.1038/nature24621. 29088705 PMC6192678

[15] Integrative HMP (iHMP) Research Network Consortium . The Integrative Human Microbiome Project. Nature. 2019;569(7758):641–8. 10.1038/s41586-019-1238-8. 31142853 PMC6784865

[16] Lloyd-Price J, Arze C, Ananthakrishnan AN et al. Multi-omics of the gut microbial ecosystem in inflammatory bowel diseases. Nature. 2019;569(7758):655–62. 10.1038/s41586-019-1237-9. 31142855 PMC6650278

[17] Hornung BVH, Zwittink RD, Kuijper EJ. Issues and current standards of controls in microbiome research. FEMS Microbiol Ecol. 2019;95(5):fiz045. 10.1093/femsec/fiz045. 30997495 PMC6469980

[18] Cernava T, Rybakova D, Buscot F et al. Metadata harmonization-standards are the key for a better usage of omics data for integrative microbiome analysis. Environ Microbiome. 2022;17(1):33. 10.1186/s40793-022-00425-1. 35751093 PMC9233336

[19] Duvallet C, Gibbons SM, Gurry T et al. Meta-analysis of gut microbiome studies identifies disease-specific and shared responses. Nat Commun. 2017;8(1):1784. 10.1038/s41467-017-01973-8. 29209090 PMC5716994

[20] Gloor GB, Macklaim JM, Pawlowsky-Glahn V et al. Microbiome datasets are compositional: and this is not optional. Front Microbiol. 2017;8:2224. 10.3389/fmicb.2017.02224. 29187837 PMC5695134

[21] Guyon I, Weston J, Barnhill S et al. Gene selection for cancer classification using support vector machines. Machine Learning. 2002;46(1/3):389–422. 10.1023/A:1012487302797.

[22] Sanavia T, Aiolli F, Da San Martino G et al. Improving biomarker list stability by integration of biological knowledge in the learning process. BMC Bioinf. 2012;13(Suppl 4):S22. 10.1186/1471-2105-13-S4-S22. PMC3314566 22536969

[23] Shen WX, Liang SR, Jiang YY, et al. Enhanced metagenomic deep learning for disease prediction and consistent signature recognition by restructured microbiome 2D representations. Patterns. 2023;4(1):100658. 10.1016/j.patter.2022.100658. 36699735 PMC9868677

[24] Shen WX, Liu Y, Chen Y. AggMapNet: enhanced and explainable low-sample omics deep learning with feature-aggregated multi-channel networks. Nucleic Acids Res. 2022;50(8):e45. 10.1093/nar/gkac010. 35100418 PMC9071488

[25] Ma S, Zhang Z. OmicsMapNet: transforming omics data to take advantage of deep convolutional neural network for discovery. 2018. arXiv. https://doi.org/10.48550/arXiv.1804.05283

[26] Bazgir O, Zhang R, Dhruba SR et al. Representation of features as images with neighborhood dependencies for compatibility with convolutional neural networks. Nat Commun. 2020;11:4391. 10.1038/s41467-020-18197-y. 32873806 PMC7463019

[27] McInnes L, Healy J, Melville J. UMAP: Uniform Manifold Approximation and Projection. J Open Source Softw. 2018;3(29):861. 10.21105/joss.00861.

[28] Lundberg SM, Lee S-I. A unified approach to interpreting model predictions. Advances in Neural Information Processing Systems. 2017, 4765–4774.. https://papers.nips.cc/paper_files/paper/2017/hash/8a20a8621978632d76c43dfd28b67767-Abstract.html.

[29] Gou W, Ling CW, He Y, et al. Interpretable machine learning framework reveals robust gut microbiome features associated with type 2 diabetes. Diabetes Care. 2021;44(2):358–66. 10.2337/dc20-1536. 33288652 PMC7818326

[30] Gan RW, Sun D, Tatro AR, et al. Replicating prediction algorithms for hospitalization and corticosteroid use in patients with inflammatory bowel disease. PLoS One. 2021;16(9):e0257520. 10.1371/journal.pone.0257520. 34543353 PMC8452029

[31] Lee Y, Cappellato M, Camillo BD. Supporting data for “Machine Learning–Based Feature Selection to Search Stable Microbial Biomarkers: Application to Inflammatory Bowel Disease.” GigaScience Database. 2023. 10.5524/102450. PMC10600917 37882604

[32] Flores GE, Caporaso JG, Henley JB et al. Temporal variability is a personalized feature of the human microbiome. Genome Biol. 2014;15(12):531. 10.1186/s13059-014-0531-y. 25517225 PMC4252997

[33] Halfvarson J, Brislawn CJ, Lamendella R et al. Dynamics of the human gut microbiome in inflammatory bowel disease. Nat Microbiol. 2017;2:17004. 10.1038/nmicrobiol.2017.4. 28191884 PMC5319707

[34] McDonald D, Hyde E, Debelius JW, et al. American gut: an open platform for citizen science microbiome research. mSystems. 2018;3(3):e00031–18. 10.1128/mSystems.00031-18. 29795809 PMC5954204

[35] Caporaso JG, Kuczynski J, Stombaugh J et al. QIIME allows analysis of high-throughput community sequencing data. Nat Methods. 2010;7(5):335–6. 10.1038/nmeth.f.303. 20383131 PMC3156573

[36] Lin H, Peddada SD. Analysis of microbial compositions: a review of normalization and differential abundance analysis. NPJ Biofilms Microbiomes. 2020;6(1):60. 10.1038/s41522-020-00160-w. 33268781 PMC7710733

[37] Lloréns-Rico V, Vieira-Silva S, Gonçalves PJ et al. Benchmarking microbiome transformations favors experimental quantitative approaches to address compositionality and sampling depth biases. Nat Commun. 2021;12(1):3562. 10.1038/s41467-021-23821-6. 34117246 PMC8196019

[38] Baruzzo G, Patuzzi I, Di Camillo B. Beware to ignore the rare: how imputing zero-values can improve the quality of 16S rRNA gene studies results. BMC Bioinf. 2022;22(Suppl 15):618. 10.1186/s12859-022-04587-0. PMC8822630 35130833

[39] Kubinski R, Djamen-Kepaou JY, Zhanabaev T et al. Benchmark of data processing methods and machine learning models for gut microbiome-based diagnosis of inflammatory bowel disease. Front Genet. 2022;13:784397. 10.3389/fgene.2022.784397. 35251123 PMC8895431

[40] Lahti L, Shetty S, Ernst FM et al. Orchestrating Microbiome Analysis with Bioconductor [Beta Version]. 2021, https://github.com/microbiome/OMA.

[41] Sanz H, Valim C, Vegas E, et al. SVM-RFE: selection and visualization of the most relevant features through non-linear kernels. BMC Bioinf. 2018;19:432. 10.1186/s12859-018-2451-4. PMC6245920 30453885

[42] Lin X, Li C, Zhang Y et al. Selecting feature subsets based on SVM-RFE and the overlapping ratio with applications in bioinformatics. Molecules. 2017;23(1):52. 10.3390/molecules23010052. 29278382 PMC5943966

[43] Racedo S, Portnoy I, Vélez J,I et al. A new pipeline for structural characterization and classification of RNA-seq microbiome data. BioData Min. 2021;14(1):31. 10.1186/s13040-021-00266-7. 34243809 PMC8268467

[44] Chicco D, Jurman G. The advantages of the Matthews correlation coefficient (MCC) over F1 score and accuracy in binary classification evaluation. BMC Genomics. 2020;21:6. 10.1186/s12864-019-6413-7. 31898477 PMC6941312

[45] Freedman D, Pisani R, Purves R. Statistics (International Student Edition). 4th ed. New York: WW Norton & Company; 2007. ISBN: 978-0-393-92972-0.

[46] Bray JR, Curtis JT. An ordination of upland forest communities of southern Wisconsin. Ecological Monographs. 1957;27:325–49. 10.2307/1942268.

[47] Mohana CP, Perumal K. A survey on feature selection stability measures. Int J Comput Sci Info Technol. 2016;5(1):ISSN: 2279–0764.. https://www.ijcit.com/archives/volume5/issue1/Paper050114.pdf.

[48] Khaire UM, Dhanalakshmi R. Stability of feature selection algorithm: a review. J King Saud Univs. 2022;34(4):1060–73. 10.1016/j.jksuci.2019.06.012.

[49] Pedregosa F, Varoquaux G, Gramfort A, et al. Scikit-learn: machine learning in Python. JMLR. 2011;12:2825–30.. https://www.jmlr.org/papers/volume12/pedregosa11a/pedregosa11a.pdf.

[50] Di Camillo B, Sanavia T, Martini M. Effect of size and heterogeneity of samples on biomarker discovery: synthetic and real data assessment. PLoS One. 2012;7(3):e32200. 10.1371/journal.pone.0032200. 22403633 PMC3293892

[51] West RM . Best practice in statistics: the use of log transformation. Ann Clin Biochem. 2022;59(3):162–5. 10.1177/00045632211050531. 34666549 PMC9036143

[52] Pasolli E, Truong DT, Malik F et al. Machine learning meta-analysis of large metagenomic datasets: tools and biological insights. PLoS Comput Biol. 2016;12(7):e1004977. 10.1371/journal.pcbi.1004977. 27400279 PMC4939962

[53] Giliberti R, Cavaliere S, Mauriello IE, et al. Host phenotype classification from human microbiome data is mainly driven by the presence of microbial taxa. PLoS Comput Biol. 2022;18(4):e1010066. 10.1371/journal.pcbi.1010066. 35446845 PMC9064115

[54] Bakir-Gungor B, Hacılar H, Jabeer A, et al. Inflammatory bowel disease biomarkers of human gut microbiota selected via different feature selection methods. PeerJ. 2022;10:e13205. 10.7717/peerj.13205. 35497193 PMC9048649

[55] Liñares-Blanco J, Fernandez-Lozano C, Seoane JA et al. Machine learning based microbiome signature to predict inflammatory bowel disease subtypes. Front Microbiol. 2022;13:872671. 10.3389/fmicb.2022.872671. 35663898 PMC9157387

