# Supplementary material for: Machine learning–based feature selection to search stable microbial biomarkers: application to inflammatory bowel disease
Source: Gigascience. 2023 Oct 26;12:giad083. doi: 10.1093/gigascience/giad083 (PMC10600917; doi:10.1093/gigascience/giad083)
Supplement: giad083_GIGA-D-23-00164_Revision_2 [file giad083_giga-d-23-00164_revision_2.pdf]

GigaScience

Machine learning based feature selection to search stable microbial biomarkers: application to inflammatory bowel disease

--Manuscript Draft--

**Manuscript Number:**	GIGA-D-23-00164R2
**Full Title:**	Machine learning based feature selection to search stable microbial biomarkers: application to inflammatory bowel disease
**Article Type:**	Research
**Funding Information:**	Korea Health Industry Development Institute (KHIDI), funded by the Ministry of Health & Welfare (Project Number : HI21C1092) — Mr Youngro Lee<br>Department of Information Engineering of the University of Padova (Grants nr. DI_C_BIRD2020_01) — Mr Marco Cappellato, Prof Barbara Di Camillo
**Abstract:**	Background<br>Biomarker discovery exploiting feature importance of machine learning has risen recently in the microbiome landscape with its high predictive performance in several disease states. To have a concrete selection among a high number of features, Recursive Feature Elimination (RFE) has been widely used in the bioinformatics field. However, machine learning based RFE has factors that decrease the stability of feature selection. In this paper, we suggested methods to improve stability while sustaining performance.<br>Results<br>We exploited the abundance matrices of the gut microbiome (283 taxa at species level and 220 at genus level) to classify between patients with inflammatory bowel disease (IBD) and healthy control (1569 samples). We found that applying an already published data transformation before RFE improves feature stability significantly. Moreover, we performed an in-depth evaluation of different variants of the data transformation and identify those that demonstrate better improvement in stability while not sacrificing classification performance. To ensure a robust comparison, we evaluated stability using various similarity metrics, distances, the common number of features, and the ability to filter out noise features. We were able to confirm that the mapping by the Bray-Curtis similarity matrix before RFE consistently improves the stability while maintaining good performance. Multi-Layer Perceptron (MLP) algorithm exhibited the highest performance among eight different machine learning algorithms when a large number of features (a few hundred) were considered based on the best performance across 100 bootstrapped internal test sets. Conversely, when utilizing only a limited number of biomarkers as a tradeoff between optimal performance and method generalizability, the random forest algorithm demonstrated the best performance. Using the optimal pipeline we developed, we identified fourteen biomarkers for IBD at the species level and analyzed their roles using SHapley Additive exPlanations.<br>Conclusion<br>Taken together our work showed not only how to improve biomarker discovery in the metataxonomic field without sacrificing classification performance, but also provided useful insights for future comparative studies.
**Corresponding Author:**	Barbara Di Camillo<br>University of Padova School of Engineering: Universita degli Studi di Padova Scuola di Ingegneria<br>Padova, ITALY
**Corresponding Author Secondary Information:**	
**Corresponding Author's Institution:**	University of Padova School of Engineering: Universita degli Studi di Padova Scuola di Ingegneria
**Corresponding Author's Secondary Institution:**	
**First Author:**	Youngro Lee

Powered by Editorial Manager® and ProduXion Manager® from Aries Systems Corporation

**First Author Secondary Information:**	
**Order of Authors:**	Youngro Lee
	Marco Cappellato
	Barbara Di Camillo
**Order of Authors Secondary Information:**	
**Response to Reviewers:**	Dear Editor,<br><br>We sincerely appreciate the time and effort you have invested in evaluating our manuscript.<br><br>We have made the revisions mentioned in your letter and manuscript file, which include:<br><br>- Moving the URL to the reference list.<br>- Revising the 'Availability of Source Code and Requirements' section while including the RRID number.<br>- Adding a 'Data Availability' section and citing the GigaDB DOI reference.<br><br>If there is anything we have missed, please do let us know.<br><br>We sincerely hope that our revised manuscript now meets the high standards of GigaScience and is suitable for publication.<br><br>Thank you for your time and consideration.<br><br>Sincerely,<br>Barbara Di Camillo<br><br>Full Prof. in Computer Science<br>Information Engineering Department<br>Via Gradenigo 6B<br>35131, Padova, Italy<br>Mail: barbara.dicamillo@unipd.it<br>Phone: +39 049 827 7671
**Additional Information:**	
**Question**	**Response**
Are you submitting this manuscript to a special series or article collection?	No
**Experimental design and statistics**<br><br>Full details of the experimental design and statistical methods used should be given in the Methods section, as detailed in our Minimum Standards Reporting Checklist. Information essential to interpreting the data presented should be made available in the figure legends.<br><br>Have you included all the information requested in your manuscript?	Yes
**Resources**	Yes

A description of all resources used, including antibodies, cell lines, animals and software tools, with enough information to allow them to be uniquely identified, should be included in the Methods section. Authors are strongly encouraged to cite Research Resource Identifiers (RRIDs) for antibodies, model organisms and tools, where possible.<br><br>Have you included the information requested as detailed in our Minimum Standards Reporting Checklist?	
**Availability of data and materials**<br><br>All datasets and code on which the conclusions of the paper rely must be either included in your submission or deposited in publicly available repositories (where available and ethically appropriate), referencing such data using a unique identifier in the references and in the "Availability of Data and Materials" section of your manuscript.<br><br>Have you have met the above requirement as detailed in our Minimum Standards Reporting Checklist?	Yes

Powered by Editorial Manager® and ProduXion Manager® from Aries Systems Corporation

Click here to access/download;Manuscript;Lee_Cappellato_DiCamillo_Main

Machine learning based feature selection to search stable microbial biomarkers: application to inflammatory bowel disease

Youngro Lee[1,2], Marco Cappellato[3], Barbara Di Camillo[3]*

[1] Department of Electrical and Computer Engineering, Seoul National University, Seoul, Korea

[2] Institute of Engineering Research at Seoul National University, Seoul, Korea

[3] Department of Information Engineering, University of Padova, Padova, Italy

* Corresponding author

E-mail: barbara.dicamillo@unipd.it (BDC)

Abstract

Background

Biomarker discovery exploiting feature importance of machine learning has risen recently in the microbiome landscape with its high predictive performance in several disease states. To have a concrete selection among a high number of features, Recursive Feature Elimination (RFE) has been widely used in the bioinformatics field. However, machine learning based RFE has factors that decrease the stability of feature selection. In this paper, we suggested methods to improve stability while sustaining performance.

Results

We exploited the abundance matrices of the gut microbiome (283 taxa at species level and 220 at genus level) to classify between patients with inflammatory bowel disease (IBD) and healthy control (1569 samples). We found that applying an already published data transformation before RFE improves feature stability significantly. Moreover, we performed an in-depth evaluation of different variants of the data transformation and identify those that demonstrate better improvement in stability while not sacrificing classification performance. To ensure a robust comparison, we evaluated stability using various similarity metrics, distances, the common number of features, and the ability to filter out noise features. We were able to confirm that the mapping by the Bray-Curtis similarity matrix before RFE consistently improves the stability while maintaining good performance. Multi-Layer Perceptron (MLP) algorithm exhibited the highest performance among eight different machine learning algorithms when a large number of features (a few hundred) were considered based on the best performance across 100 bootstrapped internal test sets. Conversely, when utilizing only a limited number of biomarkers as a tradeoff between optimal performance and method generalizability, the random forest algorithm demonstrated the best performance. Using the optimal pipeline we developed, we identified fourteen biomarkers for IBD at the species level and analyzed their roles using SHapley Additive exPlanations.

Conclusion

Taken together our work showed not only how to improve biomarker discovery in the metataxonomic field without sacrificing classification performance, but also provided useful insights for future comparative studies.

Keywords: microbiota, machine learning, feature selection, biomarkers discovery, Shapley values

1. Introduction

Next-Generation Sequencing technologies allow reconstructing the internal composition of the whole microbial community (microbiota) present in a sample possibly exploiting two different approaches:

Whole Genome Shotgun sequencing (WGS) and targeted amplicon sequencing of 16S ribosomal RNA (16S rDNA-seq) [**REF1#-REF2#**]. The first focuses on all genomes, while the second only on a region of a single gene, i.e. 16S rRNA gene. Different bioinformatics pre-processing pipelines can be used to obtain the so-called abundance matrix and taxonomy matrix [**REF3#-REF4#**] from raw-read data. The abundance matrix describes the relative abundance of different operating taxonomic units (OTUs) or of different Amplicon Sequence Variant (ASV) [**REF5#-REF6#**] on each sample, while the taxonomy matrix contains information about the taxonomy of each OTU or ASV, i.e. kingdom, phylum, class, order, family, genus, species.

Machine learning (ML) can be used to classify samples based on their taxa composition, thus identifying a microbial signature that characterizes host phenotypes. Recently, several studies exploited different ML-based techniques to develop models for predicting disease states, such as inflammatory bowel disease [**REF7#-REF8#**], colorectal cancer [**REF9#-REF10#**], and cardiovascular disease [**REF11#**] using microbial data. This growing interest is mainly due to the potential impact on diagnosis and therapeutic target identification.

However, the application of ML methods to microbiota might be challenging for a number of reasons [**REF12#**]. Despite the existence of several consortium studies [**REF13#-REF14#-REF15#-REF16#**], there's a lack of standards in data structures, metadata collection, and pre-processing. This leads to limited generalizability [**REF17#-REF18#**] of the results in terms of diagnostic and prognostic biomarkers, i.e. taxa that could be used as diagnostic or prognostic markers [**REF19#**] and that, as such, need to be robust and reproducible indicators of the biological state.

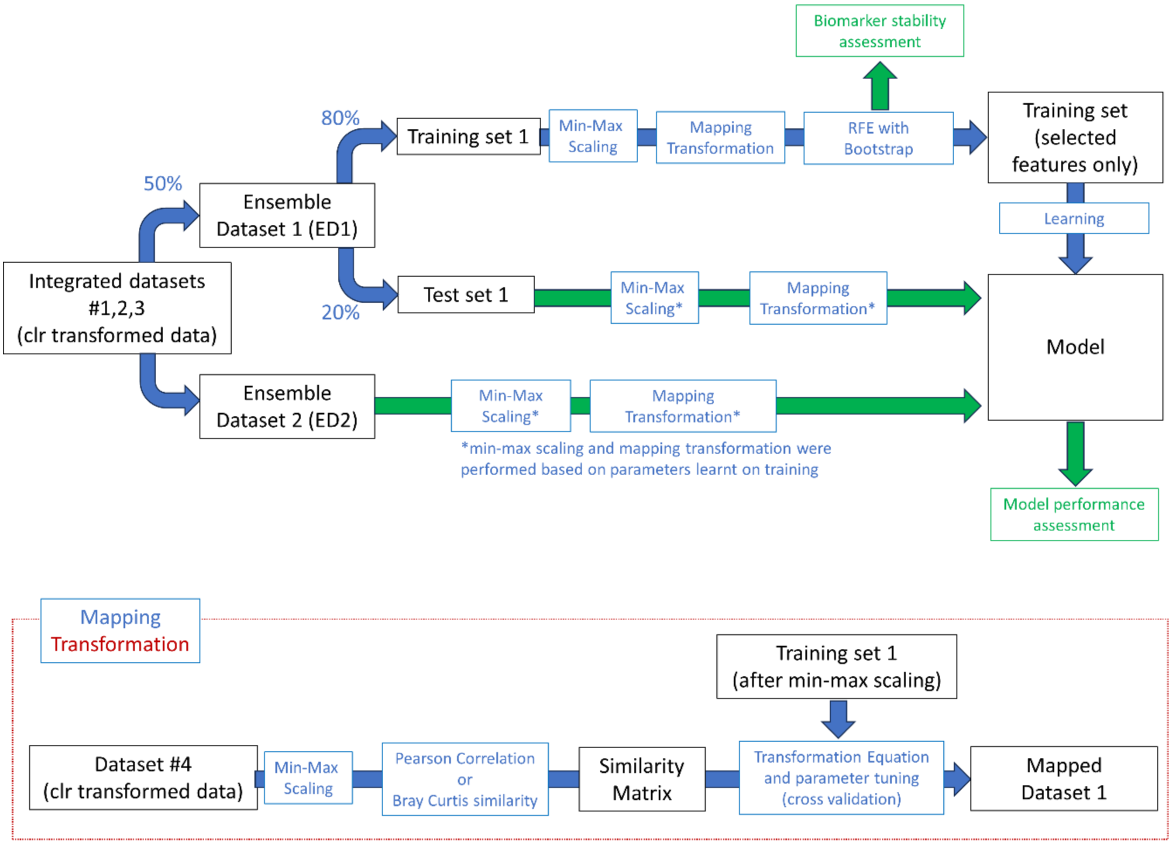

Fig. 1. Diagram for the overall experiments. The figure reports the analysis performed on ensemble dataset 1 using ensemble dataset 2 only for testing (black: inputs and outputs; blue: analysis steps; green: results assessment). A symmetric analysis was performed on ensemble dataset 2 using ensemble dataset 1 for testing. The inset represents the details of the mapping transformation procedure.

In this work, we performed a comprehensive evaluation of biomarker stability and classification approaches in the context of gut metataxonomic data used to identify a microbial signature of IBD-affected patients vs. healthy controls. Fig. 1 summarized the whole analysis pipeline. Three different datasets (described in Section 2.1) were merged to increase the number of examples. The final merged dataset consists of 1569 samples in total, where 702 samples were identified as IBD-affected patients and the others as healthy controls. The merged dataset was split in two, thus obtaining Ensemble Dataset 1 (ED1) and Ensemble Dataset 2 (ED2) by mixing the samples from the original studies. Note that, although the percentage of samples from different datasets is similar in ED1 vs. ED2, we expected the

abundance of each taxon to be different in the two datasets, due to the high number of features and the high variability and sparsity of metagenomics datasets **[REF20#]**. This characteristic might potentially affect the performance and generalizability of methods, which is a property we wanted to check in our experiments.

Each dataset was given as input to a “typical” ML pipeline, including splitting the data into test and training set, feature selection, and a final classification step. Since generalized biomarker discovery is a key point in this landscape, our effort focused on studying the feature selection step. In particular, we used Recursive Feature Elimination (RFE) within a bootstrap embedding [**REF21#**] as described in Section 2.2. to identify and select robust features. Moreover, the integration of prior knowledge in the learning process was investigated as a method to achieve higher stability of the biomarker list [**REF22#**]. A fourth external dataset for which the samples’ labels were not available, was used for this purpose, to compute feature similarity. We referred to this procedure as mapping strategy, since it is based on a kernel-based data transformation that projects data in a new space where the more similar two features are, the closer they are mapped in the new space. The basic idea is to take into account the correlation of different taxa: if some features are strongly correlated, then they likely have similar importance and are equally relevant for the classification task. More details about the mapping strategy are provided in Section 2.3. In Section 2.4 we introduce all metrics used to assess features stability (robustness of taxa selected).

It is worth noting that there are similar approaches that utilizes similarity matrix to map similar features into closer space **[REF23#][REF24#][REF25#][REF26#]**. Among others, AggMapNet utilizes a unique approach for data transformation, converting the original data into multi-channel 2D spatial-correlated images through pairwise correlation distances. This is achieved by employing the manifold learning method called Uniform Manifold Approximation and Projection (UMAP) **[REF27#]**. Through a preliminary clustering step, various channels are selected based on the pairwise correlation distances among features. Subsequently, the feature maps are fed as input to machine learning models, such as convolutional neural networks, enabling effective classification tasks. However, traditional ML

methods require as input 1D-vectors. Our approach can map unordered features as 1D vectors for conventional ML models and is therefore somehow complementary to AggMapNet approach.

With the selected set of features, we used different classification approaches to classify IBD-affected patients and healthy controls, namely logistic regression, support vector machines, random forests, extreme gradient boosting, and neural networks. In Section 2.5 we introduce all the prediction methods used in our study. To assess the generalizability of the classifiers and the robustness of the microbial signature, the models developed using ED1 were tested on test1 (obtained by splitting ED1 in training and test in proportions 80% and 20% respectively) and on the entire ED2. Similarly, models developed using ED2 were tested on test2 and on the entire ED1.

Using the Bray-Curtis similarity matrix to map the data provides the best, improved stability, without sacrificing the classification performance. Using this pipeline, we selected the top 14 features as a tradeoff between optimal performance and method generalizability. The best-performing algorithm on this robust set of features was the Random Forest. We further investigated the role of these biomarkers using Shapley values **[REF28#]**[**REF29#**][**REF30#**].

To ensure the reproducibility of the results, datasets and all code written in support of this publication are publicly available at GigaScience Database [REF55#].

2. Methods

2.1 Data

Four datasets were downloaded from Qiita, an open-source microbial study management platform, (see Table 1 for the reference Qiita study ID for reproducibility) [**REF16#, REF31#, REF32#, REF33#**]. Table 1 summarizes the characteristics of the different datasets, such as 16S region sequenced, the specimen analyzed, the patient's geographic origin, and the abundance matrices' dimensionality.

Throughout the manuscript, we will refer to each dataset with the ID indicated in the first column (Dataset#).

We downloaded the metadata file, with all the available covariates (e.g.: sample ID, age, sex, weight), the *.biom* file with the abundance matrices of the processed 16S rDNA sequences, and the associated taxonomy. For all the datasets, the abundance matrices were obtained by applying the same bioinformatics pre-processing steps, i.e. trimming (QIIMEq2 1.9.1) and Pick closed-reference OTUs (QIIMEq2 1.9.1) [**REF34#**] as implemented in Qiita.

Table 1. Summary of datasets used in the study

**Datasets Information**							
**Dataset#**	**Qiita study ID**	**16S Region**	**biological specimen**	**Geographic localization**	**Selected samples for the study**		
					**Total**	**IBD**	**nonIBD**
Dataset 1 [**REF16#**]	11484	V4	Feces	USA	96	95<br>CD:75/UC:20	1
Dataset 3 [**REF31#**]	2151	V4	Feces	USA	836	32<br>CD:10/UC:22	804
Dataset 2 [**REF32#**]	1629	V4	Feces	Sweden	637	575<br>CD:251/UC:324	62
Dataset 4 [**REF33#**]	10317	V4	Feces	USA, UK, AU	444	N/A	N/A

Ensemble Datasets Information (Dataset #1+Dataset #2+Dataset #3)			
Ensemble Dataset #1 -Training Dataset	627	281	346
Ensemble Dataset #2 -Training Dataset	628	281	347
Ensemble Dataset #1 -Test Dataset	157	70	87
Ensemble Dataset #2 -Test Dataset	157	70	87

Table 1. Summary of datasets used in the study. The table shows, in different columns: the ID of the dataset (Dataset#), the Qiita study ID, the sequenced hypervariable region (16s Region), the specimen analyzed (Product), the patient's geographic origin (Geographic localization), and the number of samples labelled as IBD vs non-IBD. CD stands for Crohn's disease and UC for Ulcerative Colitis

Several preprocessing tools have been developed to handle with 16S seq data characteristics, such as log-ratio-based transformation, normalization, and zero-imputation **[REF35#-REF36#-REF37#]**. However, no standard approach has been identified yet. We pre-processed each dataset independently and then combined them in ED1 and ED2. First, all the taxa with the same taxonomy classification (i.e. same species level or genus level) were aggregated and their respective counts were summed, as usually done in microbiome studies [**REF19#-REF38#-REF39#**]. We considered both species and genus levels independently, thus obtaining two abundance matrices for each dataset. After that, we filtered out those taxa with more than 99% of abundances equal to 0. It is worth noting that usually, in the field of microbiota analysis, a threshold of 95% or 90% is used. However, lower thresholds can alter the composition of the abundance profiles (i.e. the abundances of all the features in a sample) and have an effect on the following normalization step [**REF40#**]. Then we divided the abundance profiles by the geometric mean and took the log (base 2) of the ratios leveraging on the idea of clr transformation, using a pseudocount value equal to the minimum observed data.

After finishing preprocessing Dataset#1+Dataset#2+Dataset#3 separately, we integrated the three datasets as shown in Fig.1. To this purpose, from the entire set of features, only the ones common throughout all datasets were kept. In this way, we selected a core set of features with 283 taxa at the species level and 220 at the genus level. After datasets splitting in ED1 and ED2, min-max scaling was performed to scale features into the same range. The preprocessing was performed separately to the external dataset (Dataset #4) following the same steps described above.

2.2 Feature Selection Approach

For each taxonomic level (genus, species) and each dataset ED1 and ED2, Recursive Feature Elimination (RFE) was used as a feature ranking method to obtain the optimal number of features [**REF21#**] as described in the following. RFE was performed 100 times with bootstrapping. In each bootstrap, the training dataset was split into an internal training set and an internal test set. A prediction model was trained within the internal training set using Linear Support Vector Machine (SVM). Recursive feature elimination (RFE) is a feature selection method that fits a model and removes the weakest feature (or features) until the specified number of features is reached. Linear SVMs were chosen because of their cost-efficiency tradeoff and their ability to deal with a high number of predictors [REF41#] [REF42#] [REF43#]. The regularization parameter was tuned within each internal training set using grid search and 5-fold cross-validation with Matthew correlation coefficient (MCC) [**REF44#**] as the performance index. Feature importance was measured based on the feature weight since data had been standardized in input. The least important feature was eliminated from the dataset iterating the process until only one feature was left. Sorting the features from the last eliminated, which will therefore have rank 1, and averaging the ranked lists across the 100 bootstraps, the global feature rank was calculated. Finally, MCC was computed independently on each bootstrap internal test sets for different numbers of features and then averaged across the 100 bootstraps. The number of features corresponding to the maximum MCC (MCC shows either a peak or a saturation effect) was taken as optimum.

RFE was performed i) without any transformation; ii) with mapping performed using Pearson correlation; iii) with mapping performed using Bray-Curtis similarity, as explained in the following paragraph.

2.3 Mapping

2.3.1 Similarity Matrix

The similarity matrix is a symmetric matrix encoding the knowledge about the likeness between features. In this paper, we used either Pearson correlation [**REF45#**] or Bray-Curtis similarity [**REF46#**]. Pearson correlation is the linear correlation between two sets, dividing the covariance of two features by the product of each standard deviation. Bray-Curtis similarity is the comparison of the composition between two different sites, dividing twice the sum of the lesser value for only those features in common between two sites by the total sum of values counted at both sites for all the features. Assume x_{ij} as $j^{th}(< n)$ feature value of $i^{th}(< m)$ data sample. Then we can define the list of ath and b^{th} feature value as $A = [x_{1a}, x_{2a}, \dots\ x_{ma}]$ and $B = [x_{1b}, x_{2b}, \dots\ x_{mb}]$. Pearson correlation and Bray Curtis similarity are defined as follows:

$$Pearson\ Correlation = \frac{cov(A, B)}{\sigma_A \sigma_B}$$ Eq. (1)

$$Bray\ Curtis\ Similarity = \frac{2\sum_1^m min(A[i], B[i])}{(\sum A) + (\sum B)}$$ Eq. (2)

It should be noted that, to avoid introducing any bias, the similarity matrices were calculated on dataset 4 (see Fig. 1 and Table 1), an external dataset used only to calculate the similarity matrix to perform the mapping transformation (see paragraph 2.3.1).

2.3.2 Mapping transformation and its theoretical advantages

Feature mapping is a crucial steps in machine learning that can significantly impact model performance. Feature mapping involves transforming raw input data into a format suitable for the learning algorithm, enabling the extraction of meaningful patterns and relationships. By converting complex and diverse features into a more structured representation, feature mapping empowers the model to discern relevant information, leading to more accurate predictions. In the context of omics data, due to the abundance of features, the problem of identifying relevant features for the predictive model becomes under-constrained, leading to numerous potential sets of relevant features that could achieve comparable accuracy. To address this, we leveraged supplementary data from an external dataset (dataset 4) to impose additional constraints during feature mapping. In essence, this approach aims to account for strong correlations among certain features, indicating their similar importance for the classification task. As a result, we ensure that these correlated features are equally relevant, enhancing the overall performance of the model.

Information about feature correlation is integrated by mapping data using a kernel transformation that has been shown to possibly alleviate feature instability [**REF22#**]. Transformation matrix P, is obtained using the equation $\mathrm{P} = \mathrm{D}^{-1}(\mathrm{I} + \alpha(\mathrm{S} - \mathrm{I}))$, where S is the similarity matrix, D is the diagonal matrix whose elements are the sum of the elements in the rows of the matrix $\mathrm{I} + \alpha(\mathrm{S} - \mathrm{I})$ and α is a tuning parameter. The value of α was decided for each experiment using 5-fold cross-validation within its internal training dataset using a grid of 0.01 and from 0.05 to 1 by step 0.05. In our approach, mapping was used only in the RFE step.

2.4 Evaluation metrics

2.4.1 Stability

Different rank-based stability indexes were used to evaluate the robustness of the feature selection algorithm: Spearman's rank correlation coefficient (SRCC), Hamming Distance, Pearson Correlation, and Bray Curtis Dissimilarity [**REF47#-REF48#**].

Since the ranks are distinct integers, ***SRCC*** between two rank sets can be calculated as follows:

$$SRCC = 1 - 6 * \sum_{each\,feature} \frac{d^2}{n^3 - n}$$

Eq. (3)

Where d is the difference between the two ranks of each feature and n is the total number of ranked features.

Hamming Distance between two rank sets is calculated as the proportion of disagreeing components as follows:

$$Hamming\ Distance = \frac{1}{n} \sum_{each\,feature} \begin{cases} 1, & if\ ranks\ are\ not\ the\ same \\ 0, & otherwise \end{cases}$$

Eq. (4)

The equation of ***Pearson Correlation and Bray Curtis Dissimilarity*** is identical to Eq. (1) and Eq. (2), but A and B are now comprised of rank of each feature at each bootstrap where x_{ij} represent the rank of $i^{th}(i = 1, \dots, \#of\ features)$ feature of $j^{th}(j = 1, \dots, 100)$ bootstrap. Bray Curtis Dissimilarity is calculated by subtracting Bray Curtis similarity value from one.

Euclidian distance is the length of the line segment connecting two different points. The stability indexes described above were computed for each pair of bootstrap samples used for RFE and finally averaged across the 100*(100-1)/2 values.

The ***number of commonly ranked features*** was defined by counting the number of common features across all bootstraps considering a range that starts from the top rank single feature and expanding the range one by one. The condition can be varied by defining ‘common’ as consistent across all 100 bootstraps or across at least 66 or 50 bootstraps.

To compare the ability to distinguish unimportant features from important features, a noise filtering experiment was performed. We added 100 randomly generated noisy features (range 0 to 1 from a uniform distribution) to the original features (range 0 to 1 by min-max scaler) before mapping and after

mapping and RFE compared the average rank of noisy features from the average rank of true features (Table 3). With a good noise filtering algorithm, noise features are expected to have big feature ranks.

2.4.2 Performance Measurement

MCC was used for the step of Recursive Feature Elimination and to compare the performance of the different models. MCC estimates the correlation between the predictive value and ground truth value by using the following equation:

$$MCC = \frac{TN*TP-FN*FP}{\sqrt{(TP+FP)(TP+FN)(TN+FP)(TN+FN)}} \qquad \text{Eq. (5)}$$

where TP is the number of True Positive, TN of True Negative, FN of False Negative and FP of False Positive values. David et al. showed the appropriateness of MCC for evaluating binary classification performance by considering all four confusion matrix categories [**REF44#**].

AUC, accuracy, specificity, sensitivity, positive predictive value (PPV), negative predictive value (NPV) were also measured to compare models' performance. AUC stands for area under the ROC curve, and the ROC curve is drawn by placing False Positive Rate, $\frac{FP}{FN+TP}$ to x-axis and True Positive Rate, $\frac{TP}{TP+FN}$ to y-axis. Accuracy stands for $\frac{TP+TN}{TP+FP+FN+TN}$, specificity for $\frac{TN}{TN+FP}$, sensitivity for $\frac{TP}{TP+FN}$, PPV for $\frac{TP}{TP+FP}$, NPV for $\frac{TN}{TN+FN}$. Apart from Linear SVM which outputs the class, predictive values should be converted into binary values by establishing a threshold. Considering the positive to negative ratio was not high (with a value of 0.81 as indicated in Table 1), we set the threshold to 0.5.

2.5 Classification Model Algorithms

Eight different types of prediction algorithms, namely logistic regression, linear SVM, random forest, XGBoost, Perceptron, and Multi-Layer Perceptron (MLP) with 1, 2 or 3 hidden layers, were used to classify samples in IBD vs. healthy using the features selected within the RFE phase. Within each

training dataset, a classification model which predicts the status of IBD was built. Hyperparameters in each model were tuned using 5-fold cross-validation within the training set with grid search.

Logistic Regression outputs prediction scores between 0 and 1 by applying logistic function to linear regression. In this study, regularized logistic regression, using L2 penalty, i.e. Ridge Regression, was used. For the optimization, lbfgs solver was used with the tolerance parameter of 1e-4 (which followed the default setting of the library sklearn.linear_model.LogisticRegression by scikit-learn) [REF49#]. Regularization parameter was tuned using a grid of two to the power of (-5, -3, -1,1,3,5).

Linear SVM linearly separates data maximizing the margin between two classes. The squared L2 penalty was applied to prevent overfitting. 'rfb' kernel was used, and 'gamma' was set as 'scale' so that it used 1 / (# of features * variation of features) as value of gamma (which followed the default setting of the library sklearn.svm.LinearSVC by scikit-learn) [REF49#]. Regularization parameter was tuned with a grid of two to the power of (-5 to 15 with the step of 2).

Random Forest builds many decision trees and uses bagging to make an uncorrelated forest of trees combined for better prediction. Gini-impurity was used to decide the splits, while the maximum depth was unlimited so that nodes are expanded until all leaves are pure or contain less than the parameter 'min samples split'. The parameter 'min samples split', which is the minimum number of samples for splitting an internal node, and the parameter 'min samples leaf', which is the minimum of samples to be at a leaf node were set equal to two. The number of features to search for the best split was set as $\sqrt{\#\text{ of features}}$. Finally, the number of trees in the forest was set as 100.

Extreme gradient boosting (***XGBoost***) is a tree-ensemble model which utilizes gradient boosting to combine many trees. XGBRegressor was trained with 200 gradient boosted trees. Base learners had 20 maximum tree depth. Subsample ratio of the training instance was 0.2, while subsample ratio of columns when constructing each tree was 0.5. Minimum loss reduction which is for the partition on a

leaf node, was set to 1. Learning rate was tuned with the grid of 0.005,0.01, 0.05, 0.1, 0.5. Regularization factor, alpha, which is the L1 regularization term was with the grid of 1e-3, 1e-2, 0.1, 1,10.

Perceptron is a single layer neural network, predicting an output with combinations of input values, weights and bias. Multi-Layer Perceptron (***MLP***) is a feedforward neural network with multiple hidden layers. In the experiments, MLP Regressor which has a single output layer was built. LBFGS optimizer minimizing the squared error function was used to optimize weight variables, while ReLu function was utilized for the activation function. When the loss score was under 1e-4, the optimization was considered as converged, and the training stopped. Learning rate and L2 regularization term are tuned with the same procedure with XGBoost. MLP with 1,2,3 hidden layers were constructed with hidden layer size corresponding to the number of features.

3. Results

3.1 Mapping Transformation and Recursive Feature Elimination

Hyperparameter α used in mapping (paragraph 2.3.2) decides how much we weigh the similarity matrix in data transformation when performing RFE and was tuned in cross-validation within each internal test set as explained in paragraph 2.3.2. (see Fig. 2). Interestingly, the MCC values exhibit different patterns when comparing mapping performed using Pearson Correlation and mapping performed using Bray Curtis Similarity. For mapping with Pearson Correlation, the MCC shows an unstable profile and a sudden drop as shown in Fig 2.a and c. The optimal α value appears as the smallest value, 0.01, within the grid range of 0.01 to 1. On the other hand, Bray-Curtis Similarity mappings demonstrates an initial increase in the performance followed by a stable pattern of decline, with optimal α values equal to 0.15 and 0.05 at species level for ED1 and ED2, respectively; and equal to 0.05 at genus level for both ED1 and ED2.

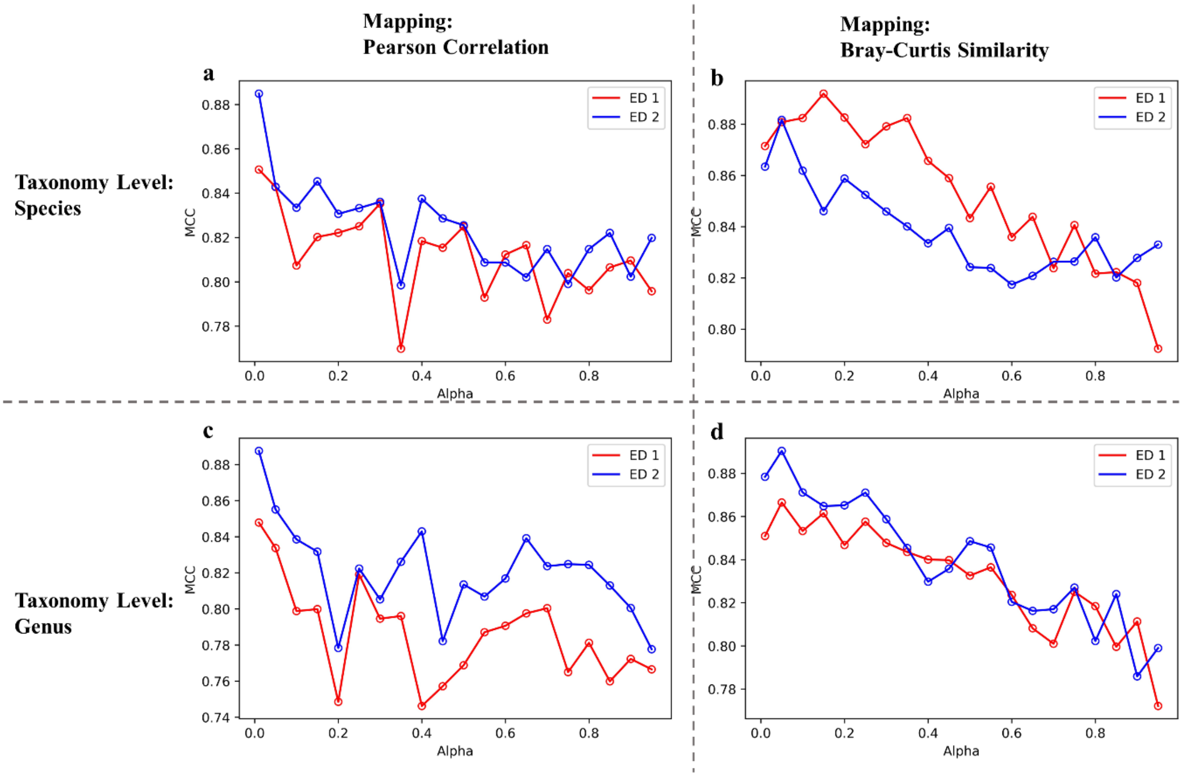

Fig. 2. Average MCC across bootstraps at different values of hyperparameter α in mapping transformation. Hyperparameter α is used for controlling the degree of mapping transformation. Panel a: species level, Mapping by Pearson Correlation. Panel b: species level, Mapping by Pearson Correlation. Panel c: genus level, Mapping by Pearson Correlation. Panel d: genus level, Mapping by Pearson Correlation

Performance dependence on the number of features is illustrated in Fig. 3, where MCC is shown i) with not mapping; ii) with mapping performed using Pearson correlation; iii) with mapping performed using Bray-Curtis similarity. The pattern is similar in the three cases, with MCC close to 0.8 slightly increasing with the number of features and saturating only at the very right side of the curve. As a consequence, the optimal number of features is high (87.1% of features are selected in average) in most of RFE experiments. the only exception is represented by mapping based on Pearson correlation on ED1 at species level, which reaches the maximum MCC when using 22 features. The maximum value of the MCC (average calculated on the 100 bootstrap samples) is shown in the label of Fig. 3 as 'Maximum MCC', with the corresponding number of features indicated as “Optimal feature number”.

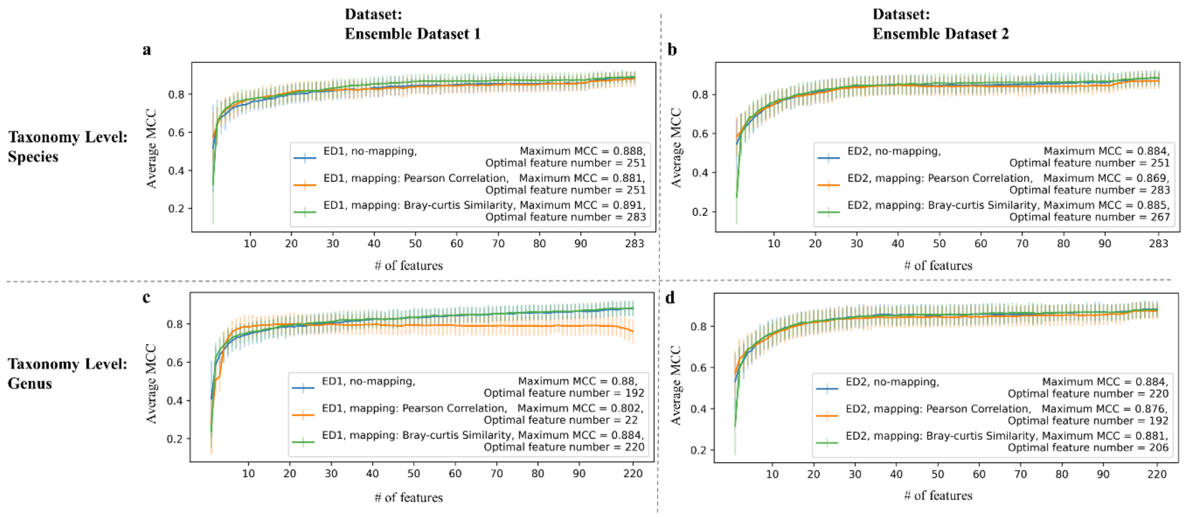

Fig. 3. Average MCC across the 100 bootstrapped recursive feature elimination process. MCC(y-axis) at varying number of features(x-axis) averaged across the 100 bootstrap internal test sets. Maximum MCC with optimal feature number is written in each legend. Error bars represent the standard deviation of MCC. Panel a: species level, ED1. Panel b: species level, ED2. Panel c: genus level, ED1. Panel d: genus level, ED2.

3.2 Feature stability

Stability Indexes

Table 2 illustrates the values of the stability metrics across the different bootstraps in each experiment. At species level, mapping data using Bray-Curtis similarity allows reaching better stability with every dataset and every metric. At genus level, mapping data using Bray-Curtis similarity still shows the better stability in ED1 dataset (with the exception of Hamming distance and SRCC metrics) and in ED2 dataset (with the exception of Hamming distance metric).

Supplementary Fig. 1 and 2 show the *number of commonly ranked features* across all 100 bootstraps or across at least 66 or 50 bootstraps. At small ranks, the difference between different mapping strategies is not clear as the number of common features across bootstraps is too small. However, as more features are considered, RFE with Bray Curtis similarity-based mapping shows a greater number of common features in every dataset except ED1 in genus level.

Table 2. Stability metrics for each Recursive Feature Elimination experiment

Level: Species					
ED1	SRCC	Pearson Correlation	Hamming Distance	Bray-Curtis Dissimilarity	Euclidian Distance
no-mapping	0.662	0.653	0.919	0.085	1.402
mapping: Pearson Correlation	0.652	0.642	0.92	0.086	1.425
mapping: Bray-Curtis Similarity	**0.667**	**0.689**	**0.918**	**0.08**	**1.325**
ED2					
no-mapping	0.617	0.639	0.925	0.088	1.423
mapping: Pearson Correlation	0.579	0.599	0.928	0.094	1.505
mapping: Bray-Curtis Similarity	**0.643**	**0.662**	**0.921**	**0.084**	**1.355**
Level: Genus					
ED1	SRCC	Pearson Correlation	Hamming Distance	Bray-Curtis Dissimilarity	Euclidian Distance
no-mapping	**0.67**	0.671	0.921	**0.1**	1.689
mapping: Pearson Correlation	0.607	0.618	**0.903**	0.108	1.819
mapping: Bray-Curtis Similarity	0.667	**0.677**	0.919	**0.1**	**1.672**
ED2					
no-mapping	0.698	0.715	**0.916**	0.093	1.561
mapping: Pearson Correlation	0.647	0.662	0.922	0.102	1.705
mapping: Bray-Curtis Similarity	**0.707**	**0.733**	0.917	**0.089**	**1.514**

Table 2. Stability metrics for each Recursive Feature Elimination experiment. For each dataset, the best stability value is highlighted in bold.

Noise Filtering

To assess the noise filtering ability of the various approaches (paragraph 2.4.1), we subtracted the average rank of noisy features from the average rank of original data features (283 features in species level, 220 features in genus level). In Table 3, except ED1 in species level, mapping with Bray-Curtis similarity shows better noise filtering score than the others. The superiority of mapping with Bray-

Curtis similarity is more apparent at genus level, as the gap is triple bigger compared to species level (22.87 to 77.66 and 26.06 to 80.49). While mapping with Bray-Curtis similarity shows consistent noise filtering ability, mapping with Pearson correlation shows unstable noise filtering ability, which is sometimes worse than no-mapping.

Table 3. Noise filtering result for each Recursive Feature Elimination experiment.

Noise Filtering Score	avg(rank of noises) – avg(rank of real) *rank by Recursive Feature Elimination	
Taxonomy Level / Algorithm	species	genus
ED 1: no-mapping	11.30	34.61
ED 1: mapping with Pearson Correlation	27.17	-5.92
ED 1: mapping with Bray Curtis Similarity	22.87	77.66
ED 2: no-mapping	20.19	33.21
ED 2: mapping with Pearson Correlation	14.10	33.42
ED 2: mapping with Bray Curtis Similarity	26.06	80.49

Table 3. Noise filtering result for each Recursive Feature Elimination experiment. The margin between the rank of noise features and original features is calculated.

3.3. Performance comparison by selected set of features

Prediction models were built based on the optimal set of features identified using linear SVM based RFE and Bray-Curtis-similarity based mapping (Fig. 3). To validate the methods, the models trained on ED1 were tested in both Test1-ED1 and on the entire ED2 and models trained on ED2 were tested on Test2-ED2 and on the entire ED1. 8 different prediction algorithms (see Section 2.5) were tested. Overall performance obtained by different methods using different metrics (AUC, accuracy, sensitivity, specificity, PPV, NPV, MCC) are illustrated in Supplementary Table 1,2,3 and 4. Supplementary Table 5 summarizes the best MCC with best performing algorithm in each experiment. MLP with 1 or 2 hidden layer shows the best MCC in most of the experiments (23 among 24 results).

As the optimal number of features is high in most of RFE experiments (Fig.3.), prediction models were also trained and compared with a constant number of a few top features. We choose to consider the top 14 features (see paragraph 3.4). Results obtained using different algorithms are shown in Supplementary Table 6. With smaller number of selected features, the performance slightly decreases and the best performing algorithm varies. Random Forest shows the best performance in the most cases (15 among 24), followed by MLP-1,2 hidden layer, XGBoost, Logistic Regression and Linear SVM. RFE without mapping shows better performance than RFE with mapping by with Bray-Curtis similarity with 0.0016 in average. Considering the small gap, mapping with Bray Curtis Similarity, while improving feature stability, does not seem to affect methods performance in terms of MCC, which overall, remains quite stable.

3.4 Full Data Experiment and Biomarker Selection

The best pipeline for biomarker selection (Linear SVM based RFE with mapping using Bray-Curtis Similarity) is applied to the full dataset at species level without any split (i.e. training set is the combination of training sets in ED1 and ED2, test set of test sets in ED1 and ED2) to obtain the possible biomarkers for IBD. During the RFE process, we compute the average MCC across the 100 bootstraps internal test sets. Similar to Fig.3, average MCC slightly increases with the number of features and the maximum MCC is reached corresponding to 267 features among the 283 considered. The eight different predictive models presented in Section 2.5 are trained using the selected features, illustrated in Supplementary Table 7. MLP-1,2 hidden layers consistently show the highest performance in terms of MCC, confirming what previously found.

As the optimal number of features is high in most of RFE experiments (Fig.3.), prediction models were also trained and compared with a constant number of a few top features. We choose to consider the top 14 features as a tradeoff between optimal performance and generalizability potential, based on results

shown in Figure 4 where we calculated the ΔMCC as the difference in MCC at different subsequent numbers of features and selected the value at which the ΔMCC starts to converge to zero. Compared to models with optimal number of features (267), overall performance decreases and the best model algorithm changes. While MLP with 1 hidden layer is the best algorithm with MCC 0.963 in Supplementary Table 7, it decreases to 0.826. Instead, Random Forest decreases to 0.845 from 0.854, which is the maximum MCC among the eight algorithms.

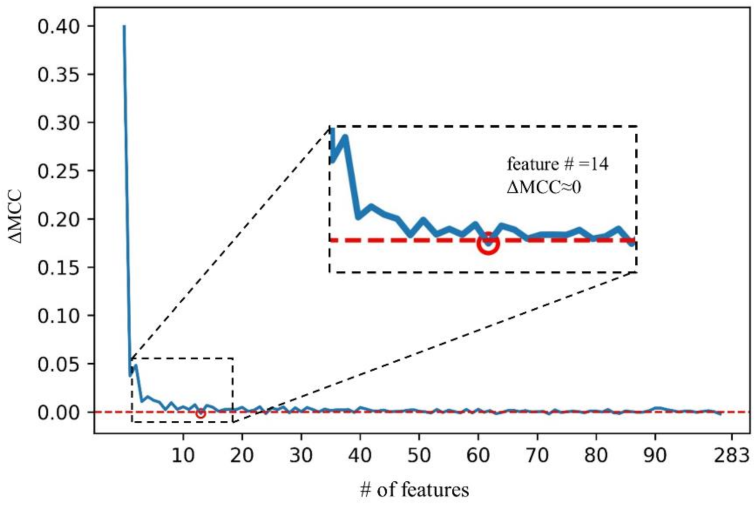

Fig. 4. Differential of average MCC during RFE. Average MCC is calculated in each feature number, and each average MCC is subtracted by its former average MCC to calculate differentials. x-axis: the number of features, y-axis: differential of average MCC, horizontal red line: y=0.

With the final random forest based predictive model, Shapley values are calculated on the training set, the test and the external dataset for the top 14 ranked features. Shapley values calculate the extent that each feature at each data sample contributes to the change of output of the prediction model. Since Shapley values are model agnostic which provides local explanations, they are calculated on the training and test datasets and on dataset 4 (for which we do not know the sample labels but still can run the models and calculate the Shapley values). Shapley values on the training set is shown in Fig. 5 (Shapley values on the test and the external dataset 4 are shown in Supplementary Table 8). The rank of the 14

features based on Shapley values shows a high similarity across different datasets (training, test and external dataset 4), and the directions which increases or decreases the possibility of IBD are identical. Random Forest model with 14 selected features determines *Lachnospiraceae(f) Butyrivibrio(g), Bacteroides (g) fragilis (s), Porphyromonadaceae(f) Dysgonomonas(g), Erysipelotrichaceae(f) cc_115 (g), Fusobacteriaceae(f) Fusobacterium(g), Alkalimonas(g) amylolytica(s), Lactobacillus(g) zeae (s), Peptococcaceae(f) Peptococcus (g)* as indicators of IBD state, and *Corynebacteriaceae (f) Corynebacterium (g), Tissierellaceae(f) WAL_1855D(g), Campylobacteraceae(f) Campylobacter(g), Lachnospiraceae(f) Ruminococcus(g), Porphyromonadaceae(f) Parabacteroides(g), Lactobacillus(g) iners (s)* as indicators of negative of IBD (*f*: family, *g*: genus, *s*: species level).

Indicators of IBD

Total Rank	Taxonomy name	SHAP values
#3	Lachnospiraceae(f) Butyrivibrio(g)	
#4	Bacteroides (g) fragilis (s)	
#6	Porphyromonadaceae(f) Dysgonomonas(g)	
#8	Erysipelotrichaceae(f) cc_115 (g)	
#10	Fusobacteriaceae(f) Fusobacterium(g)	
#11	Alkalimonas(g) amylolytica(s)	
#13	Lactobacillus(g) zeae (s)	
#14	Peptococcaceae(f) Peptococcus (g)	

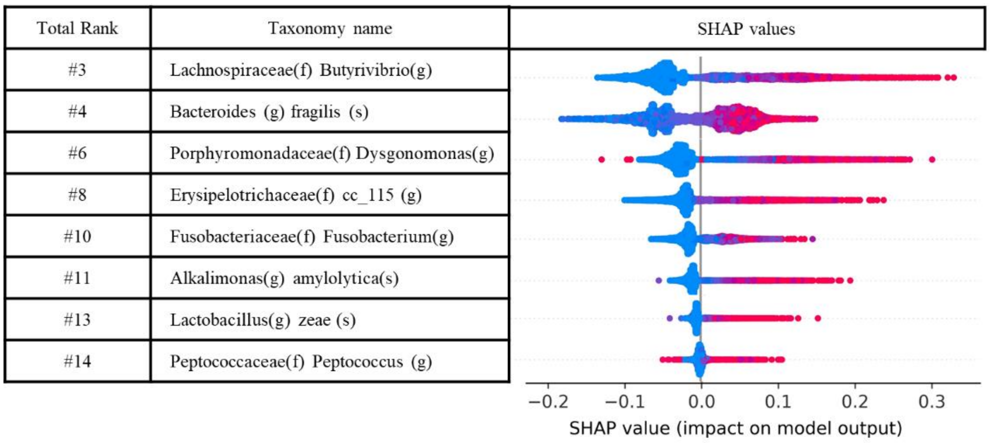

Indicators of non IBD

Total Rank	Taxonomy name	SHAP values
#1	Corynebacteriaceae (f) Corynebacterium (g)	
#2	Tissierellaceae(f) WAL_1855D(g)	
#5	Campylobacteraceae(f) Campylobacter(g)	
#7	Lachnospiraceae(f) Ruminococcus(g)	
#9	Porphyromonadaceae(f) Parabacteroides(g)	
#12	Lactobacillus(g) iners (s)	

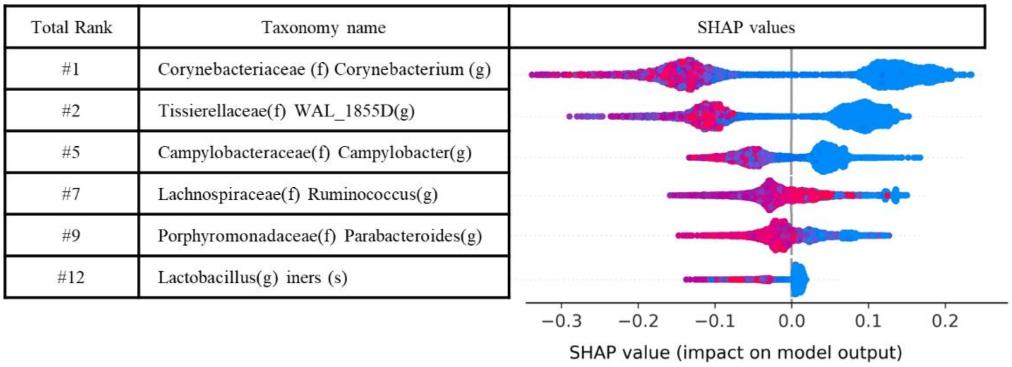

Fig. 5. SHAP summary plot of primary indicators of IBD and non IBD. Random Forest model trained by top fourteen features in RFE (species level, trained by the combination of training sets in ED1 and ED2) are used to calculate SHAP values. SHAP values are measured by training dataset. Total rank is the rank between fourteen features used for training the model.

4. Discussion

Stable feature selection is a prerequisite to decide strong biomarkers. To prove the validity of a set of features as biomarkers, they should be observed consistently every time a feature selection algorithm is applied. In this paper, we performed Recursive Feature Elimination multiple times using bootstrapping and checked the level of consistency of feature ranks between trials using stability indexes. Moreover, we investigated various strategies which could improve the stability of selected biomarkers.

First, we used bootstrap to cope with the scarcity of samples in the training set compared to the number of features **[REF50#]**. In this way we generated various classifiers, selected various features on various data splits, and then averaged the results, preserving a high ranking only for those features that are consistently the most discriminating features across the splits. Second, since the high number of features makes the problem under-constrained, i.e. many possible sets of features can be considered relevant to the task and equally good in terms of accuracy, we used additional information from an external dataset (dataset 4) to include additional constraints in terms of feature mapping. In other words, if some features are strongly correlated, then they likely have similar importance, and we want them to be equally relevant for the classification task.

Three different datasets were merged to increase the number of examples and mitigate the potential batch effect, and then split in two. As the division of the dataset was performed using bootstrapping, the ratio of non-IBD vs IBD is preserved, and the ratio of the data source was also preserved without significant difference as shown in Supplementary Table 9. However, the distribution of each feature may differ as the size of the dataset was small compared to the number of features.

Mapping transformation with Linear SVM based RFE improved the stability without the loss of performance. We recommended using Bray-Curtis similarity to improve the stability of the selected features since, in most cases, mapping using Bray-Curtis similarity showed significant increase of stability compared to other approaches and higher noise filtering score, at both genus and species level.

Moreover, as shown in Supplementary Fig 1 and 2, mapping with Bray-Curtis similarity presents a higher number of common features, already at early stage of RFE.

Notably, the Bray-Curtis similarity was designed for compositional data (a composition is represented by a vector of proportions with respect to a total sum on the sample under observation) and is therefore more robust to spurious correlations introduced by the fact that the taxonomic abundances are multivariate observations whose information content is closely linked to the relationship between the components.

A possible drawback of our method is that the mapping transformation compresses the information by shortening the distance between similar features. In case the degree of the transformation is high (α in the transformation matrix equation $P = D^{-1}(I + \alpha(S - I))$), the loss of potential information can be important. Choosing α in cross-validation as done in this work, might help mitigating this risk although at a higher computational cost. Indeed, in our experiments the optimal choice of α did not highlight a loss in classification performance.

There are similar approaches that utilizes similarity matrix to map similar features into closer space **[REF23#][REF24#][REF25#][REF26#]**. AggMapNet maps the original data into multi-channel 2D spatial-correlated images based on their pairwise correlation distances using the manifold learning method Uniform Manifold Approximation and Projection (UMAP) **[REF27#]**. Different channels are chosen based on a preliminary clustering step, which is again based on pairwise correlation distances between features. Features maps are then given as input to machine learning methods such as convolutional neural networks to perform classification. Results obtained using AggMapNet

[REF23#][REF24#] approach on our dataset are shown in Supplementary Table 10 and compared with our pipeline (Bray-Curtis similarity-based mapping + RFE + MLP). This comparison proves the strength of our pipeline in the situation of small data and a high number of features, which is typical of omics studies. Differently from AggMapNet **[REF23#][REF24#]**, our pipeline does not project the data in multi-channel 2D spatial-correlated images, thus it is not suitable for 2D data representation and omics data integration.

To further assess the consistency of our results and the robustness of our approach, we calculated the FDR-adjusted p-values using the Wilcoxon rank-sum test, fold change and average, standard deviation information of the top 14 biomarkers we selected in Supplementary Table 11. We found that 13 of 14 biomarkers had a q-value below 0.001 when comparing between IBD and non-IBD samples. However, despite the statistically significant differences in the markers, 52.3% of the features showed significant differences between IBD and non-IBD groups, which implies that relying solely on statistical tests may not be sufficient in determining good biomarkers. The robustness of our algorithm and the excellence of machine learning algorithms are shown by high performing prediction for this sparse type of dataset.

It should be reported that we originally performed the overall experiment without logarithmic transformation (data not shown) and checked later that logarithmic transformation improved the performance significantly. We expect the logarithmic transformation has accounted for the highly skewed distribution of microbial abundances **[REF51#]**. Before using the logarithmic transformation, the best performing algorithm was random forest instead of MLP. While tree-based ensembles utilize feature splitting based on a set of thresholds, neural network utilizes continuous values to build non-linear relationships among variables, and logarithmic transformation is expected to advantage the performance in this aspect.

However, when fewer features are considered, random forest outperforms other methods including MLP. In recent studies, random forest provides generally higher performance than other conventional algorithms for microbial data analysis [**REF38#-REF52#-REF53#-REF54#**]. We suppose the higher

complexity of MLP algorithm takes more advantage when a higher number of features is considered; whereas, in case fewer features are considered, the ‘split’ approach of tree-based ensemble models may fit to microbial data better.

Availability of Source Code and Requirements

1) Project name: MLonMicrobiome
2) Project home page: https://gitlab.com/sysbiobig/mlonmicrobiome
3) Operating system(s): Platform independent
4) Programming language: Python, R
5) Other requirements: R 4.1.3, phyloseq 1.27.6, openxlsx 4.2.4, Python 3.85, scikit-learn 1.0.2
6) License: GNU GPL
7) RRID: SCR_024442

Data Availability

Four datasets were downloaded from Qiita, an open-source microbial study management platform, (see Table 1 for the reference Qiita study ID for reproducibility) [**REF16#, REF31#, REF32#, REF33#**]. Data after each preprocessing stage can be accessed by our code repository. Our code and other data further supporting this work, including DOME-ML annotations, are openly available in the GigaScience repository GigaDB [REF# 55].

Abbreviations

RFE: Recursive Feature Elimination; Inflammatory bowel disease: IBD; Whole Genome Shotgun sequencing: WGS; Matthew correlation coefficient: MCC; Support Vector Machine: SVM; Random

Forest: RF; Extreme Gradient Boosting: XGBoost; Multi-layer perceptron: MLP; Spearman’s rank correlation coefficient: SRCC; Canberra Distance: CD; Area Under roc Curve: AUC;

Acknowledgement

This research was supported by a grant of the Korea Health Technology R&D Project through the Korea Health Industry Development Institute (KHIDI), funded by the Ministry of Health & Welfare, Republic of Korea (grant number : HI21C1092) and by the SEED Project "tRajectoriEs of baCtErial NeTwoRks from hEalthy to disease state and back (RECENTRE)" funded by the Department of Information Engineering of the University of Padova, Grants nr. DI_C_BIRD2020_01.

Competing Interests

The authors declare that they have no competing interests.

References

[REF1#] Quince C, Walker AW, Simpson JT, Loman NJ, Segata N. Shotgun metagenomics, from sampling to analysis. Nat Biotechnol. 2017 Sep 12;35(9):833-844. doi: 10.1038/nbt.3935. Erratum in: Nat Biotechnol. 2017 Dec 8;35(12):1211. PMID: 28898207.

[REF2#] Kamble A, Sawant S, Singh H. 16S ribosomal RNA gene-based metagenomics: A review. Biomed Res J. 2020 Jun 12;7:5-11. doi: 10.4103/BMRJ.BMRJ_4_20.

[REF3#] Breitwieser FP, Lu J, Salzberg SL. A review of methods and databases for metagenomic classification and assembly. Brief Bioinform. 2019 Jul 19;20(4):1125-1136. doi: 10.1093/bib/bbx120. PMID: 29028872; PMCID: PMC6781581.

[REF4#] Bharti R, Grimm DG. Current challenges and best-practice protocols for microbiome analysis. Brief Bioinform. 2021 Jan 18;22(1):178-193. doi: 10.1093/bib/bbz155. PMID: 31848574; PMCID: PMC7820839.

[REF5#] Blaxter M, Mann J, Chapman T, et al. Defining operational taxonomic units using DNA barcode data. Philos Trans R Soc Lond B Biol Sci. Oct 29 2005;360(1462):1935-43. doi:10.1098/rstb.2005.1725.

[REF6#] Callahan BJ, McMurdie PJ, Holmes SP. Exact sequence variants should replace operational taxonomic units in marker-gene data analysis. The ISME Journal. 2017/12/01 2017;11(12):2639-2643. doi:10.1038/ismej.2017.119

[REF7#] Manandhar I, Alimadadi A, Aryal S, Munroe PB, Joe B, Cheng X. Gut microbiome-based supervised machine learning for clinical diagnosis of inflammatory bowel diseases. Am J Physiol Gastrointest Liver Physiol. 2021 Mar 1;320(3):G328-G337. doi: 10.1152/ajpgi.00360.2020. Epub 2021 Jan 13. PMID: 33439104; PMCID: PMC8828266.

[REF8#] Wang X, Xiao Y, Xu X, Guo L, Yu Y, Li N, Xu C. Characteristics of Fecal Microbiota and Machine Learning Strategy for Fecal Invasive Biomarkers in Pediatric Inflammatory Bowel Disease. Front Cell Infect Microbiol. 2021 Dec 7;11:711884. doi: 10.3389/fcimb.2021.711884. PMID: 34950604; PMCID: PMC8688824.

[REF9#] Thomas AM, Manghi P, Asnicar F, Pasolli E, Armanini F, Zolfo M, Beghini F, Manara S, Karcher N, Pozzi C, Gandini S, Serrano D, Tarallo S, Francavilla A, Gallo G, Trompetto M, Ferrero G, Mizutani S, Shiroma H, Shiba S, Shibata T, Yachida S, Yamada T, Wirbel J, Schrotz-King P, Ulrich CM, Brenner H, Arumugam M, Bork P, Zeller G, Cordero F, Dias-Neto E, Setubal JC, Tett A, Pardini B, Rescigno M, Waldron L, Naccarati A, Segata N. Metagenomic analysis of colorectal cancer datasets identifies cross-cohort microbial diagnostic signatures and a link with choline degradation. Nat Med. 2019 Apr;25(4):667-678.

doi: 10.1038/s41591-019-0405-7. Epub 2019 Apr 1. Erratum in: Nat Med. 2019 Dec;25(12):1948. PMID: 30936548.

[REF10#] Gao Y, Zhu Z, Sun F. Increasing prediction performance of colorectal cancer disease status using random forests classification based on metagenomic shotgun sequencing data. Synth Syst Biotechnol. 2022 Jan 27;7(1):574-585. doi: 10.1016/j.synbio.2022.01.005. PMID: 35155839; PMCID: PMC8801753.

[REF11#] Aryal S, Alimadadi A, Manandhar I, Joe B, Cheng X. Machine Learning Strategy for Gut Microbiome-Based Diagnostic Screening of Cardiovascular Disease. Hypertension. 2020 Nov;76(5):1555-1562. doi: 10.1161/HYPERTENSIONAHA.120.15885. Epub 2020 Sep 10. PMID: 32909848; PMCID: PMC7577586.

[REF12#] Marcos-Zambrano LJ, Karaduzovic-Hadziabdic K, Loncar Turukalo T, Przymus P, Trajkovik V, Aasmets O, Berland M, Gruca A, Hasic J, Hron K, Klammsteiner T, Kolev M, Lahti L, Lopes MB, Moreno V, Naskinova I, Org E, Paciência I, Papoutsoglou G, Shigdel R, Stres B, Vilne B, Yousef M, Zdravevski E, Tsamardinos I, Carrillo de Santa Pau E, Claesson MJ, Moreno-Indias I, Truu J. Applications of Machine Learning in Human Microbiome Studies: A Review on Feature Selection, Biomarker Identification, Disease Prediction and Treatment. Front Microbiol. 2021 Feb 19;12:634511. doi: 10.3389/fmicb.2021.634511. PMID: 33737920; PMCID: PMC7962872.

[REF13#] Human Microbiome Project Consortium. Structure, function and diversity of the healthy human microbiome. Nature. 2012 Jun 13;486(7402):207-14. doi: 10.1038/nature11234. PMID: 22699609; PMCID: PMC3564958.

[REF14#] Thompson LR, Sanders JG, McDonald D, Amir A, Ladau J, Locey KJ, Prill RJ, Tripathi A, Gibbons SM, Ackermann G, Navas-Molina JA, Janssen S, Kopylova E, Vázquez-Baeza Y, González A, Morton JT, Mirarab S, Zech Xu Z, Jiang L, Haroon MF, Kanbar J, Zhu Q, Jin Song S, Kosciolek T, Bokulich NA, Lefler J, Brislawn CJ, Humphrey G, Owens SM,

Hampton-Marcell J, Berg-Lyons D, McKenzie V, Fierer N, Fuhrman JA, Clauset A, Stevens RL, Shade A, Pollard KS, Goodwin KD, Jansson JK, Gilbert JA, Knight R; Earth Microbiome Project Consortium. A communal catalogue reveals Earth's multiscale microbial diversity. Nature. 2017 Nov 23;551(7681):457-463. doi: 10.1038/nature24621. Epub 2017 Nov 1. PMID: 29088705; PMCID: PMC6192678.

[REF15#] Integrative HMP (iHMP) Research Network Consortium. The Integrative Human Microbiome Project. Nature. 2019 May;569(7758):641-648. doi: 10.1038/s41586-019-1238-8. Epub 2019 May 29. PMID: 31142853; PMCID: PMC6784865.

[REF16#] Lloyd-Price J, Arze C, Ananthakrishnan AN, Schirmer M, Avila-Pacheco J, Poon TW, Andrews E, Ajami NJ, Bonham KS, Brislawn CJ, Casero D, Courtney H, Gonzalez A, Graeber TG, Hall AB, Lake K, Landers CJ, Mallick H, Plichta DR, Prasad M, Rahnavard G, Sauk J, Shungin D, Vázquez-Baeza Y, White RA 3rd; IBDMDB Investigators, Braun J, Denson LA, Jansson JK, Knight R, Kugathasan S, McGovern DPB, Petrosino JF, Stappenbeck TS, Winter HS, Clish CB, Franzosa EA, Vlamakis H, Xavier RJ, Huttenhower C. Multi-omics of the gut microbial ecosystem in inflammatory bowel diseases. Nature. 2019 May;569(7758):655-662. doi: 10.1038/s41586-019-1237-9. Epub 2019 May 29. PMID: 31142855; PMCID: PMC6650278.

[REF17#] Hornung BVH, Zwittink RD, Kuijper EJ. Issues and current standards of controls in microbiome research. FEMS Microbiol Ecol. 2019 May 1;95(5):fiz045. doi: 10.1093/femsec/fiz045. PMID: 30997495; PMCID: PMC6469980.

[REF18#] Cernava T, Rybakova D, Buscot F, Clavel T, McHardy AC, Meyer F, Meyer F, Overmann J, Stecher B, Sessitsch A, Schloter M, Berg G; MicrobiomeSupport Team. Metadata harmonization-Standards are the key for a better usage of omics data for integrative microbiome analysis. Environ Microbiome. 2022 Jun 24;17(1):33. doi: 10.1186/s40793-022-00425-1. PMID: 35751093; PMCID: PMC9233336.

[REF19#] Duvallet C, Gibbons SM, Gurry T, Irizarry RA, Alm EJ. Meta-analysis of gut microbiome studies identifies disease-specific and shared responses. Nat Commun. 2017 Dec 5;8(1):1784. doi: 10.1038/s41467-017-01973-8. PMID: 29209090; PMCID: PMC5716994.

[REF20#] Gloor GB, Macklaim JM, Pawlowsky-Glahn V, Egozcue JJ. Microbiome Datasets Are Compositional: And This Is Not Optional. Front Microbiol. 2017 Nov 15;8:2224. doi: 10.3389/fmicb.2017.02224. PMID: 29187837; PMCID: PMC5695134.

[REF21#] Guyon I, Weston J, Barnhill S, Vapnik V. Gene Selection for Cancer Classification using Support Vector Machines. Machine Learning. 2002;46(1/3):389–422. doi: 10.1023/A:1012487302797.

[REF22#] Sanavia T, Aiolli F, Da San Martino G, Bisognin A, Di Camillo B. Improving biomarker list stability by integration of biological knowledge in the learning process. BMC Bioinformatics. 2012 Mar 28;13 Suppl 4(Suppl 4):S22. doi: 10.1186/1471-2105-13-S4-S22. PMID: 22536969; PMCID: PMC3314566.

[REF23#] Wan Xiang Shen, Shu Ran Liang, Yu Yang Jiang, Yu Zong Chen, Enhanced metagenomic deep learning for disease prediction and consistent signature recognition by restructured microbiome 2D representations, Patterns, Volume 4, Issue 1, 2023, 100658, ISSN 2666-3899, https://doi.org/10.1016/j.patter.2022.100658.

[REF24#] Wan Xiang Shen and others, AggMapNet: enhanced and explainable low-sample omics deep learning with feature-aggregated multi-channel networks, Nucleic Acids Research, Volume 50, Issue 8, 6 May 2022, Page e45, https://doi.org/10.1093/nar/gkac010

[REF25#] Ma S., Zhang Z. OmicsMapNet: transforming omics data to take advantage of deep convolutional neural network for discovery. 2018; arXiv doi:23 may 2019, preprint: not peer reviewedhttps://arxiv.org/abs/1804.05283.

[REF26#] Bazgir O., Zhang R., Dhruba S.R., Rahman R., Ghosh S., Pal R. Representation of features as images with neighborhood dependencies for compatibility with convolutional neural networks. Nat. Commun. 2020; 11:4391.

[REF27#] McInnes et al., (2018). UMAP: Uniform Manifold Approximation and Projection. Journal of Open Source Software, 3(29), 861, https://doi.org/10.21105/joss.00861

[REF28#] Lundberg, Scott M., and Su-In Lee. "A unified approach to interpreting model predictions." Advances in Neural Information Processing Systems (2017)

[REF29#] Gou W, Ling CW, He Y, Jiang Z, Fu Y, Xu F, Miao Z, Sun TY, Lin JS, Zhu HL, Zhou H, Chen YM, Zheng JS. Interpretable Machine Learning Framework Reveals Robust Gut Microbiome Features Associated With Type 2 Diabetes. Diabetes Care. 2021 Feb;44(2):358-366. doi: 10.2337/dc20-1536. Epub 2020 Dec 7. PMID: 33288652; PMCID: PMC7818326.

[REF30#] Gan RW, Sun D, Tatro AR, Cohen-Mekelburg S, Wiitala WL, Zhu J, Waljee AK. Replicating prediction algorithms for hospitalization and corticosteroid use in patients with inflammatory bowel disease. PLoS One. 2021 Sep 20;16(9):e0257520. doi: 10.1371/journal.pone.0257520. PMID: 34543353; PMCID: PMC8452029.

[REF31#] Flores GE, Caporaso JG, Henley JB, Rideout JR, Domogala D, Chase J, Leff JW, Vázquez-Baeza Y, Gonzalez A, Knight R, Dunn RR, Fierer N. Temporal variability is a personalized feature of the human microbiome. Genome Biol. 2014 Dec 3;15(12):531. doi: 10.1186/s13059-014-0531-y. PMID: 25517225; PMCID: PMC4252997.

[REF32#] Halfvarson J, Brislawn CJ, Lamendella R, Vázquez-Baeza Y, Walters WA, Bramer LM, D'Amato M, Bonfiglio F, McDonald D, Gonzalez A, McClure EE, Dunklebarger MF, Knight R, Jansson JK. Dynamics of the human gut microbiome in inflammatory bowel disease. Nat Microbiol. 2017 Feb 13;2:17004. doi: 10.1038/nmicrobiol.2017.4. PMID: 28191884; PMCID: PMC5319707.

[REF33#] McDonald D, Hyde E, Debelius JW, Morton JT, Gonzalez A, Ackermann G, Aksenov AA, Behsaz B, Brennan C, Chen Y, DeRight Goldasich L, Dorrestein PC, Dunn RR, Fahimipour AK, Gaffney J, Gilbert JA, Gogul G, Green JL, Hugenholtz P, Humphrey G, Huttenhower C, Jackson MA, Janssen S, Jeste DV, Jiang L, Kelley ST, Knights D, Kosciolek T, Ladau J, Leach J, Marotz C, Meleshko D, Melnik AV, Metcalf JL, Mohimani H, Montassier E, Navas-Molina J, Nguyen TT, Peddada S, Pevzner P, Pollard KS, Rahnavard G, Robbins-Pianka A, Sangwan N, Shorenstein J, Smarr L, Song SJ, Spector T, Swafford AD, Thackray VG, Thompson LR, Tripathi A, Vázquez-Baeza Y, Vrbanac A, Wischmeyer P, Wolfe E, Zhu Q; American Gut Consortium, Knight R. American Gut: an Open Platform for Citizen Science Microbiome Research. mSystems. 2018 May 15;3(3):e00031-18. doi: 10.1128/mSystems.00031-18. PMID: 29795809; PMCID: PMC5954204.

[REF34#] Caporaso JG, Kuczynski J, Stombaugh J, Bittinger K, Bushman FD, Costello EK, Fierer N, Peña AG, Goodrich JK, Gordon JI, Huttley GA, Kelley ST, Knights D, Koenig JE, Ley RE, Lozupone CA, McDonald D, Muegge BD, Pirrung M, Reeder J, Sevinsky JR, Turnbaugh PJ, Walters WA, Widmann J, Yatsunenko T, Zaneveld J, Knight R. QIIME allows analysis of high-throughput community sequencing data. Nat Methods. 2010 May;7(5):335-6. doi: 10.1038/nmeth.f.303. Epub 2010 Apr 11. PMID: 20383131; PMCID: PMC3156573.

[REF35#] Lin H, Peddada SD. Analysis of microbial compositions: a review of normalization and differential abundance analysis. NPJ Biofilms Microbiomes. 2020 Dec 2;6(1):60. doi: 10.1038/s41522-020-00160-w. PMID: 33268781; PMCID: PMC7710733.

[REF36#] Lloréns-Rico V, Vieira-Silva S, Gonçalves PJ, Falony G, Raes J. Benchmarking microbiome transformations favors experimental quantitative approaches to address compositionality and sampling depth biases. Nat Commun. 2021 Jun 11;12(1):3562. doi: 10.1038/s41467-021-23821-6. PMID: 34117246; PMCID: PMC8196019.

[REF37#] Baruzzo G, Patuzzi I, Di Camillo B. Beware to ignore the rare: how imputing zero-values can improve the quality of 16S rRNA gene studies results. BMC Bioinformatics. 2022 Feb 7;22(Suppl 15):618. doi: 10.1186/s12859-022-04587-0. PMID: 35130833; PMCID: PMC8822630.

[REF38#] Pasolli E, Truong DT, Malik F, Waldron L, Segata N. Machine Learning Meta-analysis of Large Metagenomic Datasets: Tools and Biological Insights. PLoS Comput Biol. 2016 Jul 11;12(7):e1004977. doi: 10.1371/journal.pcbi.1004977. PMID: 27400279; PMCID: PMC4939962.

[REF39#] Kubinski R, Djamen-Kepaou JY, Zhanabaev T, Hernandez-Garcia A, Bauer S, Hildebrand F, Korcsmaros T, Karam S, Jantchou P, Kafi K, Martin RD. Benchmark of Data Processing Methods and Machine Learning Models for Gut Microbiome-Based Diagnosis of Inflammatory Bowel Disease. Front Genet. 2022 Feb 14;13:784397. doi: 10.3389/fgene.2022.784397. PMID: 35251123; PMCID: PMC8895431.

[REF40#] Lahti, Leo, Sudarshan Shetty, Felix M Ernst, et al. 2021. Orchestrating Microbiome Analysis with Bioconductor [Beta Version]. microbiome.github.io/oma/.

[REF41#] Sanz, H., Valim, C., Vegas, E. et al. SVM-RFE: selection and visualization of the most relevant features through non-linear kernels. BMC Bioinformatics 19, 432 (2018). https://doi.org/10.1186/s12859-018-2451-4

[REF42#] Lin X, Li C, Zhang Y, Su B, Fan M, Wei H. Selecting Feature Subsets Based on SVM-RFE and the Overlapping Ratio with Applications in Bioinformatics. Molecules. 2017 Dec 26;23(1):52. doi: 10.3390/molecules23010052. PMID: 29278382; PMCID: PMC5943966

[REF43#] Racedo S, Portnoy I, Vélez JI, San-Juan-Vergara H, Sanjuan M, Zurek E. A new pipeline for structural characterization and classification of RNA-Seq microbiome data. BioData Min.

2021 Jul 9;14(1):31. doi: 10.1186/s13040-021-00266-7. PMID: 34243809; PMCID: PMC8268467.

[REF44#] Chicco, D., Jurman, G. The advantages of the Matthews correlation coefficient (MCC) over F1 score and accuracy in binary classification evaluation. BMC Genomics 21, 6 (2020). https://doi.org/10.1186/s12864-019-6413-7

[REF45#] Freedman, D., Pisani, R., & Purves, R. (2007). Statistics (international student edition). Pisani, R. Purves, 4th Edn. WW Norton & Company, New York.

[REF46#] Bray, J. R. and J. T. Curtis. 1957. An ordination of upland forest communities of southern Wisconsin. Ecological Monographs 27:325-349.

[REF47#] Mohana CP, Perumal K. A Survey on Feature Selection Stability Measures. International Journal of Computer Science and Information Technology. 2016 Jan; 5(1): 2279–0764.

[REF48#] Khaire UM, Dhanalakshmi R. Stability of Feature Selection Algorithm: A Review. Journal of King Saud University - Computer and Information Sciences. 2022;34(4):1060–73. doi: 10.1016/j.jksuci.2019.06.012

[REF49#] Scikit-learn: Machine Learning in Python, Pedregosa et al., JMLR 12, pp. 2825-2830, 2011.

[REF50#] Effect of size and heterogeneity of samples on biomarker discovery: synthetic and real data assessment. Barbara Di Camillo, Tiziana Sanavia, Matteo Martini, Giuseppe Jurman, Francesco Sambo, Annalisa Barla, Margherita Squillario, Cesare Furlanello, Gianna Toffolo, Claudio Cobelli. PLoS One . 2012;7(3):e32200. doi: 10.1371/journal.pone.0032200.

[REF51#] West RM. Best practice in statistics: The use of log transformation. Ann Clin Biochem. 2022 May;59(3):162-165. doi: 10.1177/00045632211050531. Epub 2021 Oct 19. PMID: 34666549; PMCID: PMC9036143.

[REF52#] Giliberti R, Cavaliere S, Mauriello IE, Ercolini D, Pasolli E. Host phenotype classification from human microbiome data is mainly driven by the presence of microbial taxa. PLoS

Comput Biol. 2022 Apr 21;18(4):e1010066. doi: 10.1371/journal.pcbi.1010066. PMID: 35446845; PMCID: PMC9064115.

[REF53#] Bakir-Gungor B, Hacılar H, Jabeer A, Nalbantoglu OU, Aran O, Yousef M. Inflammatory bowel disease biomarkers of human gut microbiota selected via different feature selection methods. PeerJ. 2022 Apr 25;10:e13205. doi: 10.7717/peerj.13205. PMID: 35497193; PMCID: PMC9048649.

[REF54#] Liñares-Blanco J, Fernandez-Lozano C, Seoane JA, López-Campos G. Machine Learning Based Microbiome Signature to Predict Inflammatory Bowel Disease Subtypes. Front Microbiol. 2022 May 17;13:872671. doi: 10.3389/fmicb.2022.872671. PMID: 35663898; PMCID: PMC9157387.

[REF55#] Lee Y; Cappellato M; Camillo BD: Supporting data for "Machine learning based feature selection to search stable microbial biomarkers: application to inflammatory bowel disease" GigaScience Database. 2023. http://dx.doi.org/10.5524/102450
