# Supplementary material for: Machine learning–based feature selection to search stable microbial biomarkers: application to inflammatory bowel disease
Source: Gigascience. 2023 Oct 26;12:giad083. doi: 10.1093/gigascience/giad083 (PMC10600917; doi:10.1093/gigascience/giad083)
Supplement: giad083_Supplemental_File [file giad083_supplemental_file.docx]

**Supplementary Material**

Machine learning based feature selection to search stable microbial biomarkers: application to inflammatory bowel disease

***Tables and Figures are arranged according to the mention in the Main Article.**


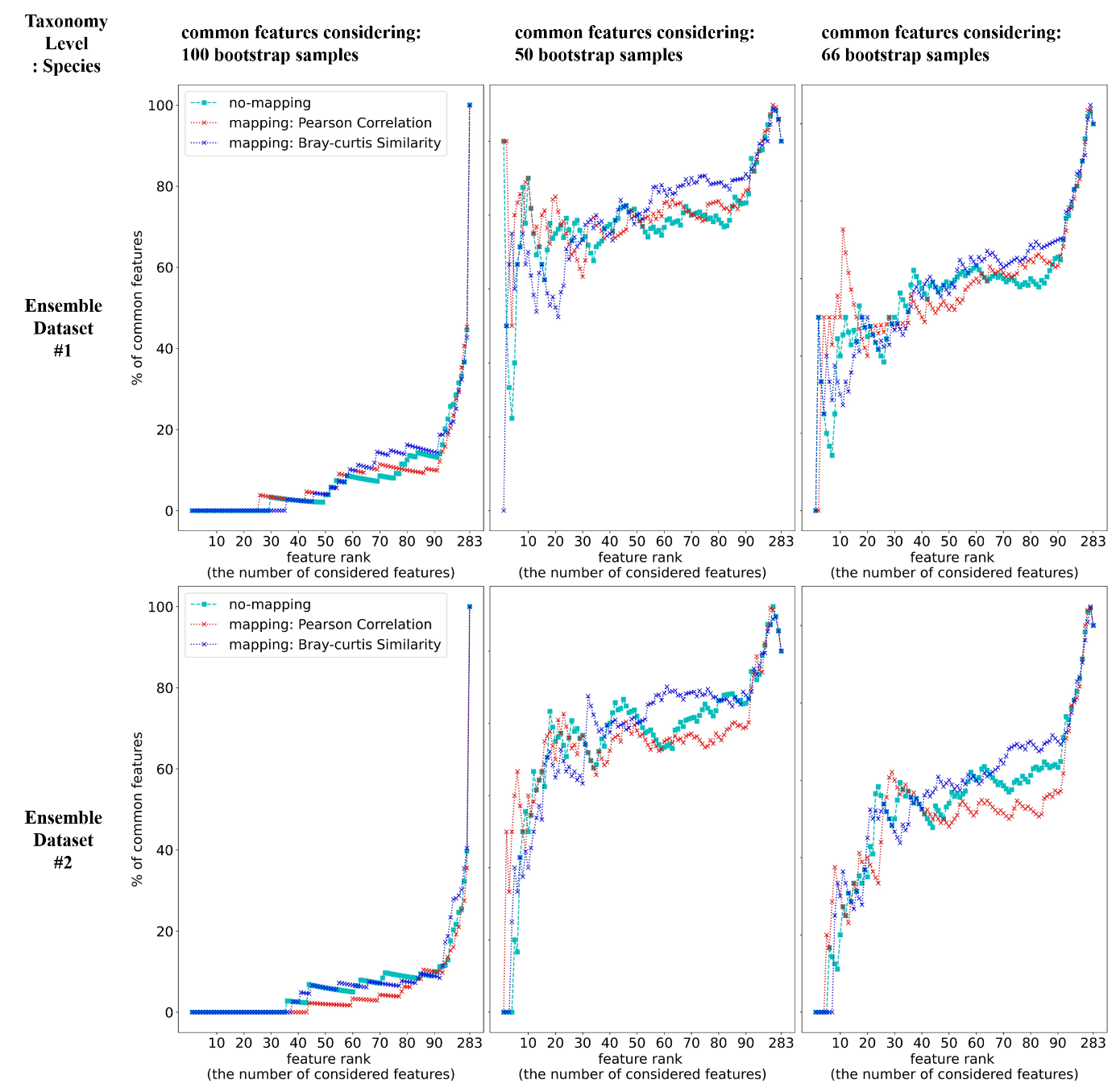


**Supplementary Figure 1. Number of common features across all bootstrap samples using RFE to rank features (species level).** X-axis represents feature rank (i.e. the number of considered features); y-axis represents the percentage of common features considering 100 (left), at least 50 (middle), or 66 (right) bootstrap samples.


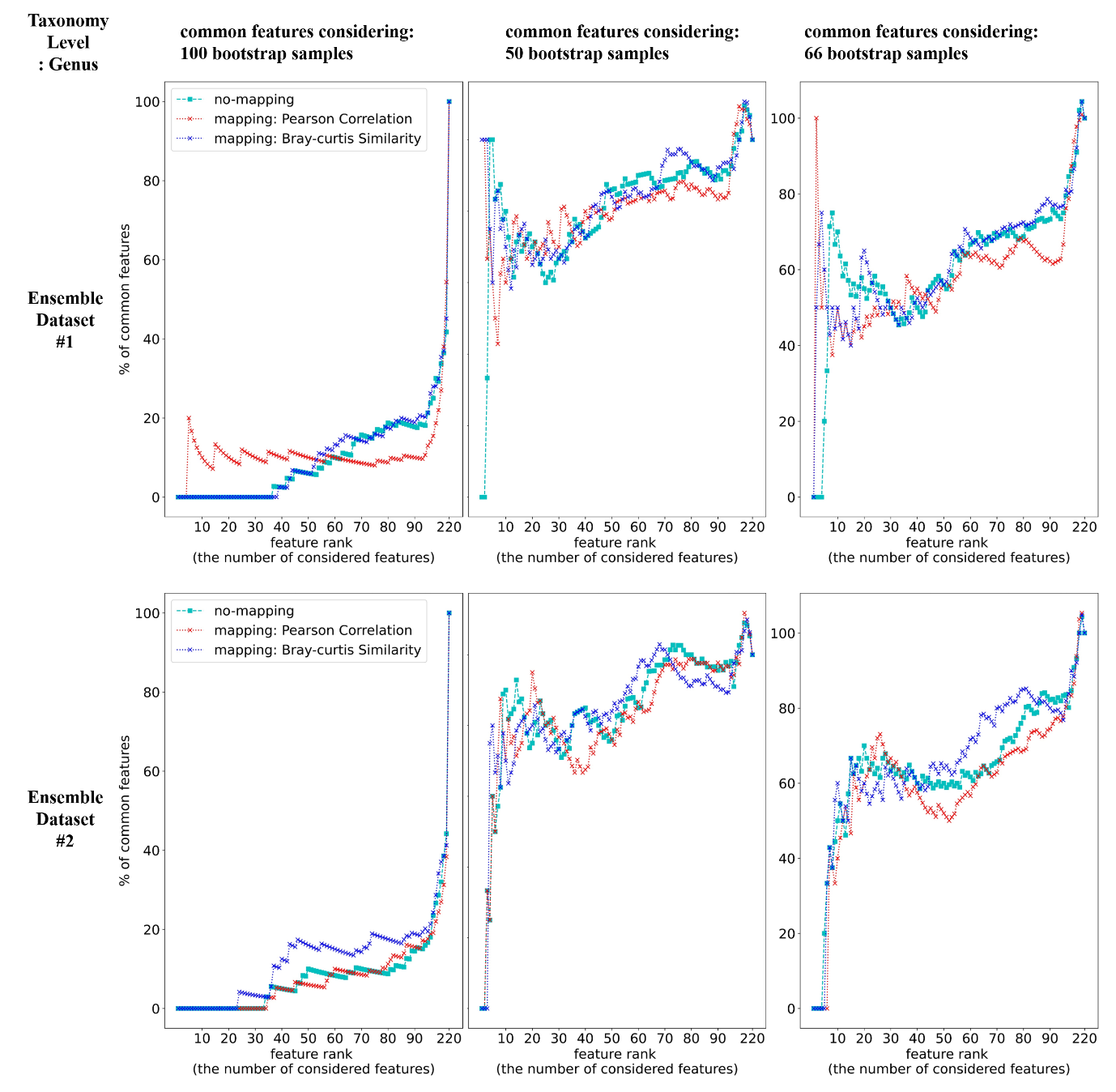


**Supplementary Figure 2. Number of common features across all bootstrap samples using RFE to rank features (genus level).** X-axis represents feature rank (i.e. the number of considered features); y-axis represents the percentage of common features considering 100 (left), at least 50 (middle), or 66 (right) bootstrap samples.

| species-ED1, RFE with  no mapping | | | | | | | |
| --- | --- | --- | --- | --- | --- | --- | --- |
| Optimal Feature #: 251 | AUC | Sensitivity | Specificity | PPV | NPV | Accuracy | MCC |
| Logistic Regression | 0.974 | 0.943 | 0.966 | 0.957 | 0.955 | 0.955 | 0.910 |
| Linear SVM | 0.954 | 0.943 | 0.966 | 0.957 | 0.955 | 0.955 | 0.910 |
| Random Forest | 0.935 | 0.954 | 0.915 | 0.900 | 0.961 | 0.932 | 0.865 |
| XGBoost | 0.972 | 0.954 | 0.878 | 0.863 | 0.960 | 0.912 | 0.828 |
| MLP-Perceptron | 0.937 | 0.920 | 0.954 | 0.942 | 0.937 | 0.939 | 0.876 |
| MLP-1 hidden layer | 0.992 | 0.957 | 0.989 | 0.985 | 0.966 | 0.975 | 0.949 |
| MLP-2 hidden layer | 0.992 | 0.957 | 0.984 | 0.980 | 0.966 | 0.972 | 0.943 |
| MLP-3 hidden layer | 0.991 | 0.940 | 0.979 | 0.973 | 0.953 | 0.962 | 0.923 |
| species-ED1, RFE with  mapping: Pearson Correlation | | | | | | | |
| Optimal Feature #: 251 | AUC | Sensitivity | Specificity | PPV | NPV | Accuracy | MCC |
| Logistic Regression | 0.974 | 0.929 | 0.920 | 0.903 | 0.941 | 0.924 | 0.846 |
| Linear SVM | 0.917 | 0.914 | 0.920 | 0.901 | 0.930 | 0.917 | 0.833 |
| Random Forest | 0.917 | 0.943 | 0.892 | 0.876 | 0.951 | 0.915 | 0.831 |
| XGBoost | 0.971 | 0.954 | 0.878 | 0.863 | 0.960 | 0.912 | 0.828 |
| MLP-Perceptron | 0.917 | 0.903 | 0.931 | 0.916 | 0.923 | 0.918 | 0.836 |
| MLP-1 hidden layer | 0.985 | 0.960 | 0.961 | 0.952 | 0.968 | 0.961 | 0.920 |
| MLP-2 hidden layer | 0.981 | 0.954 | 0.963 | 0.954 | 0.963 | 0.959 | 0.918 |
| MLP-3 hidden layer | 0.979 | 0.951 | 0.966 | 0.957 | 0.961 | 0.959 | 0.918 |
| species-ED1, RFE with  mapping: Bray-Curtis Similarity | | | | | | | |
| Optimal Feature #: 283 | AUC | Sensitivity | Specificity | PPV | NPV | Accuracy | MCC |
| Logistic Regression | 0.973 | 0.943 | 0.966 | 0.957 | 0.955 | 0.955 | 0.910 |
| Linear SVM | 0.954 | 0.943 | 0.966 | 0.957 | 0.955 | 0.955 | 0.910 |
| Random Forest | 0.934 | 0.957 | 0.910 | 0.896 | 0.964 | 0.931 | 0.863 |
| XGBoost | 0.965 | 0.957 | 0.871 | 0.857 | 0.962 | 0.910 | 0.824 |
| MLP-Perceptron | 0.930 | 0.931 | 0.929 | 0.917 | 0.945 | 0.930 | 0.861 |
| MLP-1 hidden layer | 0.991 | 0.957 | 0.989 | 0.985 | 0.966 | 0.975 | 0.949 |
| MLP-2 hidden layer | 0.991 | 0.966 | 0.984 | 0.980 | 0.973 | 0.976 | 0.951 |
| MLP-3 hidden layer | 0.993 | 0.960 | 0.986 | 0.982 | 0.968 | 0.975 | 0.949 |
| species-ED2, RFE with  no mapping | | | | | | | |
| Optimal Feature #: 251 | AUC | Sensitivity | Specificity | PPV | NPV | Accuracy | MCC |
| Logistic Regression | 0.964 | 0.914 | 0.920 | 0.901 | 0.930 | 0.917 | 0.833 |
| Linear SVM | 0.911 | 0.914 | 0.908 | 0.889 | 0.929 | 0.911 | 0.820 |
| Random Forest | 0.881 | 0.880 | 0.883 | 0.858 | 0.901 | 0.882 | 0.761 |
| XGBoost | 0.942 | 0.874 | 0.883 | 0.857 | 0.897 | 0.879 | 0.756 |
| MLP-Perceptron | 0.888 | 0.889 | 0.887 | 0.870 | 0.914 | 0.888 | 0.780 |
| MLP-1 hidden layer | 0.968 | 0.903 | 0.915 | 0.895 | 0.921 | 0.910 | 0.817 |
| MLP-2 hidden layer | 0.967 | 0.909 | 0.920 | 0.901 | 0.926 | 0.915 | 0.827 |
| MLP-3 hidden layer | 0.961 | 0.920 | 0.933 | 0.917 | 0.936 | 0.927 | 0.853 |
| species-ED2, RFE with  mapping: Pearson Correlation | | | | | | | |
| Optimal Feature # : 283 | AUC | Sensitivity | Specificity | PPV | NPV | Accuracy | MCC |
| Logistic Regression | 0.985 | 0.986 | 0.908 | 0.896 | 0.988 | 0.943 | 0.889 |
| Linear SVM | 0.913 | 0.929 | 0.897 | 0.878 | 0.940 | 0.911 | 0.822 |
| Random Forest | 0.911 | 0.891 | 0.931 | 0.912 | 0.914 | 0.913 | 0.824 |
| XGBoost | 0.955 | 0.894 | 0.885 | 0.862 | 0.912 | 0.889 | 0.777 |
| MLP-Perceptron | 0.938 | 0.966 | 0.910 | 0.897 | 0.971 | 0.935 | 0.872 |
| MLP-1 hidden layer | 0.983 | 0.997 | 0.949 | 0.941 | 0.998 | 0.971 | 0.942 |
| MLP-2 hidden layer | 0.985 | 0.980 | 0.947 | 0.937 | 0.983 | 0.962 | 0.924 |
| MLP-3 hidden layer | 0.983 | 0.980 | 0.961 | 0.953 | 0.984 | 0.969 | 0.939 |
| species-ED2, RFE with  mapping: Bray-Curtis Similarity | | | | | | | |
| Optimal Feature #: 267 | AUC | Sensitivity | Specificity | PPV | NPV | Accuracy | MCC |
| Logistic Regression | 0.980 | 0.929 | 0.920 | 0.903 | 0.941 | 0.924 | 0.846 |
| Linear SVM | 0.930 | 0.929 | 0.931 | 0.915 | 0.942 | 0.930 | 0.858 |
| Random Forest | 0.916 | 0.903 | 0.929 | 0.911 | 0.922 | 0.917 | 0.832 |
| XGBoost | 0.955 | 0.897 | 0.885 | 0.863 | 0.914 | 0.890 | 0.780 |
| MLP-Perceptron | 0.941 | 0.969 | 0.913 | 0.900 | 0.973 | 0.938 | 0.877 |
| MLP-1 hidden layer | 0.984 | 0.986 | 0.954 | 0.945 | 0.988 | 0.968 | 0.937 |
| MLP-2 hidden layer | 0.975 | 0.931 | 0.940 | 0.926 | 0.945 | 0.936 | 0.871 |
| MLP-3 hidden layer | 0.988 | 0.963 | 0.940 | 0.928 | 0.969 | 0.950 | 0.900 |

**Supplementary Table 1. Prediction performance of models trained at species level and tested on the independent test.** Features are selected by Linear SVM based Recursive Feature Elimination**.**

| genus-ED1, RFE with  no mapping | | | | | | | |
| --- | --- | --- | --- | --- | --- | --- | --- |
| Optimal Feature # from RFE: 192 | AUC | Sensitivity | Specificity | PPV | NPV | Accuracy | MCC |
| Logistic Regression | 0.964 | 0.943 | 0.931 | 0.917 | 0.953 | 0.936 | 0.872 |
| Linear SVM | 0.943 | 0.943 | 0.943 | 0.930 | 0.953 | 0.943 | 0.884 |
| Random Forest | 0.926 | 0.946 | 0.906 | 0.890 | 0.954 | 0.924 | 0.848 |
| XGBoost | 0.972 | 0.960 | 0.869 | 0.855 | 0.964 | 0.910 | 0.824 |
| MLP-Perceptron | 0.932 | 0.931 | 0.933 | 0.919 | 0.944 | 0.932 | 0.864 |
| MLP-1 hidden layer | 0.989 | 0.943 | 0.986 | 0.982 | 0.955 | 0.967 | 0.933 |
| MLP-2 hidden layer | 0.992 | 0.943 | 0.986 | 0.982 | 0.956 | 0.967 | 0.933 |
| MLP-3 hidden layer | 0.989 | 0.943 | 0.984 | 0.979 | 0.955 | 0.966 | 0.931 |
| genus-ED1, RFE with  mapping: Pearson Correlation | | | | | | | |
| Optimal Feature # from RFE: 22 | AUC | Sensitivity | Specificity | PPV | NPV | Accuracy | MCC |
| Logistic Regression | 0.890 | 0.900 | 0.782 | 0.768 | 0.907 | 0.834 | 0.678 |
| Linear SVM | 0.834 | 0.886 | 0.782 | 0.765 | 0.895 | 0.828 | 0.664 |
| Random Forest | 0.875 | 0.917 | 0.832 | 0.815 | 0.926 | 0.870 | 0.745 |
| XGBoost | 0.900 | 0.900 | 0.784 | 0.770 | 0.907 | 0.836 | 0.680 |
| MLP-Perceptron | 0.761 | 0.803 | 0.720 | 0.714 | 0.840 | 0.757 | 0.538 |
| MLP-1 hidden layer | 0.925 | 0.911 | 0.807 | 0.792 | 0.919 | 0.854 | 0.714 |
| MLP-2 hidden layer | 0.919 | 0.891 | 0.862 | 0.840 | 0.909 | 0.875 | 0.751 |
| MLP-3 hidden layer | 0.926 | 0.914 | 0.844 | 0.825 | 0.924 | 0.875 | 0.754 |
| genus-ED1, RFE with  mapping: Bray-Curtis Similarity | | | | | | | |
| Optimal Feature # from RFE: 220 | AUC | Sensitivity | Specificity | PPV | NPV | Accuracy | MCC |
| Logistic Regression | 0.964 | 0.943 | 0.943 | 0.930 | 0.953 | 0.943 | 0.884 |
| Linear SVM | 0.943 | 0.943 | 0.943 | 0.930 | 0.953 | 0.943 | 0.884 |
| Random Forest | 0.928 | 0.949 | 0.908 | 0.893 | 0.957 | 0.926 | 0.853 |
| XGBoost | 0.975 | 0.960 | 0.857 | 0.844 | 0.964 | 0.903 | 0.813 |
| MLP-Perceptron | 0.937 | 0.906 | 0.968 | 0.958 | 0.929 | 0.940 | 0.880 |
| MLP-1 hidden layer | 0.993 | 0.940 | 0.986 | 0.982 | 0.953 | 0.966 | 0.931 |
| MLP-2 hidden layer | 0.990 | 0.954 | 0.984 | 0.980 | 0.964 | 0.971 | 0.941 |
| MLP-3 hidden layer | 0.992 | 0.957 | 0.989 | 0.985 | 0.966 | 0.975 | 0.949 |
| genus-ED2, RFE with  no mapping | | | | | | | |
| Optimal Feature # from RFE: 220 | AUC | Sensitivity | Specificity | PPV | NPV | Accuracy | MCC |
| Logistic Regression | 0.972 | 0.900 | 0.920 | 0.900 | 0.920 | 0.911 | 0.820 |
| Linear SVM | 0.910 | 0.900 | 0.920 | 0.900 | 0.920 | 0.911 | 0.820 |
| Random Forest | 0.912 | 0.897 | 0.926 | 0.908 | 0.918 | 0.913 | 0.825 |
| XGBoost | 0.952 | 0.886 | 0.894 | 0.871 | 0.907 | 0.890 | 0.779 |
| MLP-Perceptron | 0.926 | 0.920 | 0.931 | 0.918 | 0.938 | 0.926 | 0.854 |
| MLP-1 hidden layer | 0.985 | 0.974 | 0.956 | 0.947 | 0.979 | 0.964 | 0.928 |
| MLP-2 hidden layer | 0.972 | 0.903 | 0.920 | 0.900 | 0.922 | 0.912 | 0.822 |
| MLP-3 hidden layer | 0.970 | 0.900 | 0.920 | 0.900 | 0.920 | 0.911 | 0.820 |
| genus-ED2, RFE with  mapping: Pearson Correlation | | | | | | | |
| Optimal Feature # from RFE: 192 | AUC | Sensitivity | Specificity | PPV | NPV | Accuracy | MCC |
| Logistic Regression | 0.984 | 0.943 | 0.897 | 0.880 | 0.951 | 0.917 | 0.835 |
| Linear SVM | 0.911 | 0.914 | 0.908 | 0.889 | 0.929 | 0.911 | 0.820 |
| Random Forest | 0.912 | 0.897 | 0.926 | 0.908 | 0.918 | 0.913 | 0.825 |
| XGBoost | 0.956 | 0.889 | 0.885 | 0.861 | 0.908 | 0.887 | 0.772 |
| MLP-Perceptron | 0.927 | 0.946 | 0.908 | 0.896 | 0.956 | 0.925 | 0.853 |
| MLP-1 hidden layer | 0.987 | 0.957 | 0.956 | 0.946 | 0.965 | 0.957 | 0.913 |
| MLP-2 hidden layer | 0.985 | 0.966 | 0.959 | 0.950 | 0.972 | 0.962 | 0.923 |
| MLP-3 hidden layer | 0.988 | 0.974 | 0.956 | 0.947 | 0.979 | 0.964 | 0.928 |
| genus-ED2, RFE with  mapping: Bray-Curtis Similarity | | | | | | | |
| Optimal Feature # from RFE: 206 | AUC | Sensitivity | Specificity | PPV | NPV | Accuracy | MCC |
| Logistic Regression | 0.983 | 0.943 | 0.897 | 0.880 | 0.951 | 0.917 | 0.835 |
| Linear SVM | 0.920 | 0.943 | 0.897 | 0.880 | 0.951 | 0.917 | 0.835 |
| Random Forest | 0.909 | 0.891 | 0.926 | 0.907 | 0.914 | 0.911 | 0.819 |
| XGBoost | 0.962 | 0.889 | 0.874 | 0.850 | 0.907 | 0.880 | 0.759 |
| MLP-Perceptron | 0.899 | 0.834 | 0.963 | 0.950 | 0.883 | 0.906 | 0.815 |
| MLP-1 hidden layer | 0.982 | 0.929 | 0.961 | 0.950 | 0.944 | 0.946 | 0.892 |
| MLP-2 hidden layer | 0.985 | 0.946 | 0.954 | 0.943 | 0.956 | 0.950 | 0.900 |
| MLP-3 hidden layer | 0.979 | 0.963 | 0.940 | 0.928 | 0.969 | 0.950 | 0.900 |

**Supplementary Table 2. Prediction performance of models trained at genus level and tested on the independent test.** Features are selected by Linear SVM based Recursive Feature Elimination**.**

| species-ED1, RFE with  no mapping | | | | | | | |
| --- | --- | --- | --- | --- | --- | --- | --- |
| Optimal Feature # from RFE: 251 | AUC | Sensitivity | Specificity | PPV | NPV | Accuracy | MCC |
| Logistic Regression | 0.988 | 0.923 | 0.956 | 0.945 | 0.939 | 0.941 | 0.881 |
| Linear SVM | 0.941 | 0.926 | 0.956 | 0.945 | 0.941 | 0.943 | 0.884 |
| Random Forest | 0.946 | 0.949 | 0.943 | 0.931 | 0.958 | 0.945 | 0.890 |
| XGBoost | 0.977 | 0.938 | 0.921 | 0.906 | 0.949 | 0.929 | 0.857 |
| MLP-Perceptron | 0.928 | 0.899 | 0.957 | 0.945 | 0.922 | 0.931 | 0.861 |
| MLP-1 hidden layer | 0.990 | 0.942 | 0.980 | 0.975 | 0.954 | 0.963 | 0.925 |
| MLP-2 hidden layer | 0.990 | 0.952 | 0.977 | 0.971 | 0.962 | 0.966 | 0.931 |
| MLP-3 hidden layer | 0.991 | 0.943 | 0.982 | 0.978 | 0.955 | 0.965 | 0.929 |
| species-ED1, RFE with  mapping: Pearson Correlation | | | | | | | |
| Optimal Feature # from RFE: 251 | AUC | Sensitivity | Specificity | PPV | NPV | Accuracy | MCC |
| Logistic Regression | 0.983 | 0.952 | 0.940 | 0.928 | 0.960 | 0.945 | 0.890 |
| Linear SVM | 0.934 | 0.940 | 0.929 | 0.914 | 0.950 | 0.934 | 0.867 |
| Random Forest | 0.947 | 0.950 | 0.944 | 0.932 | 0.959 | 0.947 | 0.893 |
| XGBoost | 0.974 | 0.935 | 0.916 | 0.900 | 0.946 | 0.924 | 0.848 |
| MLP-Perceptron | 0.930 | 0.936 | 0.923 | 0.909 | 0.948 | 0.929 | 0.858 |
| MLP-1 hidden layer | 0.988 | 0.958 | 0.965 | 0.957 | 0.966 | 0.962 | 0.924 |
| MLP-2 hidden layer | 0.985 | 0.935 | 0.963 | 0.954 | 0.948 | 0.951 | 0.900 |
| MLP-3 hidden layer | 0.983 | 0.944 | 0.958 | 0.948 | 0.955 | 0.952 | 0.902 |
| species-ED1, RFE with  mapping: Bray-Curtis Similarity | | | | | | | |
| Optimal Feature # from RFE: 283 | AUC | Sensitivity | Specificity | PPV | NPV | Accuracy | MCC |
| Logistic Regression | 0.988 | 0.923 | 0.956 | 0.945 | 0.939 | 0.941 | 0.881 |
| Linear SVM | 0.941 | 0.926 | 0.956 | 0.945 | 0.941 | 0.943 | 0.884 |
| Random Forest | 0.945 | 0.945 | 0.946 | 0.934 | 0.955 | 0.945 | 0.889 |
| XGBoost | 0.974 | 0.930 | 0.916 | 0.900 | 0.942 | 0.923 | 0.844 |
| MLP-Perceptron | 0.930 | 0.931 | 0.929 | 0.918 | 0.945 | 0.930 | 0.861 |
| MLP-1 hidden layer | 0.986 | 0.937 | 0.983 | 0.979 | 0.951 | 0.963 | 0.925 |
| MLP-2 hidden layer | 0.990 | 0.948 | 0.974 | 0.968 | 0.959 | 0.963 | 0.924 |
| MLP-3 hidden layer | 0.988 | 0.938 | 0.980 | 0.975 | 0.951 | 0.961 | 0.922 |
| species-ED2, RFE with  no mapping | | | | | | | |
| Optimal Feature # from RFE: 251 | AUC | Sensitivity | Specificity | PPV | NPV | Accuracy | MCC |
| Logistic Regression | 0.973 | 0.949 | 0.917 | 0.902 | 0.957 | 0.931 | 0.862 |
| Linear SVM | 0.937 | 0.952 | 0.921 | 0.908 | 0.959 | 0.935 | 0.870 |
| Random Forest | 0.927 | 0.943 | 0.911 | 0.896 | 0.952 | 0.925 | 0.851 |
| XGBoost | 0.953 | 0.921 | 0.907 | 0.889 | 0.934 | 0.913 | 0.826 |
| MLP-Perceptron | 0.909 | 0.931 | 0.886 | 0.875 | 0.945 | 0.906 | 0.818 |
| MLP-1 hidden layer | 0.984 | 0.954 | 0.927 | 0.913 | 0.961 | 0.939 | 0.877 |
| MLP-2 hidden layer | 0.981 | 0.946 | 0.928 | 0.914 | 0.955 | 0.936 | 0.871 |
| MLP-3 hidden layer | 0.965 | 0.954 | 0.929 | 0.916 | 0.962 | 0.940 | 0.880 |
| species-ED2, RFE with  mapping: Pearson Correlation | | | | | | | |
| Optimal Feature # from RFE: 283 | AUC | Sensitivity | Specificity | PPV | NPV | Accuracy | MCC |
| Logistic Regression | 0.986 | 0.960 | 0.949 | 0.939 | 0.967 | 0.954 | 0.908 |
| Linear SVM | 0.943 | 0.954 | 0.931 | 0.918 | 0.962 | 0.941 | 0.882 |
| Random Forest | 0.937 | 0.950 | 0.924 | 0.910 | 0.958 | 0.935 | 0.871 |
| XGBoost | 0.964 | 0.929 | 0.913 | 0.897 | 0.941 | 0.920 | 0.840 |
| MLP-Perceptron | 0.941 | 0.943 | 0.939 | 0.926 | 0.953 | 0.941 | 0.880 |
| MLP-1 hidden layer | 0.990 | 0.964 | 0.961 | 0.952 | 0.971 | 0.962 | 0.924 |
| MLP-2 hidden layer | 0.990 | 0.966 | 0.967 | 0.960 | 0.973 | 0.967 | 0.933 |
| MLP-3 hidden layer | 0.990 | 0.966 | 0.964 | 0.956 | 0.972 | 0.965 | 0.928 |
| species-ED2, RFE with  mapping: Bray-Curtis Similarity | | | | | | | |
| Optimal Feature # from RFE: 267 | AUC | Sensitivity | Specificity | PPV | NPV | Accuracy | MCC |
| Logistic Regression | 0.979 | 0.960 | 0.940 | 0.928 | 0.967 | 0.949 | 0.898 |
| Linear SVM | 0.947 | 0.960 | 0.933 | 0.921 | 0.967 | 0.945 | 0.890 |
| Random Forest | 0.937 | 0.950 | 0.924 | 0.910 | 0.958 | 0.936 | 0.871 |
| XGBoost | 0.966 | 0.926 | 0.917 | 0.900 | 0.939 | 0.921 | 0.841 |
| MLP-Perceptron | 0.946 | 0.961 | 0.932 | 0.921 | 0.967 | 0.945 | 0.890 |
| MLP-1 hidden layer | 0.990 | 0.969 | 0.962 | 0.954 | 0.975 | 0.965 | 0.930 |
| MLP-2 hidden layer | 0.981 | 0.940 | 0.952 | 0.941 | 0.951 | 0.947 | 0.892 |
| MLP-3 hidden layer | 0.988 | 0.968 | 0.960 | 0.952 | 0.973 | 0.964 | 0.926 |

**Supplementary Table 3. Prediction performance of models trained at species level and tested on the external test.** Features are selected by Linear SVM based Recursive Feature Elimination**.** ED1,2 refers that a model built based on a training dataset in ED1,2 and tested by ED2,1.

| genus-ED1, RFE with  no mapping | | | | | | | |
| --- | --- | --- | --- | --- | --- | --- | --- |
| Optimal Feature # from RFE: 192 | AUC | Sensitivity | Specificity | PPV | NPV | Accuracy | MCC |
| Logistic Regression | 0.980 | 0.923 | 0.926 | 0.910 | 0.937 | 0.925 | 0.848 |
| Linear SVM | 0.928 | 0.932 | 0.924 | 0.908 | 0.944 | 0.927 | 0.854 |
| Random Forest | 0.940 | 0.947 | 0.933 | 0.920 | 0.956 | 0.939 | 0.878 |
| XGBoost | 0.975 | 0.940 | 0.914 | 0.899 | 0.949 | 0.926 | 0.851 |
| MLP-Perceptron | 0.915 | 0.917 | 0.913 | 0.896 | 0.932 | 0.915 | 0.829 |
| MLP-1 hidden layer | 0.989 | 0.934 | 0.974 | 0.966 | 0.948 | 0.956 | 0.911 |
| MLP-2 hidden layer | 0.988 | 0.931 | 0.979 | 0.973 | 0.946 | 0.957 | 0.914 |
| MLP-3 hidden layer | 0.988 | 0.929 | 0.976 | 0.969 | 0.944 | 0.955 | 0.909 |
| genus-ED1, RFE with  mapping: Pearson Correlation | | | | | | | |
| Optimal Feature # from RFE: 22 | AUC | Sensitivity | Specificity | PPV | NPV | Accuracy | MCC |
| Logistic Regression | 0.918 | 0.920 | 0.816 | 0.801 | 0.927 | 0.862 | 0.732 |
| Linear SVM | 0.871 | 0.917 | 0.825 | 0.809 | 0.925 | 0.866 | 0.738 |
| Random Forest | 0.906 | 0.929 | 0.882 | 0.864 | 0.939 | 0.903 | 0.807 |
| XGBoost | 0.942 | 0.924 | 0.835 | 0.819 | 0.931 | 0.874 | 0.754 |
| MLP-Perceptron | 0.785 | 0.810 | 0.759 | 0.750 | 0.854 | 0.782 | 0.586 |
| MLP-1 hidden layer | 0.954 | 0.915 | 0.868 | 0.849 | 0.926 | 0.889 | 0.779 |
| MLP-2 hidden layer | 0.935 | 0.880 | 0.901 | 0.878 | 0.903 | 0.892 | 0.781 |
| MLP-3 hidden layer | 0.950 | 0.904 | 0.893 | 0.872 | 0.920 | 0.898 | 0.794 |
| genus-ED1, RFE with  mapping: Bray-Curtis Similarity | | | | | | | |
| Optimal Feature # from RFE: 220 | AUC | Sensitivity | Specificity | PPV | NPV | Accuracy | MCC |
| Logistic Regression | 0.981 | 0.923 | 0.935 | 0.920 | 0.938 | 0.930 | 0.858 |
| Linear SVM | 0.929 | 0.929 | 0.929 | 0.913 | 0.942 | 0.929 | 0.856 |
| Random Forest | 0.941 | 0.946 | 0.935 | 0.922 | 0.955 | 0.940 | 0.879 |
| XGBoost | 0.974 | 0.944 | 0.902 | 0.886 | 0.952 | 0.921 | 0.842 |
| MLP-Perceptron | 0.916 | 0.876 | 0.956 | 0.943 | 0.907 | 0.920 | 0.841 |
| MLP-1 hidden layer | 0.989 | 0.936 | 0.979 | 0.973 | 0.950 | 0.960 | 0.919 |
| MLP-2 hidden layer | 0.988 | 0.935 | 0.970 | 0.961 | 0.949 | 0.954 | 0.907 |
| MLP-3 hidden layer | 0.988 | 0.934 | 0.974 | 0.967 | 0.948 | 0.956 | 0.912 |
| genus-ED2, RFE with  no mapping | | | | | | | |
| Optimal Feature # from RFE: 220 | AUC | Sensitivity | Specificity | PPV | NPV | Accuracy | MCC |
| Logistic Regression | 0.974 | 0.949 | 0.938 | 0.925 | 0.958 | 0.943 | 0.884 |
| Linear SVM | 0.940 | 0.949 | 0.931 | 0.917 | 0.957 | 0.939 | 0.877 |
| Random Forest | 0.935 | 0.948 | 0.921 | 0.907 | 0.956 | 0.933 | 0.866 |
| XGBoost | 0.963 | 0.923 | 0.912 | 0.895 | 0.936 | 0.917 | 0.833 |
| MLP-Perceptron | 0.911 | 0.896 | 0.927 | 0.911 | 0.919 | 0.913 | 0.826 |
| MLP-1 hidden layer | 0.989 | 0.962 | 0.961 | 0.953 | 0.969 | 0.962 | 0.923 |
| MLP-2 hidden layer | 0.976 | 0.942 | 0.940 | 0.927 | 0.952 | 0.941 | 0.881 |
| MLP-3 hidden layer | 0.972 | 0.943 | 0.940 | 0.928 | 0.953 | 0.942 | 0.882 |
| genus-ED2, RFE with  mapping: Pearson Correlation | | | | | | | |
| Optimal Feature # from RFE: 192 | AUC | Sensitivity | Specificity | PPV | NPV | Accuracy | MCC |
| Logistic Regression | 0.977 | 0.952 | 0.928 | 0.915 | 0.959 | 0.939 | 0.877 |
| Linear SVM | 0.934 | 0.946 | 0.921 | 0.907 | 0.955 | 0.932 | 0.864 |
| Random Forest | 0.934 | 0.946 | 0.922 | 0.908 | 0.955 | 0.933 | 0.865 |
| XGBoost | 0.958 | 0.934 | 0.899 | 0.882 | 0.944 | 0.915 | 0.829 |
| MLP-Perceptron | 0.907 | 0.915 | 0.900 | 0.886 | 0.932 | 0.906 | 0.816 |
| MLP-1 hidden layer | 0.988 | 0.953 | 0.966 | 0.958 | 0.962 | 0.960 | 0.920 |
| MLP-2 hidden layer | 0.989 | 0.957 | 0.965 | 0.957 | 0.965 | 0.961 | 0.922 |
| MLP-3 hidden layer | 0.987 | 0.957 | 0.962 | 0.953 | 0.965 | 0.960 | 0.919 |
| genus-ED2, RFE with  mapping: Bray-Curtis Similarity | | | | | | | |
| Optimal Feature # from RFE: 206 | AUC | Sensitivity | Specificity | PPV | NPV | Accuracy | MCC |
| Logistic Regression | 0.977 | 0.932 | 0.928 | 0.913 | 0.944 | 0.930 | 0.859 |
| Linear SVM | 0.923 | 0.923 | 0.924 | 0.908 | 0.937 | 0.923 | 0.846 |
| Random Forest | 0.928 | 0.929 | 0.927 | 0.911 | 0.941 | 0.928 | 0.854 |
| XGBoost | 0.954 | 0.926 | 0.897 | 0.879 | 0.937 | 0.910 | 0.820 |
| MLP-Perceptron | 0.887 | 0.818 | 0.955 | 0.938 | 0.870 | 0.894 | 0.790 |
| MLP-1 hidden layer | 0.986 | 0.942 | 0.965 | 0.957 | 0.954 | 0.955 | 0.909 |
| MLP-2 hidden layer | 0.985 | 0.948 | 0.957 | 0.947 | 0.958 | 0.953 | 0.905 |
| MLP-3 hidden layer | 0.975 | 0.942 | 0.939 | 0.926 | 0.953 | 0.940 | 0.880 |

**Supplementary Table 4. Prediction performance of models trained at genus level and tested on the external test.** Features are selected by Linear SVM based Recursive Feature Elimination**.** ED1,2 refers that a model built based on a training dataset in ED1,2 and tested by ED2,1. # 1,2 and tested by dataset # 2,1.

| Level: species | | |
| --- | --- | --- |
| **Models developed on ED1** | MCC from best performing algorithm applied on Test1 | MCC from best performing algorithm applied on External Set ED2 |
| no-mapping | 0.949  (MLP-1 hidden layer) | 0.931  (MLP-2 hidden layer) |
| mapping:  Pearson Correlation | 0.92  (MLP-1 hidden layer) | 0.924  (MLP-1 hidden layer) |
| mapping:  Bray-Curtis Similarity | 0.951  (MLP-2 hidden layer) | 0.925  (MLP-1 hidden layer) |
| Models developed on ED2 | MCC from best performing algorithm applied on Test2 | MCC from best performing algorithm applied on External Set ED1 |
| no-mapping | 0.853  (MLP-2 hidden layer) | 0.88  (MLP-2 hidden layer) |
| mapping:  Pearson Correlation | 0.942  (MLP-1 hidden layer) | 0.933  (MLP-2 hidden layer) |
| mapping:  Bray-Curtis Similarity | 0.937  (MLP-1 hidden layer) | 0.93  (MLP-1 hidden layer) |
| Level: genus | | |
| **Models developed on** ED1 | MCC from best performing algorithm applied on Test1 | MCC from best performing algorithm applied on External Set ED2 |
| no-mapping | 0.933  (MLP-2 hidden layer) | 0.914  (MLP-2 hidden layer) |
| mapping:  Pearson Correlation | 0.754  (MLP-2 hidden layer) | 0.807  (Random Forest) |
| mapping:  Bray-Curtis Similarity | 0.949  (MLP-2 hidden layer) | 0.919  (MLP-1 hidden layer) |
| Models developed on ED2 | MCC from best performing algorithm applied on Test2 | MCC from best performing algorithm applied on External Set ED1 |
| no-mapping | 0.928  (MLP-1 hidden layer) | 0.923  (MLP-1 hidden layer) |
| mapping: Pearson Correlation | 0.928  (MLP-2 hidden layer) | 0.922  (MLP-2 hidden layer) |
| mapping: Bray-Curtis Similarity | 0.9  (MLP-2 hidden layer) | 0.909  (MLP-1 hidden layer) |

**Supplementary Table 5.** MCC obtained using the best-performing algorithm (RFE with Bray-Curtis-Similarity based mapping) using the set of optimal features.

| Level: species | | |
| --- | --- | --- |
| **Models developed on ED1** | MCC from best performing algorithm applied on Test1 | MCC from best performing algorithm applied on External Set ED2 |
| no-mapping | 0.814  (Random Forest) | 0.825  (XGBoost) |
| mapping:  Pearson Correlation | 0.832  (Random Forest) | 0.832  (Random Forest) |
| mapping:  Bray-Curtis Similarity | 0.8  (XGBoost) | 0.836  (Random Forest) |
| Models developed on ED2 | MCC from best performing algorithm applied on Test2 | MCC from best performing algorithm applied on External Set ED1 |
| no-mapping | 0.829  (Logistic Regression) | 0.816  (Random Forest) |
| mapping:  Pearson Correlation | 0.775  (MLP-1 hidden layer) | 0.816  (Random Forest) |
| mapping:  Bray-Curtis Similarity | 0.829  (Logistic Regression) | 0.816  (Random Forest) |
| Level: genus | | |
| **Models developed on** ED1 | MCC from best performing algorithm applied on Test1 | MCC from best performing algorithm applied on External Set ED2 |
| no-mapping | 0.832  (Random Forest) | 0.821  (Random Forest) |
| mapping:  Pearson Correlation | 0.576  (Random Forest) | 0.535  (Random Forest) |
| mapping:  Bray-Curtis Similarity | 0.816  (Random Forest) | 0.826  (Random Forest) |
| Models developed on ED2 | MCC from best performing algorithm applied on Test2 | MCC from best performing algorithm applied on External Set ED1 |
| no-mapping | 0.803  (MLP-2 hidden layer) | 0.808  (Random Forest) |
| mapping: Pearson Correlation | 0.81  (Linear SVM) | 0.801  (Random Forest) |
| mapping: Bray-Curtis Similarity | 0.801  (MLP-2 hidden layer) | 0.811  (MLP-2 hidden layer) |

**Supplementary Table 6.** MCC obtained using the best performing algorithm (RFE with Bray Curtis-Similarity based mapping) and the set of top 14 features.

| species-Entire Dataset, RFE with  mapping: Bray-Curtis Similarity | | | | | | | |
| --- | --- | --- | --- | --- | --- | --- | --- |
| Optimal Feature # from RFE: 267 | AUC | Sensitivity | Specificity | PPV | NPV | Accuracy | MCC |
| Logistic Regression | 0.986 | 0.986 | 0.960 | 0.952 | 0.988 | 0.971 | 0.943 |
| Linear SVM | 0.970 | 0.986 | 0.954 | 0.945 | 0.988 | 0.968 | 0.937 |
| Random Forest | 0.928 | 0.929 | 0.926 | 0.910 | 0.942 | 0.927 | 0.854 |
| XGBoost | 0.974 | 0.933 | 0.900 | 0.882 | 0.943 | 0.915 | 0.829 |
| MLP-Perceptron | 0.955 | 0.953 | 0.956 | 0.946 | 0.962 | 0.955 | 0.909 |
| MLP-1 hidden layer | 0.989 | 0.989 | 0.976 | 0.971 | 0.991 | 0.982 | 0.963 |
| MLP-2 hidden layer | 0.991 | 0.979 | 0.979 | 0.974 | 0.983 | 0.979 | 0.958 |
| MLP-3 hidden layer | 0.989 | 0.983 | 0.975 | 0.969 | 0.986 | 0.978 | 0.956 |

**Supplementary Table 7. Prediction performance of models trained at species level on the entire dataset (ED1+ED2) and tested on the independent test. Optimal set of features** are selected by Linear SVM based Recursive Feature Elimination with mapping using Bray-Curtis Similarity. Training dataset is the merge of training dataset in species-ED1 and species-ED2. Test dataset is the merge of test dataset in species-ED1 and species- ED2

| species-Entire Dataset, RFE with  mapping: Bray-Curtis Similarity | | | | | | | |
| --- | --- | --- | --- | --- | --- | --- | --- |
| Feature# from RFE: 14 | AUC | Sensitivity | Specificity | PPV | NPV | Accuracy | MCC |
| Logistic Regression | 0.953 | 0.914 | 0.885 | 0.865 | 0.928 | 0.898 | 0.796 |
| Linear SVM | 0.900 | 0.914 | 0.885 | 0.865 | 0.928 | 0.898 | 0.796 |
| Random Forest | 0.924 | 0.933 | 0.915 | 0.898 | 0.944 | 0.923 | 0.845 |
| XGBoost | 0.962 | 0.931 | 0.892 | 0.874 | 0.942 | 0.910 | 0.820 |
| MLP-Perceptron | 0.850 | 0.801 | 0.899 | 0.874 | 0.865 | 0.855 | 0.718 |
| MLP-1 hidden layer | 0.963 | 0.927 | 0.902 | 0.884 | 0.939 | 0.913 | 0.826 |
| MLP-2 hidden layer | 0.963 | 0.933 | 0.905 | 0.887 | 0.944 | 0.917 | 0.834 |
| MLP-3 hidden layer | 0.967 | 0.934 | 0.893 | 0.876 | 0.944 | 0.911 | 0.824 |

**Supplementary Table 8. Prediction performance of models trained at species level on the entire dataset (ED1+ED2) and tested on the independent test. Top 14 features** are selected by Linear SVM based Recursive Feature Elimination with mapping using Bray-Curtis Similarity. Training dataset is the merge of training dataset in species-ED1 and species-ED2. Test dataset is the merge of test dataset in species-ED1 and species- ED2


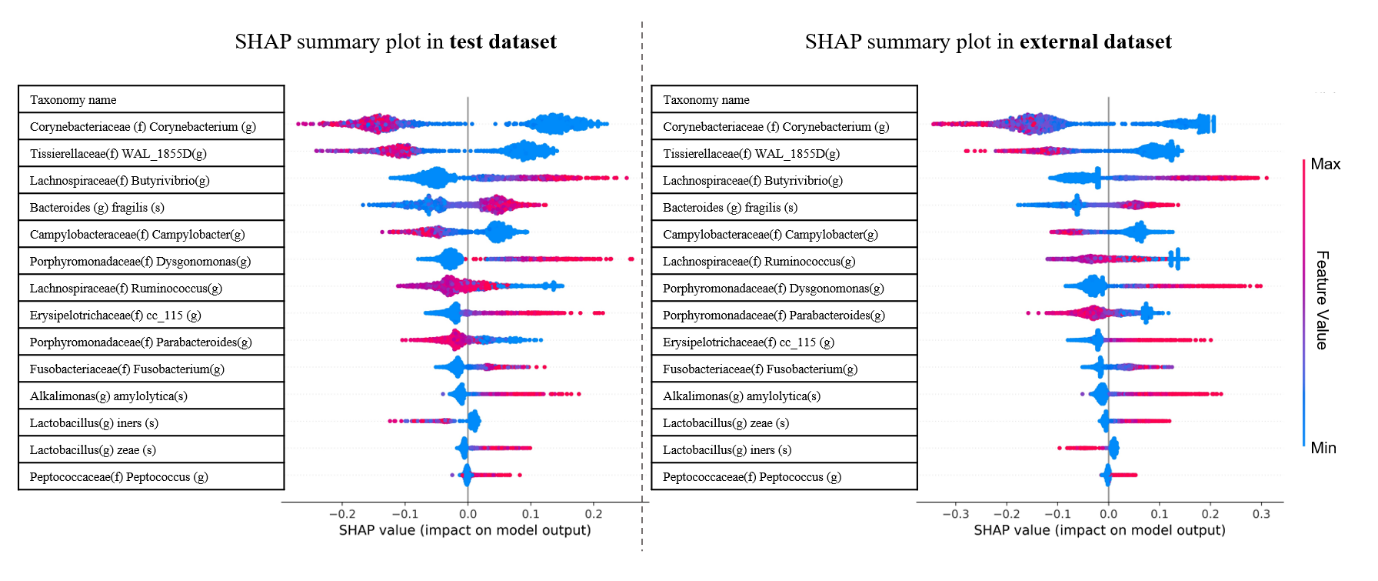


**Supplementary Figure 3. SHAP summary plot of top ten features (by the Random Forest model in Supplementary Table 6). SHAP values from a) test dataset b) external set(dataset #4 in Table 1) . Feature is listed along to its rank.**

|  | Ensemble Dataset#1 | | | Ensemble Dataset#2 | | |
| --- | --- | --- | --- | --- | --- | --- |
| Source of data | Total | Training Dataset | Test Dataset | Total | Training Dataset | Test Dataset |
| Dataset #1 | 46 | 35 | 11 | 50 | 45 | 5 |
| Dataset #2 | 323 | 258 | 65 | 314 | 249 | 65 |
| Dataset #3 | 415 | 334 | 81 | 421 | 334 | 87 |

**Supplementary Table 9. Number of samples by data source**

| **Level: species** | | |
| --- | --- | --- |
| **Models developed on ED1** | **MCC from best performing algorithm applied on Test1** | **MCC from best performing algorithm applied on External Set ED2** |
| mapping:  Bray-Curtis Similarity | 0.951  (MLP-2 hidden layer) | 0.925  (MLP-1 hidden layer) |
| AggMapNet | 0.911 | 0.907 |
| **Models developed on ED2** | **MCC from best performing algorithm applied on Test2** | **MCC from best performing algorithm applied on External Set ED1** |
| mapping:  Bray-Curtis Similarity | 0.937  (MLP-1 hidden layer) | 0.93  (MLP-1 hidden layer) |
| AggMapNet | 0.897 | 0.907 |
| Level: genus | | |
| Models developed on ED1 | MCC from best performing algorithm applied on Test1 | MCC from best performing algorithm applied on External Set ED2 |
| mapping:  Bray-Curtis Similarity | 0.949  (MLP-2 hidden layer) | 0.919  (MLP-1 hidden layer) |
| AggMapNet | 0.885 | 0.889 |
| Models developed on ED2 | MCC from best performing algorithm applied on Test2 | MCC from best performing algorithm applied on External Set ED1 |
| mapping:  Bray-Curtis Similarity | 0.9  (MLP-2 hidden layer) | 0.909  (MLP-1 hidden layer) |
| AggMapNet | 0.845 | 0.897 |

**Supplementary Table 10. Comparison of final MCC performance between our pipeline (mapping: Bray-Curtis Similarity in Supplementary Table 5) and AggMapNet (REF57#)**

| q-value | Fold change | average±std  in IBD | average±std  in non-IBD | Taxonomy name |
| --- | --- | --- | --- | --- |
| <0.001 | 0.033 | 1.019±13.906 | 30.518±104.629 | Corynebacteriaceae(f) Corynebacterium (g) |
| <0.001 | 0.025 | 0.76±11.079 | 29.939±108.275 | Tissierellaceae(f) WAL_1855D(g) |
| <0.001 | 22.215 | 0.386±1.63 | 0.017±0.101 | Lachnospiraceae(f) Butyrivibrio(g) |
| <0.001 | 11.824 | 274.207±1678.29 | 23.191±86.331 | Bacteroides (g) fragilis (s) |
| <0.001 | 0.072 | 0.937±17.699 | 13.083±57.889 | Campylobacteraceae(f) Campylobacter(g) |
| <0.001 | 25.399 | 0.905±20.592 | 0.036±0.4 | Porphyromonadaceae(f) Dysgonomonas(g) |
| <0.001 | 3.444 | 37.205±112.275 | 10.803±22.717 | Lachnospiraceae(f) Ruminococcus(g) |
| <0.001 | 16.432 | 2.432±11.881 | 0.148±1.196 | Erysipelotrichaceae(f) cc_115 (g) |
| 0.323 | 3.142 | 70.34±210.632 | 22.385±44.001 | Porphyromonadaceae(f) Parabacteroides(g) |
| <0.001 | 53.433 | 108.441±716.35 | 2.029±16.553 | Fusobacteriaceae(f) Fusobacterium(g) |
| <0.001 | 65.189 | 0.8±4.483 | 0.012±0.111 | Alkalimonas(g) amylolytica(s) |
| <0.001 | 0.0035 | 0.022±0.42 | 6.299±38.999 | Lactobacillus(g) iners (s) |
| <0.001 | 4.228 | 0.683±4.611 | 0.162±4.304 | Lactobacillus(g) zeae (s) |
| <0.001 | 1.566 | 0.763±4.042 | 0.487±2.152 | Peptococcaceae(f) Peptococcus (g) |

**Supplementary Table 11. Q-value and Fold change of top 14 features.** Q value is calculated by FDR correction of the p-values, and p-values are obtained by t-test. Fold change is obtained by dividing average feature value from non-IBD samples by average from IBD samples.
