# Supplementary material for: Machine learning–based feature selection to search stable microbial biomarkers: application to inflammatory bowel disease
Source: Gigascience. 2023 Oct 26;12:giad083. doi: 10.1093/gigascience/giad083 (PMC10600917; doi:10.1093/gigascience/giad083)
Supplement: giad083_Response_to_Reviewer_Comments_Original_Submission [file giad083_response_to_reviewer_comments_original_submission.pdf]

Author's Response To Reviewer Comments

Dear Editors,
We sincerely appreciate the time and effort invested by the reviewers in critically evaluating our manuscript and providing valuable feedback.
Below, we present a point-by-point response to each of the reviewers' comments and elaborate on the revisions made to address their concerns.
We sincerely hope that our revised manuscript meets the high standards of GigaScience and would be suitable for publication.
Thank you for your time and consideration.
Sincerely,
Barbara Di Camillo
Full Prof. in Computer Science
Information Engineering Department
Via Gradenigo 6B
35131, Padova, Italy
Mail: barbara.dicamillo@unipd.it
Phone: +39 049 827 7671

Reviewer reports:
Reviewer #1: After careful consideration of the revisions made by the authors and their responses to the first round of review, I am pleased to note that they have adequately addressed most of the major issues and improved the manuscript accordingly. However, I would like to highlight a few minor revisions that need to be addressed before considering the manuscript for publication.

Minor Revisions:

The authors have introduced a mapping transformation as an alternative method for stable feature selection in machine learning (ML) models with 1D vectors as inputs. I appreciate the theoretical statements provided by the authors, explaining the motivation behind this approach. However, in order to provide more context and clarity, I recommend that the authors highlight the background of their method in the introduction section. Specifically, the authors are encouraged to mention similar methods such as AggMap (https://github.com/shenwanxiang/bidd-aggmap) in their introduction. AggMap is a method that designed to map the unordered features into 2D feature maps (i.e., the image-like data) for deep learning models such as Convolutional Neural Networks models. However, for the traditional ML methods, their inputs are 1D-vectors instead of 2D feature maps. Therefore, there is a need to map unordered features for conventional ML models. By addressing this complementarity, the authors can underscore the importance of the feature order for traditional machine learning models with 1D vectors as inputs, and how their method fills this gap.

==>
As you suggested we have included a paragraph in the introduction section, here reported for your convenience. Thank you.
It is worth noting that there are similar approaches that utilizes similarity matrix to map similar features into closer space [REF23#][REF24#][REF25#][REF26#]. Among others, AggMapNet utilizes a unique approach for data transformation, converting the original data into multi-channel 2D spatial-correlated images through pairwise correlation distances. This is achieved by employing the manifold learning method called Uniform Manifold Approximation and Projection (UMAP) [REF27#]. Through a preliminary clustering step, various channels are selected based on the pairwise correlation distances among features. Subsequently, the feature maps are fed as input to machine learning models, such as convolutional neural networks, enabling effective classification tasks. However, traditional ML methods require as input 1D-vectors. Our approach can map unordered features as 1D vectors for conventional ML models and is therefore somehow complementary to AggMapNet approach.

Secondly, I suggest that the authors revise Figure 1 to improve its clarity and make it easier for readers to understand. A well-designed figure will enhance the overall presentation of the manuscript.
==>
Thank you for your suggestion.
We have revised Fig.1 that we report also here for the reviewer’s convenience.

Fig. 1. Diagram for the overall experiments. The figure reports the analysis performed on ensemble dataset 1 using ensemble dataset 2 only for testing (black: inputs and outputs; blue: analysis steps; green: results assessment). A symmetric analysis was performed on ensemble dataset 2 using ensemble dataset 1 for testing. The inset represents the details of the mapping transformation procedure.

Additionally, I recommend that the authors include a section titled "Theoretical Advantages of Mapping Transformation" in the Results section. This section should highlight the key ideas and theoretical benefits of their proposed approach, further elucidating the contributions of the paper.

==>
We included the following paragraph in section Methods
2.3.2 Mapping transformation and its theoretical advantages
Feature mapping is a crucial step in machine learning that can significantly impact model performance. Feature mapping involves transforming raw input data into a format suitable for the learning algorithm, enabling the extraction of meaningful patterns and relationships. By converting complex and diverse features into a more structured representation, feature mapping empowers the model to discern relevant information, leading to more accurate predictions. In the context of omics data, due to the abundance of features, the problem of identifying relevant features for the predictive model becomes under-constrained, leading to numerous potential sets of relevant features that could achieve comparable accuracy. To address this, we leveraged supplementary data from an external dataset (dataset 4) to impose additional constraints during feature mapping. In essence, this approach aims to account for strong correlations among certain features, indicating their similar importance for the classification task. As a result, we ensure that these correlated features are equally relevant, enhancing the overall performance of the model.
Information about feature correlation is integrated by mapping data using a kernel transformation that has been shown to possibly alleviate feature instability [REF22#]. Transformation matrix P, is obtained using the equation P=D^(-1) (I+α(S-I)) , where S is the similarity matrix, D is the diagonal matrix whose elements are the sum of the elements in the rows of the matrix I+α(S-I) and α is a tuning parameter. The value of α was decided for each experiment using 5-fold cross-validation within its internal training dataset using a grid of 0.01 and from 0.05 to 1 by step 0.05. In our approach, mapping was used only in the RFE step.

Lastly, the authors should carefully revise the manuscript to ensure it meets the journal's formatting requirements and formats. Adhering to the journal's guidelines will enhance the professionalism and readability of the paper.

==>
We have reviewed the entire manuscript to adhere GIGAScience requirements and format. We have revised following:
1) Double line spacing
2) Include page numbering
3) Do not use page break
4) Redraw all main figures to have image resolution of 300 dpi with tiff format
5) Put table titles above the table
6) Reference numbers were revised in the order in which they are cited in the text

We have referred to this guideline:
https://academic.oup.com/gigascience/pages/instructions_to_authors

Considering these minor revisions, I believe the manuscript will be ready for publication. Therefore, I recommend that the authors address these points and make the necessary revisions. Once these revisions have been implemented, I suggest that the manuscript be reassessed for final acceptance.

Thank you for considering my review.

Reviewer #2: I want to commend the authors on a much-improved manuscript. I fully appreciate that it must have been a tremendous amount of work to rerun everything with the log-transformation and to change the major display items.
I would like to raise two additional points, the first of which is of major importance:

1. I am a bit concerned about the numbers in Table 1. For example, for Dataset #1 (the data from the Lloyd-Price et al. paper), the authors report only a single non-IBD sample. In figure one of Lloyd-Price et al, there are multiple nonIBD samples (https://www.nature.com/articles/s41586-019-1237-9/figures/1). Can the authors explain this disparity? Also, the number of samples Dataset #2 (637) seems to be much higher than the number of subjects in the original study (137, see https://www.nature.com/articles/nmicrobiol20174#Sec1). Did the authors report the number of samples rather than the number of subjects?
Lastly, the study from Flores et al. does not seem to be focussed on IBD at all. Am I missing something here or is there a mixup with the references?

==>
We thank the reviewer for giving us the opportunity to clarify a point which could rightfully cause doubts to readers.

As already described in paragraph 2.1, all datasets ere downloaded from Qiita, an open-source microbial study management platform that allows its users to keep track of multiple studies with numerous ‘omics data while also supporting several analytical pipelines through a 3rd-party plugin system, thus providing databases and compute resources. Qiita’s platform allows for quick reanalysis of the datasets that have been deposited. Due to the type of samples considered in our study (nonIBD, CD and UC) the number of samples considered in our work may differ from the original studies (as in the case of Lloyd-Price et al., i.e. dataset #1).

The reviewer's observation is correct. In fact, the study by Flores et al. it does not focus on IBD. Indeed in their research, Flores G. et al. investigated the temporal dynamics of forehead, gut (feces, i.e. the only data that are used in this work), palm and tongue microbial communities of 85 young adults (college-age) from three different U.S. universities, who self-collected their samples every week for about three months. Bacterial and archaeal communities were characterized using high-throughput sequencing of the V4 region of the 16 rRNA gene. The authors collected demographic, lifestyle and hygiene data at the beginning of the study through a standardized survey, to investigate potential drivers of variability. Also weekly questionnaires were used to track changes in health status, medication use, menstrual cycle, and other changes in routine behaviors.

However there is no mistake in the references. We chose this dataset as it has many non-IBD and IBD samples, according to the metadata collected (from subjects suffering Crohns or Colitis diseases).
We have clarified this point in the main ms. Furthermore, we have modified the manuscript (in particular Table 1) by replacing the term “subjects” with “samples”. Indeed, as noted by the reviewer, the numbers reported refer to samples that may belong to the same subject since the studies considered are longitudinal.

2. I would recommend to order the features in Figure 5 according to their enrichment in IBD vs. controls

(most enriched in IBD on the top, most enriched in controls at the bottom, for example). (optional)

==>
Thank you for your suggestion. We have revised Fig.5 that we report also here for the reviewer’s convenience.

Fig. 5. SHAP summary plot of primary indicators of IBD and non IBD. Random Forest model trained by top fourteen features in RFE (species level, trained by the combination of training sets in ED1 and ED2) are used to calculate SHAP values. SHAP values are measured by training dataset. Total rank is the rank between fourteen features used for training the model..
