# Supplementary material for: Machine learning–based feature selection to search stable microbial biomarkers: application to inflammatory bowel disease
Source: Gigascience. 2023 Oct 26;12:giad083. doi: 10.1093/gigascience/giad083 (PMC10600917; doi:10.1093/gigascience/giad083)
Supplement: giad083_Response_to_Reviewer_Comments_Revision_1 [file giad083_response_to_reviewer_comments_revision_1.pdf]

Author's Response To Reviewer Comments

Dear Editor,

We sincerely appreciate the time and effort you have invested in evaluating our manuscript.

We have made the revisions mentioned in your letter and manuscript file, which include:

- Moving the URL to the reference list.
- Revising the 'Availability of Source Code and Requirements' section while including the RRID number.
- Adding a 'Data Availability' section and citing the GigaDB DOI reference.

If there is anything we have missed, please do let us know.

We sincerely hope that our revised manuscript now meets the high standards of GigaScience and is suitable for publication.

Thank you for your time and consideration.

Sincerely,
Barbara Di Camillo

Full Prof. in Computer Science
Information Engineering Department
Via Gradenigo 6B
35131, Padova, Italy
Mail: barbara.dicamillo@unipd.it
Phone: +39 049 827 7671
