# Supplementary material for: Machine learning–based feature selection to search stable microbial biomarkers: application to inflammatory bowel disease
Source: Gigascience. 2023 Oct 26;12:giad083. doi: 10.1093/gigascience/giad083 (PMC10600917; doi:10.1093/gigascience/giad083)
Supplement: giad083_Reviewer_1_Report_Original_Submission — Wan Xiang Shen -- 7/10/2023 Reviewed [file giad083_reviewer_1_report_original_submission.pdf]

Reviewer Report

Title: Machine learning based feature selection to search stable microbial biomarkers: application to inflammatory bowel disease

Version: Original Submission **Date:** 7/10/2023

Reviewer name: Wan Xiang Shen

Reviewer Comments to Author:

After careful consideration of the revisions made by the authors and their responses to the first round of review, I am pleased to note that they have adequately addressed most of the major issues and improved the manuscript accordingly. However, I would like to highlight a few minor revisions that need to be addressed before considering the manuscript for publication.
Minor Revisions:
The authors have introduced a mapping transformation as an alternative method for stable feature selection in machine learning (ML) models with 1D vectors as inputs. I appreciate the theoretical statements provided by the authors, explaining the motivation behind this approach. However, in order to provide more context and clarity, I recommend that the authors highlight the background of their method in the introduction section. Specifically, the authors are encouraged to mention similar methods such as AggMap (https://github.com/shenwanxiang/bidd-aggmap) in their introduction. AggMap is a method that designed to map the unordered features into 2D feature maps (i.e., the image-like data) for deep learning models such as Convolutional Neural Networks models. However, for the traditional ML methods, their inputs are 1D-vectors instead of 2D feature maps. Therefore, there is a need to map unordered features for conventional ML models. By addressing this complementarity, the authors can underscore the importance of the feature order for traditional machine learning models with 1D vectors as inputs, and how their method fills this gap.
Secondly, I suggest that the authors revise Figure 1 to improve its clarity and make it easier for readers to understand. A well-designed figure will enhance the overall presentation of the manuscript.
Additionally, I recommend that the authors include a section titled "Theoretical Advantages of Mapping Transformation" in the Results section. This section should highlight the key ideas and theoretical benefits of their proposed approach, further elucidating the contributions of the paper.
Lastly, the authors should carefully revise the manuscript to ensure it meets the journal's formatting requirements and formats. Adhering to the journal's guidelines will enhance the professionalism and readability of the paper.
Considering these minor revisions, I believe the manuscript will be ready for publication. Therefore, I recommend that the authors address these points and make the necessary revisions. Once these revisions have been implemented, I suggest that the manuscript be reassessed for final acceptance.
Thank you for considering my review.

Methods

Are the methods appropriate to the aims of the study, are they well described, and are necessary controls included? Choose an item.

Conclusions

Are the conclusions adequately supported by the data shown? Choose an item.

Reporting Standards

Does the manuscript adhere to the journal's guidelines on minimum standards of reporting? Choose an item.

Choose an item.

Statistics

Are you able to assess all statistics in the manuscript, including the appropriateness of statistical tests used? Choose an item.

Quality of Written English

Please indicate the quality of language in the manuscript: Choose an item.

Declaration of Competing Interests

Please complete a declaration of competing interests, considering the following questions:

- Have you in the past five years received reimbursements, fees, funding, or salary from an organisation that may in any way gain or lose financially from the publication of this manuscript, either now or in the future?
- Do you hold any stocks or shares in an organisation that may in any way gain or lose financially from the publication of this manuscript, either now or in the future?
- Do you hold or are you currently applying for any patents relating to the content of the manuscript?
- Have you received reimbursements, fees, funding, or salary from an organization that holds or has applied for patents relating to the content of the manuscript?
- Do you have any other financial competing interests?
- Do you have any non-financial competing interests in relation to this paper?

If you can answer no to all of the above, write 'I declare that I have no competing interests' below. If your reply is yes to any, please give details below.

I declare that I have no competing interests

I agree to the open peer review policy of the journal. I understand that my name will be included on my report to the authors and, if the manuscript is accepted for publication, my named report including any attachments I upload will be posted on the website along with the authors' responses. I agree for my report to be made available under an Open Access Creative Commons CC-BY license (http://creativecommons.org/licenses/by/4.0/). I understand that any comments which I do not wish to be included in my named report can be included as confidential comments to the editors, which will not be published.

Choose an item.

To further support our reviewers, we have joined with Publons, where you can gain additional credit to further highlight your hard work (see: https://publons.com/journal/530/gigascience). On publication of this paper, your review will be automatically added to Publons, you can then choose whether or not to claim your Publons credit. I understand this statement.

Yes Choose an item.
