# Supplementary material for: Machine learning–based feature selection to search stable microbial biomarkers: application to inflammatory bowel disease
Source: Gigascience. 2023 Oct 26;12:giad083. doi: 10.1093/gigascience/giad083 (PMC10600917; doi:10.1093/gigascience/giad083)
Supplement: giad083_Reviewer_2_Report_Original_Submission — Jakob Wirbel -- 7/16/2023 Reviewed [file giad083_reviewer_2_report_original_submission.pdf]

**Reviewer Report**

**Title: Machine learning based feature selection to search stable microbial biomarkers: application to inflammatory bowel disease**

**Version: Original Submission** **Date:** 7/16/2023

**Reviewer name: Jakob Wirbel**

**Reviewer Comments to Author:**

I want to commend the authors on a much-improved manuscript. I fully appreciate that it must have been a tremendous amount of work to rerun everything with the log-transformation and to change the major display items.
I would like to raise two additional points, the first of which is of major importance:
1. I am a bit concerned about the numbers in Table 1. For example, for Dataset #1 (the data from the Lloyd-Price et al. paper), the authors report only a single non-IBD sample. In figure one of Lloyd-Price et al, there are multiple nonIBD samples (https://www.nature.com/articles/s41586-019-1237-9/figures/1). Can the authors explain this disparity? Also, the number of samples Dataset #2 (637) seems to be much higher than the number of subjects in the original study (137, see https://www.nature.com/articles/nmicrobiol20174#Sec1). Did the authors report the number of samples rather than the number of subjects? Lastly, the study from Flores et al. does not seem to be focussed on IBD at all. Am I missing something here or is there a mixup with the references?
2. I would recommend to order the features in Figure 5 according to their enrichment in IBD vs controls (most enriched in IBD on the top, most enriched in controls at the bottom, for example). (optional)

**Methods**

Are the methods appropriate to the aims of the study, are they well described, and are necessary controls included? Choose an item.

**Conclusions**

Are the conclusions adequately supported by the data shown? Choose an item.

**Reporting Standards**

Does the manuscript adhere to the journal's guidelines on minimum standards of reporting? Choose an item.

Choose an item.

**Statistics**

Are you able to assess all statistics in the manuscript, including the appropriateness of statistical tests used? Choose an item.

**Quality of Written English**

Please indicate the quality of language in the manuscript: Choose an item.

**Declaration of Competing Interests**

Please complete a declaration of competing interests, considering the following questions:

- Have you in the past five years received reimbursements, fees, funding, or salary from an organisation that may in any way gain or lose financially from the publication of this manuscript, either now or in the future?
- Do you hold any stocks or shares in an organisation that may in any way gain or lose financially from the publication of this manuscript, either now or in the future?
- Do you hold or are you currently applying for any patents relating to the content of the manuscript?
- Have you received reimbursements, fees, funding, or salary from an organization that holds or has applied for patents relating to the content of the manuscript?
- Do you have any other financial competing interests?
- Do you have any non-financial competing interests in relation to this paper?

If you can answer no to all of the above, write 'I declare that I have no competing interests' below. If your reply is yes to any, please give details below.

I declare that I have no competing interests

I agree to the open peer review policy of the journal. I understand that my name will be included on my report to the authors and, if the manuscript is accepted for publication, my named report including any attachments I upload will be posted on the website along with the authors' responses. I agree for my report to be made available under an Open Access Creative Commons CC-BY license (http://creativecommons.org/licenses/by/4.0/). I understand that any comments which I do not wish to be included in my named report can be included as confidential comments to the editors, which will not be published.

Choose an item.

To further support our reviewers, we have joined with Publons, where you can gain additional credit to further highlight your hard work (see: https://publons.com/journal/530/gigascience). On publication of this paper, your review will be automatically added to Publons, you can then choose whether or not to claim your Publons credit. I understand this statement.

Yes Choose an item.
